# Geometry-controlled instability pathway selection in elastic helices enables fast, efficient robotic locomotion

**DOI:** 10.1126/sciadv.aeh2779

**Published:** 2026-09-18

**Authors:** Dezhong Tong, Jiaqi Wang, Zexiong Chen, Andy Borum, Weicheng Huang, Xiaonan Huang, M. Khalid Jawed

**Affiliations:** ^1^ Robotics Department, University of Michigan, Ann Arbor, MI 48109, USA.; ^2^ Department of Mechanical and Aerospace Engineering, University of California, Los Angeles, CA 90095, USA.; ^3^ Mathematics and Statistics Department, Vassar College, Poughkeepsie, NY 12604, USA.; ^4^ School of Engineering, Newcastle University, Newcastle upon Tyne NE1 7RU, UK.

## Abstract

Mechanical instabilities can convert slow actuation into rapid, amplified motion, but predicting whether a three-dimensional elastic structure buckles smoothly or snaps remains challenging. Here, we show that helix geometry and boundary loading jointly select continuous buckling or snap-through in elastic filaments. Combining nonlinear rod theory, simulations, and high-throughput robotic arm experiments, we establish a predictive pathway map and identify snap-through as a robust mechanism for repeatable motion amplification under simple boundary actuation. Guided by this map, we design a snap-actuated helical limb that converts small end rotations into rapid global reconfiguration and cyclic energy release. Integrated into an untethered robot, this limb enables sustained hopping across wood, cloth, acrylic, leather, grass, and sand, reaching 3.21 body lengths per second with an electrical cost of transport of 4.79. Controlled comparisons with a rigid-legged robot show that snap-through drives the gains in speed, efficiency, and terrain robustness. The same limb architecture also enables agile turning, repeated back-flips, and swimming.

## INTRODUCTION

Mechanical instabilities provide a powerful route for converting low-amplitude actuation into rapid, amplified motion by storing elastic energy and releasing it abruptly across a stability boundary (*1*, *2*). In biological and engineered systems alike, such instabilities can generate large deformations, high power density, and rapid, forceful motion that would be difficult to achieve through direct actuation alone (*3*–*5*). Yet in three-dimensional elastic structures, postcritical behavior often depends sensitively on geometry, boundary conditions, and loading direction, making instability difficult to predict, control, and exploit systematically (*6*–*9*). Under loading, slender elastic structures can access distinct nonlinear deformation pathways, from continuous buckling to discontinuous snap-through, and pathway selection can change sharply with geometry and boundary loading (*10*–*13*). A general predictive framework that links geometry and boundary loading to instability-pathway selection, and converts that selection into a practical design rule for embodied function, remains largely absent.

A particularly useful setting for studying this question is provided by initially straight elastic filaments driven into helical configurations by boundary loading (*14*). In these systems, the induced helical geometry reshapes the accessible equilibria and stability structure, producing a mechanically rich postcritical response (*6*, *14*). Boundary-induced helices therefore provide a natural platform for uncovering how geometry and loading govern instability-pathway selection in slender elastic structures. This setting is also attractive for embodied robotic function. Unlike many instability-driven systems based on planar beams, shells, origami structures, or bilayers, boundary-induced helices intrinsically couple bending, twisting, and axial compression, thereby enabling large three-dimensional deformation and directional elastic energy release. Moreover, the helical state can be programmed from a naturally straight rod through boundary displacement and rotation, without fabricating an intrinsically curved or complex multistable architecture. This geometric programmability makes the mechanism compatible with simple motor-imposed boundary rotation, offering a route to compact, resettable, untethered actuation while reducing the need for complex distributed actuation or externally imposed deformation.

An important question is whether such pathway selection can be translated into a robust embodied function, rather than remaining only a mechanics phenomenon. Among candidate tasks, locomotion over irregular, deformable, or energy-dissipative terrain provides a stringent testbed for mechanically amplified behavior (*15*, *16*). Unlike continuous-contact gaits that rely on sustained and predictable frictional interaction, hopping concentrates propulsion into brief, high-power events and can reduce losses on yielding substrates (*17*). Recent robotic systems have successfully harnessed elastic instabilities for jumping (*18*, *19*), crawling (*20*–*22*), swimming (*23*, *24*), rolling (*25*), and shape morphing (*12*, *26*). These studies demonstrate that mechanical instabilities can be engineered into diverse embodied functions, often through specifically designed structures, fabricated architectures, or actuation sequences that exploit a chosen unstable response (*27*–*29*). In many such systems, however, the useful unstable response is selected through a particular fabricated geometry, material architecture, or empirically tuned actuation sequence. A complementary mechanics-guided design principle is still needed to predict, before robotic implementation, whether a slender elastic structure will undergo continuous buckling or energy-releasing snap-through and to translate that prediction into actuator design rules. This distinction is important because snap-through is only one possible outcome of instability. Even within boundary-induced elastic helices, changes in geometry and loading direction can switch the response between continuous buckling and energy-releasing snap-through. If the snap-through pathway is selected, the resulting helical structure can be triggered and reset through simple motor-imposed boundary rotation, without requiring complex distributed actuation. Locomotion therefore provides a demanding setting in which to test whether instability-pathway selection can be translated from a mechanics problem into robust embodied performance.

**Fig. 1.**
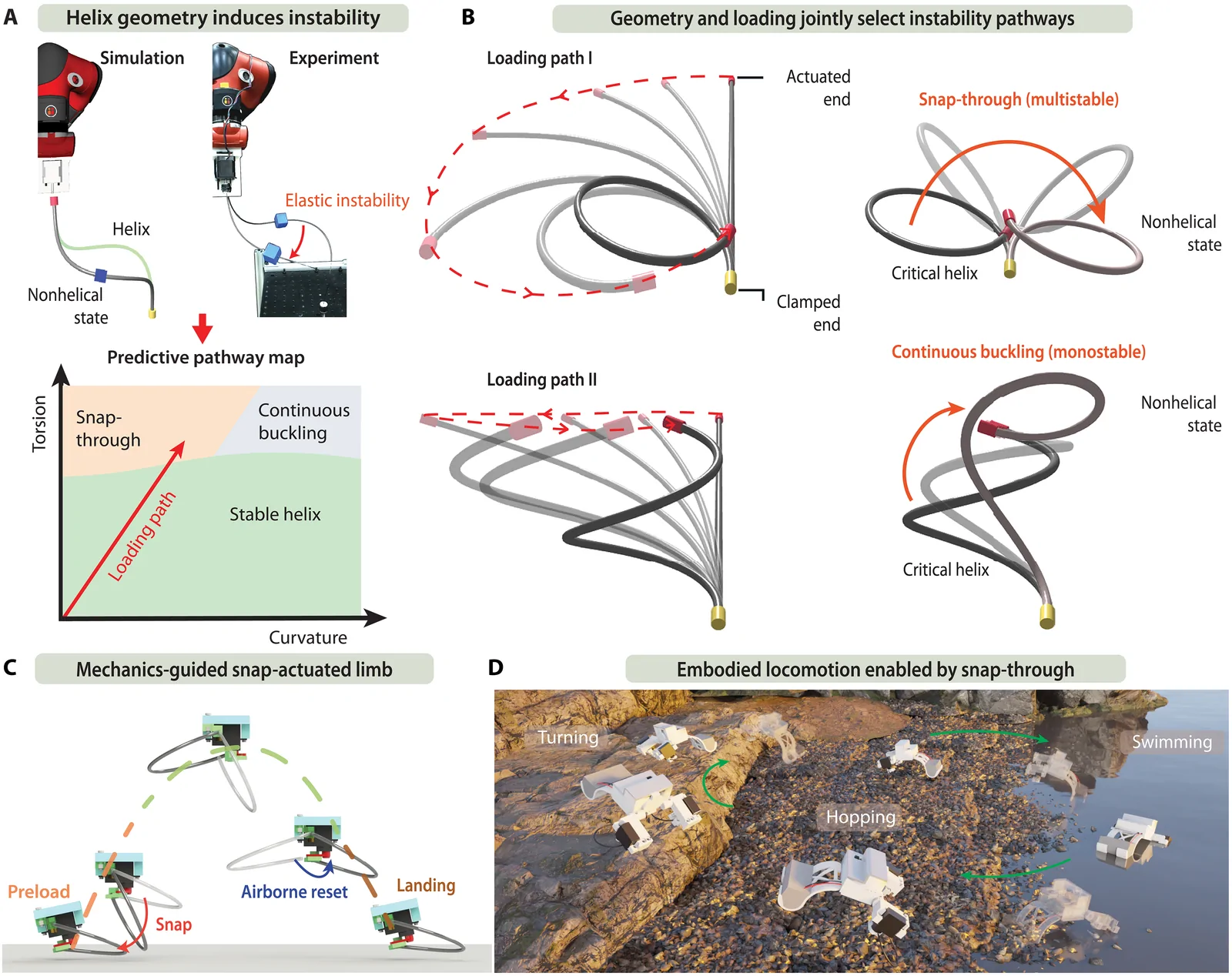
Instability-pathway selection in elastic helices enables snap-actuated locomotion. (**A**) Theory, simulation, and automated experiments show that boundary-induced helix geometry can lose stability, motivating a predictive map of instability pathways. (**B**) Geometry and loading jointly select two distinct responses of a critical helical state: snap-through, which is multistable and releases elastic energy, and continuous buckling, which is monostable and deforms smoothly. (**C**) Guided by this framework, we design a snap-actuated helical limb that converts small boundary actuation into rapid reconfiguration and impulsive mechanical output. (**D**) An untethered robot equipped with two such limbs exploits this mechanism for sustained hopping, with turning and swimming emerging as secondary manifestations of the same snap-actuated design rule.

Here, we establish a predictive framework for geometry-controlled instability-pathway selection in initially straight elastic filaments driven into helical configurations. This mechanics-to-function route is summarized in Fig. 1A. We show that helix geometry and boundary loading jointly select whether a critical helical state undergoes smooth continuous buckling or discontinuous snap-through (Fig. 1B) and identify snap-through as the mechanically useful pathway because it provides large, repeatable elastic energy release under simple boundary control. Combining nonlinear rod theory, discrete elastic rod (DER) simulations, and high-throughput robotic arm experiments, we construct a predictive map of instability pathways and associated energy release. We then convert this map into a practical design rule for a resettable snap-actuated helical limb that transforms a modest end rotation into rapid global reconfiguration (Fig. 1C). Integrated into an untethered robot, this mechanics-guided actuator enables sustained hopping across diverse terrains with high speed and low cost of transport (CoT) (Fig. 1D). Controlled comparisons with a rigid-legged counterpart across diverse terrains, including wood, acrylic, leather, cloth, grass, and sand, further show that snap-through actuation is the primary source of the gains in speed, efficiency, and terrain robustness. The same mechanism also enables agile turning, repeated back-flipping, and swimming as secondary manifestations of the same snap-through design rule. Together, these results establish instability-pathway selection in slender elastic structures, demonstrated here in boundary-induced elastic helices, as a predictive route from mechanics to programmable mechanical amplification and embodied locomotion.

## RESULTS

### Instability-pathway selection in elastic helices

**Fig. 2.**
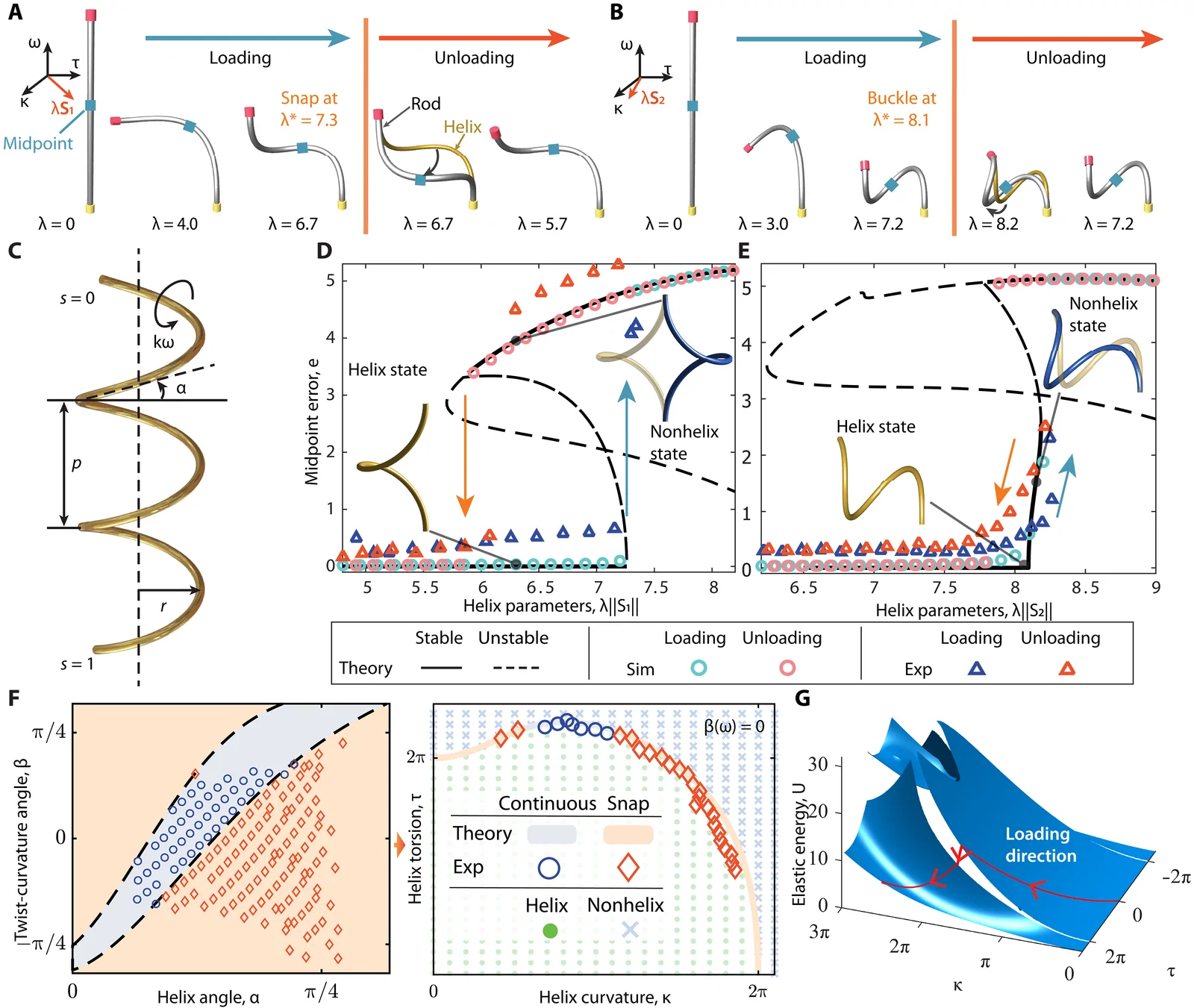
Instability pathways in elastic helices mapped by theory, simulation, and automated experiments. Loading-unloading along rays in boundary-condition space (κ, τ, ω) selects either snap-through (**A**) or continuous buckling (**B**); the two representative cases are **S**_1_ = [0.704, 0.700, 0.120] and **S**_2_ = [0.901, 0.407, 0.148]. (**C**) Helix geometry set by curvature κ and torsion τ, with radius $r=\kappa /\left(\kappa^{2}+\tau^{2}\right)$ and pitch $p=2\pi \tau /\left(\kappa^{2}+\tau^{2}\right)$. Midpoint error *e* distinguishes snap-through (**D**) from continuous buckling (**E**), with theory, simulation, and experiments in close agreement. (**F**) Phase diagram and regime classification in (α, β) space comparing theory and experiments. (**G**) Normalized elastic energy landscape at ω = 0, showing energy storage and release during snap-through.

Boundary-induced helical configurations can lose stability under load in two qualitatively different ways: discontinuous snap-through or smooth continuous buckling (movie S1). Representative loading-unloading sequences showing these two pathways are shown in Fig. 2 (A and B). For the mechanism studied here, this pathway selection is the key scientific question, because only snap-through provides the rapid reconfiguration and elastic energy release needed for strong mechanical amplification. We therefore seek a predictive framework that links boundary loading and induced helix geometry to instability-pathway selection (fig. S1).

We start from a straight clamped-clamped rod and deform it quasi-statically by prescribing the translation and rotation of one end while holding the other end fixed. As the loading amplitude λ increases, the rod passes through a family of helical equilibria parameterized by three nondimensional boundary-condition variables: curvature κ, torsion τ, and twist moment ω (Fig. 2C and section S1). We parameterize the approach to instability by rays in (κ, τ, ω) space

$$\left(\kappa ,\tau ,\omega \right)=\lambda S\left(\alpha ,\beta \right),S\left(\alpha ,\beta \right)=\frac{1}{\sqrt{1+{\text{tan}}^{2}\alpha +{\text{tan}}^{2}\beta }}\left[1,\text{tan}\alpha ,\text{tan}\beta \right]$$ (1)

where $\alpha ={\text{tan}}^{-1}\left(\tau /\kappa \right)$ defines the helix angle (Fig. 2C) and $\beta ={\text{tan}}^{-1}\left(\omega /\kappa \right)$ defines a twist-curvature angle. Thus, **S** specifies the loading direction, while λ measures the loading amplitude along that direction. For each loading direction, there exists a critical value $\lambda^{*}\left(S\right)$ at which the helical equilibrium loses stability and transitions to a nonhelical configuration.

To resolve this pathway-selection problem, we combine Kirchhoff-rod theory, high-fidelity DER simulations (*30*–*32*), and automated experiments (sections S1 to S3). The two representative loading directions in Fig. 2 (A and B) are **S**_1_ and **S**_2_. To quantify departure from the ideal helical state, we track a fiducial midpoint marker and define a normalized midpoint error *e* (defined in eq. S15), which yields the load-response curves in Fig. 2 (D and E). Along **S**_1_, the rod undergoes snap-through at $\lambda^{*}\approx 7.3$, with distinct loading and unloading branches and a hysteretic transition between helical and nonhelical states. Along **S**_2_, by contrast, the filament follows a smooth and reversible postcritical path and exhibits continuous buckling at $\lambda^{*}\approx 8.1$. Theory, simulation, and experiment show close agreement in both the critical point and the postcritical evolution.

We rationalize these two responses through bifurcation structure. Over the loading directions explored here, the loss of stability is organized by a pitchfork bifurcation (section S1). Subcritical pitchforks do not admit a continuously connected stable equilibrium branch beyond $\lambda^{*}$ and therefore produce snap-through, whereas supercritical pitchforks generate a stable postbuckled branch that emerges continuously from the helix and therefore yield continuous buckling. Thus, for the class of boundary-induced helices studied here, instability-pathway selection is governed by the loading direction **S**, which determines how the critical state is approached.

Sweeping the loading direction S(α,β) produces the phase diagram in Fig. 2F, which delineates regions of snap-through and continuous buckling and provides a predictive map from boundary loading and induced helix geometry to instability pathway. This pathway map is the key mechanics advance: It shows that helical buckling is not a single postcritical response but a geometry- and loading-selected transition between smooth continuous buckling and energy-releasing snap-through. Because this framework is formulated in nondimensional boundary-condition space, the resulting map is scalable across sizes. For isotropic rods, material properties do not alter the critical boundary-condition map in this formulation, although they can affect the bifurcation type (fig. S2). The associated energy landscape (Fig. 2G) further reveals a pronounced energy gap in the snap-through regime, corresponding to elastic energy stored before instability and released during rapid reconfiguration. In some parameter regimes, additional saddle-node bifurcations arise further along the postbuckling branches; although captured by our framework, these events are not exploited in the robotic design considered here (fig. S3). Together, these results establish the predictive instability-pathway map that we next convert into a resettable snap-actuated limb design.

### Predictive pathway mapping yields a resettable snap-actuated limb design

**Fig. 3.**
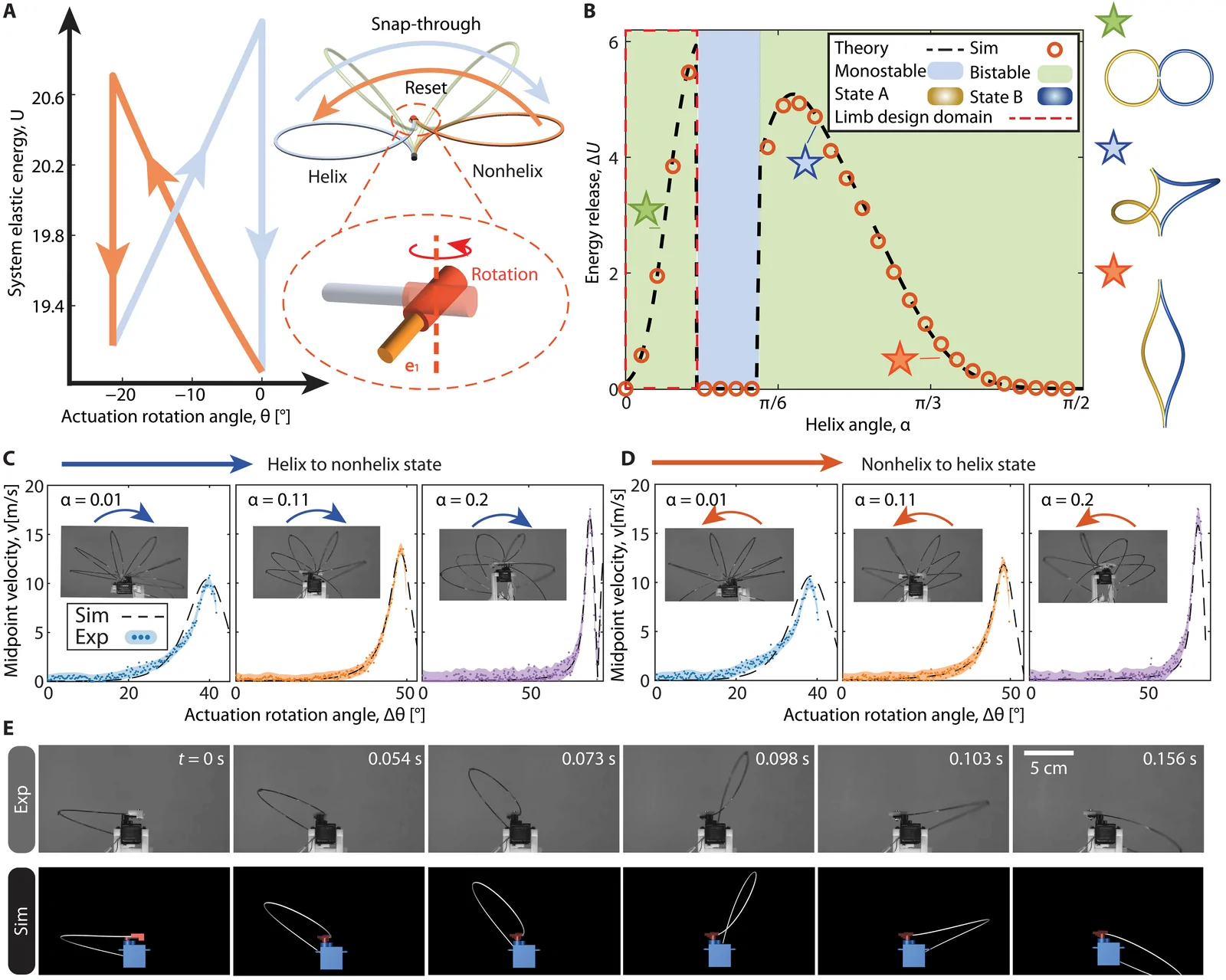
Design of the snap-actuated elastic helical limbs. (**A**) One full actuation cycle of the snap-actuated limb (helix angle α = 0.11); the zero of the rotation angle θ is defined at the critical configuration. (**B**) Normalized elastic energy release during snap-through for limbs with different critical helical shapes at zero end-twist. Experiment-simulation comparison of midpoint velocity over a full cycle: (**C**) helix-to-nonhelix transition and (**D**) nonhelix-to-helix transition. (**E**) Representative snapshots comparing experiments and dynamic simulations (helix angle α = 0.11).

Having established a predictive map for instability-pathway selection, we next convert that map into a practical design rule for robotic actuation. The design objective is not merely to trigger snap-through but to identify a resettable operating regime that combines substantial elastic energy release with simple boundary control and rapid repetition. We therefore seek a boundary-induced helical configuration that can repeatedly traverse the snap-through regime while remaining suitable for fabrication, integration, and cyclic use in a mobile robot. We implement this concept using a commercially available superelastic Nitinol rod with zero end-twist ω=0, with one end fixed to a three-dimensional printed holder and the other coupled to a micro-servo through a pushrod connector (fig. S7). During actuation, the servo applies a single-axis end rotation that implements a practically simplified loading path derived from the stability analysis; although reduced from the full theoretical boundary-loading space, this actuation preserves the essential conditions required for repeatable transitions between helical and nonhelical states (Fig. 3A and fig. S8). Despite this reduced one-degree-of-freedom implementation, the limb reliably reproduces the targeted snap-actuated cycle and is therefore suitable as a compact robotic actuator.

We first screen candidate designs by evaluating the normalized elastic energy (defined in eq. S6) released at instability as a function of helix angle $\alpha ={\text{tan}}^{-1}\left(\tau /\kappa \right)$, which parameterizes the family of critical helical shapes at zero end-twist (Fig. 3B). This screening immediately excludes the interval α∈(0.24,0.45), where the energy release at the stability threshold is nearly zero. In this regime, the helix undergoes continuous buckling and remains effectively monostable, making it unsuitable for high-power snap actuation. By contrast, pronounced energy release peaks appear near α≈0.24 and α≈0.57, identifying bistable critical shapes that are promising candidates for impulsive actuation. Strong energy release alone, however, is not sufficient for a practical limb. Larger helix angles near α≈0.57 produce strongly asymmetric three-dimensional postsnap shapes, which complicate fabrication, alignment, and reliable ground contact. We therefore restrict the design space to smaller helix angles, α∈ [0, 0.24).

Within this reduced design space, we evaluate the trade-off between snap-through strength and actuation cost by fabricating limbs with three representative critical shapes, α=0.01, 0.11, and 0.20 and comparing midpoint-velocity profiles from experiments and dynamic simulations over a full actuation cycle (Fig. 3, C to E; fig. S9; and movie S2). Both approaches show the same trend: Increasing α not only raises the peak midpoint velocity during snap-through, indicating greater elastic energy release, but also requires a larger servo rotation Δθ to switch between stable states, increasing actuation and reset time. This reset cost is especially important for locomotion, because although much of the return stroke can occur while the robot is airborne, a shorter required actuation window enables faster cyclic operation and leaves more of each stride available for useful body motion. The resulting trade-off leads to an intermediate optimum at α=0.11, which is also identified in the supplementary analysis as maximizing released energy per actuation time (fig. S10). The design rule is therefore to select twist-free critical helices in the snap-through regime, exclude monostable and strongly asymmetric configurations, and choose the helix angle that maximizes usable energy release per required actuation and reset angle. This criterion yields α=0.11, which we use throughout the remainder of the paper as the operating point for the snap-actuated helical limb. Because snap-through is discontinuous, repeatable operation requires commanding the boundary path rather than tracking the rapid transition itself. In the implemented limb command, the servo drives the actuated end beyond the snap-through target angle, holds for a prescribed dwell period to allow completion of the transition and decay of postsnap vibration, and then reverses to reset the limb (section S4 and fig. S11). Limb-level finite-speed simulations and experiments show that finite-speed actuation does not suppress the selected snap-through pathway over the tested range but modulates its dynamic expression by changing the peak midpoint velocity and postsnap oscillatory response (section S4 and fig. S12). A timing analysis and a reliability test over 1200 consecutive cycles further show that this low-bandwidth command strategy produces repeatable snap-through and reset under the tested protocol (fig. S11 and movie S3).

### Mechanics-guided snap-through actuation enables untethered hopping

**Fig. 4.**
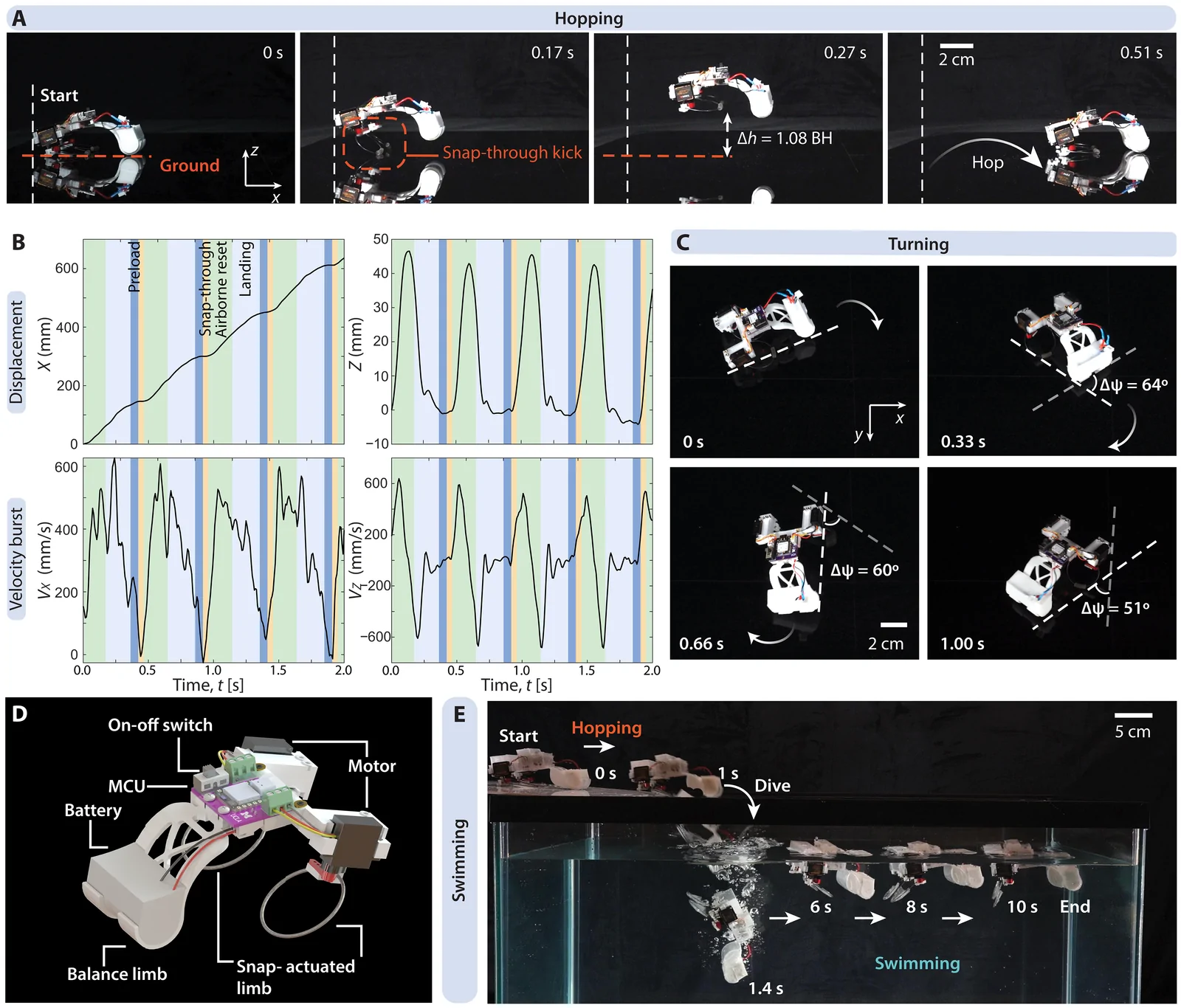
Snap-actuated helical limbs generate burst-like propulsion for sustained untethered hopping. (**A**) Time-lapse images of a representative hopping cycle show limb preloading, snap-through against the substrate, ballistic flight, and airborne reset before touchdown. (**B**) Tracked hopping kinematics show forward and vertical displacements, *x* (*t*) and *z* (*t*), together with the corresponding velocities, *v_x_* (*t*) and *v_z_* (*t*). Sharp peaks in both velocity components reveal the burst-like propulsion generated by snap-through during each hopping cycle. (**C**) Differential limb actuation enables agile turning. (**D**) Untethered prototype with two snap-actuated helical limbs, powered by an onboard LiPo battery and two servos. (**E**) The same limb architecture can transition from land to water, enabling hopping on land and swimming in water.

We next implement the mechanics-guided limb design in an untethered robotic platform (fig. S16A) to test whether the resettable snap-through cycle predicted by the instability-pathway framework can generate sustained locomotion. As shown in Fig. 4A, upon activation, the robot performs consecutive forward hops at an actuation cycle frequency of $f_{\text{act}}=2.13\;\text{Hz}$ (defined in eq. S48). Each cycle consists of limb preloading, snap-through against the substrate, ballistic flight, airborne reset, and touchdown. This sequence separates actuation and reset in time and establishes sustained hopping as the primary embodied consequence of the snap-through design. A robot-level actuation-frequency sweep further quantifies how command timing affects hopping performance, showing that stable hopping depends on the burst-like snap-through response and the available flight phase for limb reset (fig. S18).

Tracked hopping kinematics show that this gait is driven by burst-like propulsion generated during snap-through (Fig. 4B). The forward and vertical displacements, x(t) and z(t), together with the corresponding velocities, $v_{x}\left(t\right)$ and $v_{z}\left(t\right)$, exhibit sharp peaks during each cycle, indicating rapid release of elastic energy into both forward and upward motion. Using this mechanism, the platform reaches a peak speed of 3.21 ± 0.10 body lengths per second and a hop height of approximately 1.08 body heights. These results confirm that the same limb-level design logic—slow motor loading, rapid snap-through energy release, and low-loss aerial reset—translates directly into sustained untethered hopping.

The same limb-level mechanism also supports additional locomotion modes as secondary manifestations of the same design rule. Differential control of the two limbs enables agile turning (Fig. 4C), with an average yaw rate of 163° ± 12.5° per second, corresponding to a 90° turn in 0.55 s and a 180° turn in 1.1 s; arc turns achieve a minimum turning radius of *R*_min_ ≈ 3.22 cm or 0.29 body lengths (fig. S19A). These behaviors are realized on a compact, power-autonomous robot measuring 11 cm by 8 cm by 4 cm and carrying two independently driven snap-actuated helical limbs, two micro-servos, an onboard 7.4-V LiPo battery, and a lightweight custom control board (Fig. 4D and section S5). The total mass is 98.2 g, corresponding to a gravitational load of ∼0.96 N, which is well below the combined limb-level blocking force scale of 6.56 N measured for the two limbs (fig. S14). This force margin is consistent with the observed body lift during stance and with the robot-level reaction force measurements discussed below.

A sealed enclosure further enables the same robot to transition from land to water and swim at approximately 0.5 body lengths per second (Fig. 4E and fig. S20). Additional exploratory aquatic locomotion experiments show that swimming performance is tunable through actuation cycle frequency and limb installation angle, while swimming-specific optimization of limb geometry, body morphology, and fluid-structure interaction remains an important direction for future work (section S5 and fig. S21). Related timing variants also support repeated back-flips, reaching heights of up to 3.2 body heights per flip (section S5 and fig. S22). Together, these results establish sustained hopping as the primary embodied consequence of snap-through actuation while showing that the same limb architecture can be repurposed for turning, swimming, and other dynamic maneuvers (movie S4).

### Snap-through actuation improves locomotion across diverse terrains

Having established sustained untethered hopping, we next isolate the contribution of snap-through actuation to robot locomotion performance. To do so, we compare the snap-actuated robot against a rigid-legged control in which the snap-actuated elastic helical limbs are replaced by rigid counterparts while the rest of the untethered platform architecture is held fixed (fig. S16). To ensure a controlled comparison, the two robots used the same body architecture, onboard battery, control board, micro-servos, holder inclination, effective limb length from the servo output-shaft center to the friction pad ground-contact point, contact pad material, and substrate set; the only intended design difference was replacing the snap-actuated elastic helical limbs with rigid printed counterparts (table S1). Both robots were driven using the same prescribed servo command sequence at the same actuation cycle frequency, *f*_act_ = 2.13 Hz. The robot masses were similar, with the snap-actuated robot weighing 98.2 g and the rigid-legged control weighing 92.6 g. This controlled comparison therefore tests whether the instability-selected, resettable snap-through actuation mechanism, rather than simply the presence of a lightweight hopping body, is responsible for the observed gains in speed and terrain adaptability.

**Fig. 5.**
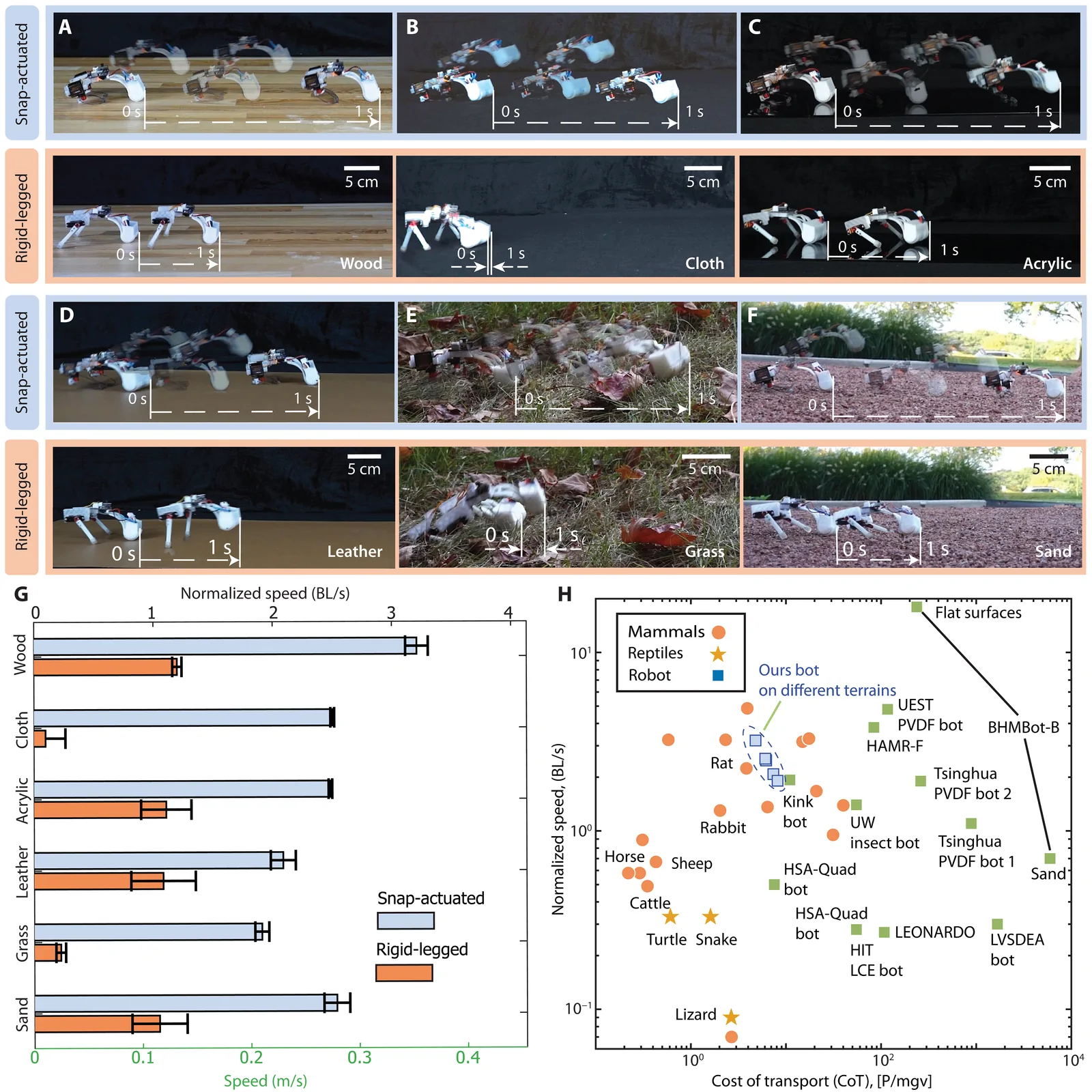
Controlled terrain benchmarking isolates the benefit of snap-through actuation. (**A** to **F**) Time-lapse snapshots comparing the snap-actuated hopping robot and a rigid-legged control on six representative terrains: (A) wood, (B) cloth, (C) acrylic, (D) leather, (E) grass, and (F) sand. (**G**) Forward speed of the snap-actuated hopping robot and the rigid-legged control across these terrains. (**H**) Body length–normalized speed and minimum electrical CoT of our robot relative to animals and previously reported untethered terrestrial robots. Data sources are listed in tables S3 to S6.

Across all six tested terrains, the snap-actuated robot consistently outperforms the rigid-legged control in forward speed (Fig. 5, A to G, and movie S5). The snap-actuated robot achieves average speeds of 3.21, 2.50, 2.48, 2.09, 1.91, and 2.54 body lengths per second on wood, cloth, acrylic, leather, grass, and sand, respectively, whereas the rigid-legged control reaches only 1.20, 0.10, 1.11, 1.08, 0.22, and 1.05 body lengths per second on the same terrains. Averaged across all six terrains, the snap-actuated platform reaches 2.46 body lengths per second, compared with 0.79 body lengths per second for the rigid-legged control. This consistent advantage shows that the locomotion gains arise from snap-through actuation itself rather than from the robot body alone.

Electrical input was characterized under matched command conditions using a fixed 7.4-V supply, corresponding to the nominal voltage of the onboard 2S LiPo battery, with current measured in real time to compute *P*(*t*) = *V I*(*t*) (fig. S24). The snap-actuated robot drew an average electrical power of 1.63 W, compared with 1.41 W for the rigid-legged control. Thus, the improved locomotion performance is not simply explained by a large increase in average electrical input but is associated with elastic energy storage and rapid release in the snap-through limbs. Additional robot-level reaction force measurements further show that, under matched actuation conditions, the snap-actuated robot generates a larger and more rapid vertical force burst than the rigid-legged control: The peak raw vertical reaction force increases from ∼1.53 to 2.46 N, and the net vertical impulse increases from 0.027 to 0.106 N·s (fig. S25C).

The advantage is especially pronounced on deformable and heterogeneous terrains. On rigid, smooth substrates such as wood, both robots remain mobile, although the snap-actuated design is still faster. On cloth and grass, however, the rigid-legged control nearly stalls, whereas the snap-actuated robot maintains relatively fast and stable locomotion. This robustness follows directly from the propulsion mechanism: Motor-driven preloading stores elastic energy in the helical limbs and then snap-through releases it rapidly in a brief, high-power stroke, while the ensuing ballistic phase reduces sustained interaction with dissipative or irregular substrates. By contrast, the rigid-legged control relies more heavily on continuous stance contact and is therefore more sensitive to slipping, sinkage, and terrain-induced energy loss. Both robots used the same contact pad material on the same substrate set, and friction measurements are provided in the Supplementary Materials (table S2). These measurements define the terrain interaction conditions and further support the reduced terrain sensitivity of the snap-actuated robot relative to the rigid-legged control.

We further assess energetic performance through the minimum electrical CoT, defined by eq. S51, and compare the robot with terrestrial animals and previously reported untethered robots (Fig. 5H). The snap-actuated robot achieves a minimum CoT of 4.79, combining high speed with low transport cost. Figure 5H is intended as contextual benchmarking rather than a primary claim, but it shows that the present robot occupies a favorable region of the speed-efficiency space relative to prior untethered terrestrial platforms. Together, these controlled comparisons establish snap-through actuation as the primary source of the observed gains in speed, efficiency, and terrain robustness and motivate the final unstructured environment tests in the next section.

### Robust operation in unstructured terrestrial and aquatic environments

**Fig. 6.**
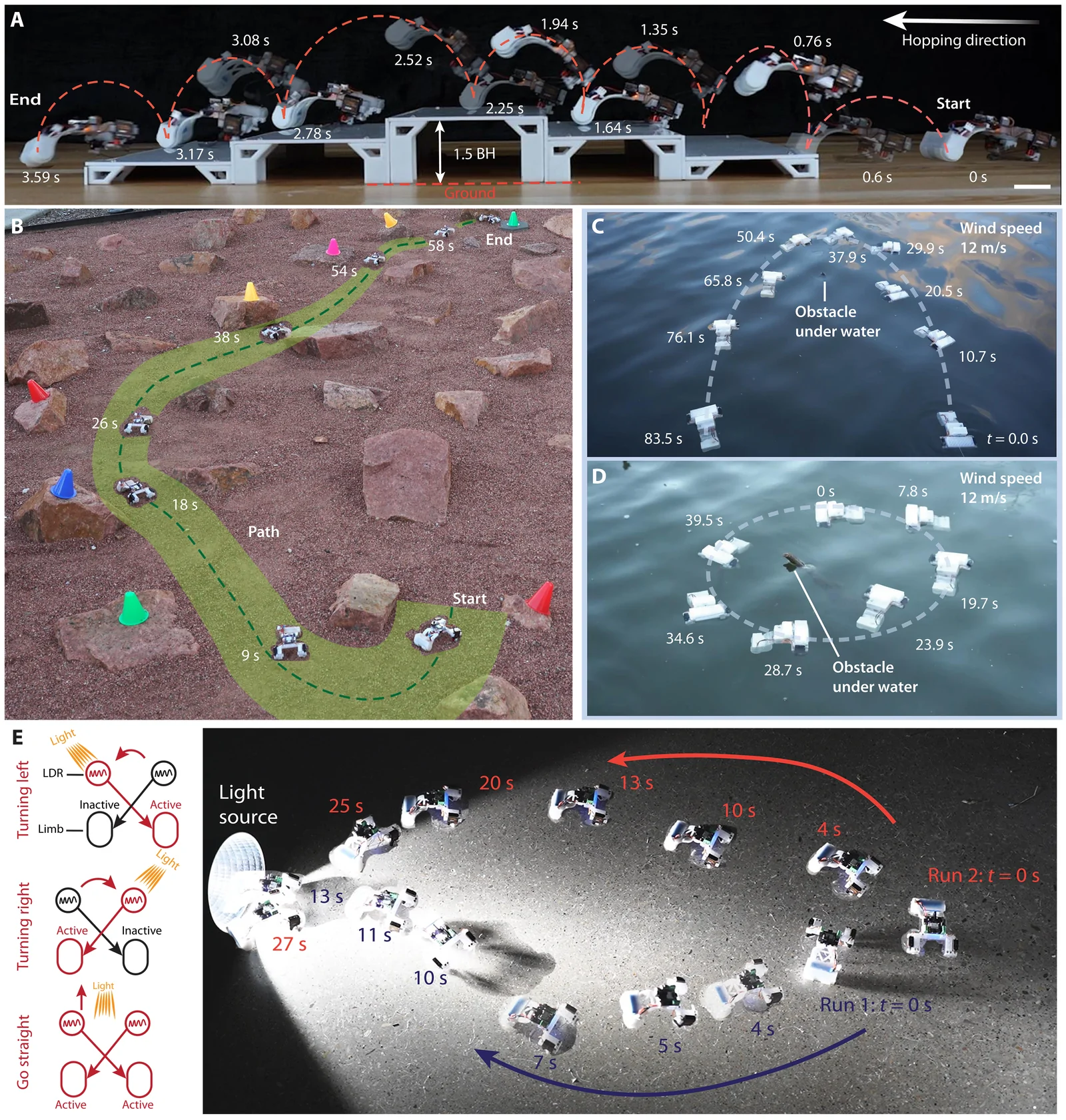
Robust operation in unstructured terrestrial and aquatic environments. (**A**) Stair climbing and descending on a constructed staircase. (**B**) Path tracking on outdoor sand with discrete obstacles. (**C** and **D**) Swimming in a natural outdoor pool with underwater obstacles under wind disturbance. (**E**) Demonstration of simple light-guided steering using onboard light-dependent resistors (LDRs).

Beyond controlled benchmarks, we lastly test whether the same mechanics-guided snap-actuated limb design remains effective in less structured settings that combine geometric discontinuities, substrate heterogeneity, and environmental disturbances. Using the same snap-actuated limb architecture, with task-specific packaging when needed, we examine locomotion on stairs, outdoor sand with discrete obstacles, and swimming in a natural outdoor pool. We additionally test whether the snap-actuated platform can be integrated with simple onboard sensing through a light-guided steering demonstration. The purpose of this section is not to introduce an additional actuation principle but to validate the breadth of the earlier design rule by showing that the same resettable snap-actuated limb remains effective outside laboratory-like conditions. We first demonstrate stair climbing and descending on a constructed staircase (Fig. 6A and movie S6). The robot repeatedly clears step edges and recovers from landing misalignments while maintaining forward progression, showing that snap-through-driven hopping can tolerate geometric discontinuities that would challenge gaits based on sustained ground contact. We next evaluate locomotion on outdoor sand interspersed with small rocks (Fig. 6B and movie S7), representing a heterogeneous, deformable, and dissipative terrain. Under teleoperation (fig. S26), the robot follows a prescribed path while maintaining stable hopping despite localized yielding and obstacles.

We then demonstrate aquatic locomotion in a natural outdoor pool (Fig. 6, C and D, and movie S8). In this setting, periodic snap-through of the elastic limbs drives oscillatory body motions that generate thrust in water, while a sealed enclosure protects the onboard components. Together, these experiments provide breadth validation of the earlier design rule by showing that the same snap-actuated limb architecture supports robust operation across built structures, loose outdoor terrain, and a natural outdoor aquatic setting.

To demonstrate compatibility with onboard sensing and feedback control, we further equipped the robot with onboard light-dependent resistors to switch left/right limb activation in response to the relative direction of a light source (Fig. 6E and movie S9). Using this simple feedback rule, the robot reoriented and moved toward the same light source from different initial poses, showing that the snap-actuated limb architecture can also be integrated with lightweight sensing and feedback control.

## DISCUSSION

In this work, we establish a predictive framework for instability-pathway selection in boundary-induced elastic helices and show how it can be converted into a practical design rule for robotic actuation. By combining nonlinear rod theory, DER simulations, and high-throughput automated experiments, we map the conditions under which a critical helical state follows continuous buckling or discontinuous snap-through and show that snap-through provides rapid elastic energy release for mechanical amplification under simple boundary control. We then translate this pathway map into a resettable snap-actuated helical limb that converts relatively small boundary actuation into rapid global reconfiguration while supporting cyclic operation. Integrated into an untethered robot, this actuator enables sustained hopping, while controlled comparisons with a rigid-legged counterpart across rigid, deformable, and granular substrates show that the gains in speed, efficiency, and terrain robustness arise from the instability-driven actuation mechanism rather than from body architecture alone. Turning, back-flipping, swimming, and operation in unstructured environments further illustrate how the same snap-through design rule supports a broader range of embodied behaviors.

The practical value of this mechanism lies in the way robotic output is generated. Geometry and boundary loading select an energy-releasing snap-through pathway, allowing a compact motor to load elastic energy gradually through boundary rotation and release it rapidly through a resettable elastic instability. Because a helix is an equilibrium configuration of a naturally straight elastic rod under boundary constraints, three-dimensional energy-storing limbs can also be fabricated and programmed without complex multicomponent mechanisms. This motor-elastic architecture can serve as a mechanical amplification module for robots in which direct-drive peak output, instantaneous power delivery, size, or weight is limiting. Rather than requiring the motor to directly generate the full burst output, this architecture allows the motor to load elastic energy over a longer boundary actuation phase, while the helical limb releases that energy over a much shorter snap-through event. Such a strategy is particularly relevant for systems requiring intermittent high-power actions, including hopping over obstacles, escaping from deformable or dissipative terrain, impulsive turning and reorientation, amphibious propulsion, and compact deployable appendages. In this sense, instability-pathway selection provides not only a way to explain nonlinear elastic transitions but also a route to program when and how stored elastic energy is released for robotic function.

The present study focuses on a specific class of boundary-induced helical configurations and robot-scale implementations driven by simple rotational actuation. The same mechanism also introduces practical trade-offs: The output is burst-like rather than continuously variable, the limb must be reset after each snap-through event, and the snap/reset timing should be coordinated with body motion and environmental contact. Environmental interactions can further shape how the selected snap-through event is expressed by introducing contact constraints, frictional dissipation, substrate deformation, or fluid loading. Future work could extend this framework to broader elastic architectures and material systems and develop whole-robot simulations that couple snap-actuated structures with body dynamics, contact, substrate deformation, fluid loading, and control. Such models would enable snap-through geometry, boundary loading, command timing, and body morphology to be codesigned for different embodied functions. Together, these results establish instability-pathway selection in slender elastic structures as a predictive route from nonlinear mechanics to resettable actuator design, embodied locomotion, and programmable mechanical amplification.

## MATERIALS AND METHODS

For brevity, the snap-actuated helical elastic limb is referred to as the “limb” and the locomoting platforms as the “robots.”

### Theory and stability analysis

The filament is modeled as an isotropic, inextensible, unshearable Kirchhoff rod with clamped boundary conditions. After nondimensionalization, helical equilibria are parameterized by curvature κ, torsion τ, and twist moment ω. Linear stability is evaluated with the conjugate point criterion by integrating eq. S9 over s∈(0,1] using the helical strains, forces, and moments from eq. S8; stability requires $\text{det}\left[J\left(s\right)\right]\neq 0$ for all s∈[0,1]. Nonhelical postbuckling equilibria are computed by a shooting-based Newton-Raphson method applied to eq. S2 under clamped boundary conditions prescribed by a selected helical state and continued by pseudo-arclength continuation. Stability along postbuckling branches is assessed using the same conjugate point criterion. Details are provided in the Supplementary Materials.

### DER simulations

Simulations are performed with the DER formulation (*30*–*32*), in which the centerline is discretized into nodes and edges with associated reference and material frames. Stretching, bending, and twisting energies define the internal forces, and the equations of motion are advanced using an implicit Euler scheme solved by Newton-Raphson iterations. To probe pathway selection in (κ, τ, ω) space, clamped boundary conditions corresponding to a helical reference configuration λ**S** are prescribed, and λ is varied quasi-statically until an instability occurs. Instability type is identified from the normalized midpoint error *e* together with loading-unloading hysteresis. Environmental interactions can be incorporated in the same DER framework through the external force term, including frictional contact and hydrodynamic loading; the formulations and a contact-constrained blocking force example are provided in the Supplementary Materials. Unless otherwise stated, DER simulations used the material parameters of the experimental Nitinol rods, with Young’s modulus *E* = 33 GPa, Poisson’s ratio ν = 0.33, and density ρ = 6450 kg m^−3^; the geometry, boundary conditions, and numerical settings were matched to each corresponding theoretical, limb-level, or robotic study, with details provided in the Supplementary Materials.

### Automated stability experiments

An automated testbed was developed to impose helical boundary conditions on a superelastic Nitinol wire (length, 0.5 m; McMaster-Carr 8320K42) and map instability outcomes along directions **S** in (κ, τ, ω) space. Endpoint position and the zero-twist intermediate frame are prescribed by a 7-degree-of-freedom robot arm (Sawyer, Rethink Robotics), while end twist about the local tangent is imposed by a customized end effector with a NEMA 17 stepper motor. Collision- and singularity-free trajectories are generated using Descartes (*33*). The filament midpoint is tracked with an RGB-D camera (Intel RealSense D435) and compared with the corresponding helical reference to compute midpoint error. Snap-through and continuous buckling are classified from the postbuckled error response using clustering-based criteria that account for measurement noise and finite sampling rate.

### Limb and robot design

Each limb consists of a superelastic Nitinol rod, a rigid holder, and printed end connectors that enforce a prescribed twist-free critical helix ω = 0 with target (κ, τ). The holder sets the end-to-end displacement and terminal tangents of the target configuration according to eqs. S37 and S38, with one end fixed and the other driven by a micro-servo (EMAX ES9052MD HV) to impose end rotation. Unless otherwise noted, experiments use α = 0.11, *L* = 13.5 cm, and *r*_0_ = 0.4 mm. The filament is superelastic Nitinol (uxcell), the holder is printed in polycarbonate (Bambu Lab PC filament) on a Bambu Lab X1C, and the end connectors are printed in photopolymer resin (Formlabs White Resin V5) on a Form 4.

The untethered hopping robot adopts a modular architecture with two limb modules mounted beneath the body at a fixed 10° inclination. The body is printed in polycarbonate on a Bambu Lab X1C, the balance limb is printed in Formlabs White Resin V5 on a Form 4, and the friction pads are printed in compliant resin (Formlabs Elastic 50A Resin V2). A custom driving circuit and onboard 2S LiPo battery (400 mAh, 7.4 V; Crazepony) are integrated into the platform. A rigid-legged baseline is constructed by replacing the elastic limbs with rigid printed limbs while preserving the same body, electronics, battery packaging, holder inclination, and actuation signals. For aquatic experiments, an amphibious variant incorporates buoyancy, waterproof sealing, and thin flexible films (VHB 4905, 3M) attached to the limbs to increase thrust while preserving snap-through. For controlled terrain comparisons, the snap-actuated and rigid-legged robots were driven using the same prescribed loading-dwell-reset-dwell command sequence at *f*_act_ = 2.13 Hz.

### Measurements

Snap-through dynamics are recorded with a high-speed camera (Wave) equipped with a 7artisans Photoelectric 60-mm f/2.8 Macro lens and illuminated by a Godox SL60IIBI Bi-Color LED Video Light at 2955 frames per second and a resolution of 2048 × 512 pixels. Robot trajectories and locomotion speeds are extracted from side-view videos (120 frames per second; Sony α7 III with a Tamron 28- to 75-mm f/2.8 lens) using Tracker, with an in-plane calibration scale used to convert pixels to distance and a consistent body-fixed point used to compute displacement, speed, and orientation. Blocking forces were measured on a tensile testing machine (Instron 68SC-5) with a static load cell (Instron 2530-100N) in displacement control. Robot-level vertical reaction forces were measured by operating the robot on an acrylic substrate mounted on a load cell (SBT641, SIMBATOUCH), and effective robot-substrate friction was characterized using static tilt angle tests and kinetic force gauge pulling tests; detailed protocols are provided in the Supplementary Materials. Electrical input power was obtained from the regulated supply voltage and total current draw of the robot using an inline current/voltage sensor (INA219, Adafruit) streamed to a microcontroller (Arduino Uno); instantaneous power was computed as *P*(t) = *V I*(t) and averaged over the measurement interval for cost-of-transport calculations.

## Supplementary Materials

### The PDF file includes:

Supplementary Text Figs. S1 to S28 Tables S1 to S6 Legends for movies S1 to S9 References
