# Supplementary material for: Geometry-controlled instability pathway selection in elastic helices enables fast, efficient robotic locomotion
Source: Sci Adv. 2026 Sep 18;12(38):eaeh2779. doi: 10.1126/sciadv.aeh2779 (PMC13588192; doi:10.1126/sciadv.aeh2779)
Supplement: Supplementary file 1 [file sciadv.aeh2779_sm.pdf]

Science Advances
AAAS

Supplementary Materials for

Geometry-controlled instability pathway selection in elastic helices enables fast, efficient robotic locomotion

Dezhong Tong *et al.*

Corresponding author: Xiaonan Huang, xiaonanh@umich.edu; M. Khalid Jawed, khalidjm@seas.ucla.edu

Sci. Adv. **12**, eaeh2779 (2026)
DOI: 10.1126/sciadv.aeh2779

The PDF file includes:

Supplementary Text
Figs. S1 to S28
Tables S1 to S6
Legends for movies S1 to S9
References

Other Supplementary Material for this manuscript includes the following:

Movies S1 to S9

Supplementary Text

The framework of the theoretical analysis of the helical buckling and the extended results are reported in section S1. The dynamic simulation is reported in section S2. The automated testbed for helical-instability experiments is described in section S3. The design, analysis, and evaluation of the snap-actuated elastic limb are discussed in section S4. The untethered hopping robot, including additional locomotion experiments, is evaluated in section S5.

S1 Theoretical analysis

To gain fundamental insight into elastic instabilities in the helical filament, we develop a theoretical framework to characterize both the onset of buckling and the post-buckling branches. This analysis reveals three key features of helical buckling: (i) the loss of stability of a helical equilibrium occurs through a pitchfork bifurcation, where a subcritical pitchfork corresponds to snap-through buckling and a supercritical pitchfork corresponds to continuous buckling; (ii) the buckling mode and the nature of the instability are determined by the loading path in the (κ, τ, ω) boundary-condition space; and (iii) along each loading ray, there is a unique critical configuration at which the helical solution loses stability, and this critical state coincides with the first conjugate point of the corresponding helical rod configuration. In the following, we first introduce our nondimensional formulation, then describe the conjugate point test used to assess stability, next trace the post-buckling branches, discuss the influence of material properties on post-buckling behavior, and present the extended theoretical results. An overview of the theory–simulation–experiment workflow is shown in Fig. S1A. Representative loading–unloading cycles further illustrate the two instability pathways that form the basis of the classification used throughout this work: snap-through to a distant non-helical configuration and continuous buckling to a nearby non-helical configuration (Fig. S1B–C).

Nondimensionalization

We model the flexible filament as an isotropic, inextensible, and unshearable Kirchhoff rod. Configurations of an elastic rod of length L are described by functions $\{\mathbf{r}, \mathbf{d}_1, \mathbf{d}_2, \mathbf{d}_3\}: [0, L] \to \mathbb{R}^3$, where $\mathbf{r}$ describes the rod centerline and $\{\mathbf{d}_1, \mathbf{d}_2, \mathbf{d}_3\}$ form an orthonormal material frame with $\mathbf{r}' = \mathbf{d}_1$, where $'$ denotes differentiation with respect to arc length s. The vector $\mathbf{u} = u_1\mathbf{d}_1 + u_2\mathbf{d}_2 + u_3\mathbf{d}_3$

satisfying $\mathbf{d}_i' = \mathbf{u} \times \mathbf{d}_i$, $i = 1, 2, 3$, is the Darboux vector for the curve $\mathbf{r}$. The bending stiffness of the rod is k_b and the torsional stiffness is k_t. The elastic potential energy of an isotropic rod configuration is

$$U = \frac{1}{2} \int_0^L \left(k_t u_1^2 + k_b u_2^2 + k_b u_3^2 \right) ds, \tag{S1}$$

where $k_t, k_b > 0$ are the torsional and bending stiffnesses. As stated in Ref. (*8*), for a rod configuration with clamped boundary conditions on $\mathbf{r}(0)$, $\mathbf{d}_i(0)$, $\mathbf{r}(L)$, and $\mathbf{d}_i(L)$ to be in equilibrium, i.e., to be a critical point of Eq. (S1), the vector of internal moments $\mathbf{m} = m_1\mathbf{d}_1 + m_2\mathbf{d}_2 + m_3\mathbf{d}_3$ and forces $\mathbf{n} = n_1\mathbf{d}_1 + n_2\mathbf{d}_2 + n_3\mathbf{d}_3$ must satisfy

$$\mathbf{m}' = \mathbf{n} \times \mathbf{d}_1, \qquad \mathbf{n}' = 0, \tag{S2}$$

and the vector $\mathbf{u}$ must satisfy $u_1 = m_1/k_t$, $u_2 = m_2/k_b$, and $u_3 = m_3/k_b$. Equilibrium configurations are found by solving the two-point boundary value problem

$$\begin{gathered} \mathbf{r}' = \mathbf{d}_1, \qquad \mathbf{d}_i' = \mathbf{u} \times \mathbf{d}_i, \qquad \mathbf{m}' = \mathbf{n} \times \mathbf{d}_1, \qquad \mathbf{n}' = 0, \\ u_1 = m_1/k_t, \qquad u_2 = m_2/k_b, \qquad u_3 = m_3/k_b, \end{gathered} \tag{S3}$$

with boundary conditions specified for $\mathbf{r}(0)$, $\mathbf{d}_i(0)$, $\mathbf{r}(L)$, and $\mathbf{d}_i(L)$. We next define nondimensional variables

$$\begin{gathered} \tilde{s} = \frac{s}{L}, \qquad \tilde{\mathbf{r}}(\tilde{s}) = \frac{\mathbf{r}(s)}{L}, \qquad \tilde{\mathbf{d}}_i(\tilde{s}) = \mathbf{d}_i(s), \\ \tilde{\mathbf{u}}(\tilde{s}) = L\mathbf{u}(s), \qquad \tilde{\mathbf{m}}(\tilde{s}) = \frac{L\mathbf{m}(s)}{k_b}, \qquad \tilde{\mathbf{n}}(\tilde{s}) = \frac{L^2\mathbf{n}(s)}{k_b}, \end{gathered} \tag{S4}$$

where $\{\tilde{\mathbf{r}}, \tilde{\mathbf{d}}_i, \tilde{\mathbf{m}}, \tilde{\mathbf{n}}\} \colon [0, 1] \to \mathbb{R}^3$. The boundary value problem in (S3), written in terms of these nondimensional variables, becomes

$$\begin{gathered} \tilde{\mathbf{r}}' = \tilde{\mathbf{d}}_1, \qquad \tilde{\mathbf{d}}_i' = \tilde{\mathbf{u}} \times \tilde{\mathbf{d}}_i, \qquad \tilde{\mathbf{m}}' = \tilde{\mathbf{n}} \times \tilde{\mathbf{d}}_1, \qquad \tilde{\mathbf{n}}' = 0, \\ \tilde{u}_1 = k\tilde{m}_1, \qquad \tilde{u}_2 = \tilde{m}_2, \qquad \tilde{u}_3 = \tilde{m}_3, \end{gathered} \tag{S5}$$

where $k = k_b/k_t$ is the ratio of bending to twisting stiffness, and boundary conditions are specified for $\tilde{\mathbf{r}}(0)$, $\tilde{\mathbf{d}}_i(0)$, $\tilde{\mathbf{r}}(1)$, and $\tilde{\mathbf{d}}_i(1)$. The corresponding nondimensional elastic energy $\tilde{U}$ is given by

$$\begin{aligned} U &= \frac{1}{2}\int_0^L \left(k_t u_1^2 + k_b u_2^2 + k_b u_3^2\right)\, ds \\ &= \frac{1}{2}\int_0^1 \left(k_t(\tilde{u}_1/L)^2 + k_b(\tilde{u}_2/L)^2 + k_b(\tilde{u}_3/L)^2\right)\, d(L\tilde{s}) \\ &= \frac{k_b}{L}\left(\frac{1}{2}\int_0^1 \left(\frac{1}{k}\tilde{u}_1^2 + \tilde{u}_2^2 + \tilde{u}_3^2\right)\, d\tilde{s}\right) \\ &= \frac{k_b}{L}\tilde{U}. \end{aligned} \tag{S6}$$

We now consider helical configurations whose centerlines have constant curvature κ and geometric torsion τ, and define the twist moment of the helical rod as ω. The corresponding nondimensional curvature, torsion, and twist moment are

$$\tilde{\kappa} = \kappa L, \qquad \tilde{\tau} = \tau L, \qquad \tilde{\omega} = \omega L. \tag{S7}$$

Solutions of Eq. (S5) that produce rod configurations with helical centerlines, i.e., centerlines with constant curvature $\tilde{\kappa}$ and torsion $\tilde{\tau}$, are given by

$$\tilde{\mathbf{u}} = \begin{bmatrix} k\tilde{\omega} \\ \tilde{\kappa}\cos(\gamma\tilde{s}) \\ \tilde{\kappa}\sin(\gamma\tilde{s}) \end{bmatrix}, \qquad \tilde{\mathbf{m}} = \begin{bmatrix} \tilde{\omega} \\ \tilde{u}_2 \\ \tilde{u}_3 \end{bmatrix}, \qquad \tilde{\mathbf{n}} = (\tilde{\omega} - \tilde{\tau}) \begin{bmatrix} \tilde{\tau} \\ \tilde{u}_2 \\ \tilde{u}_3 \end{bmatrix}, \tag{S8}$$

where $\gamma = \tilde{\tau} - \tilde{u}_1$. This nondimensionalization is equivalent to considering a rod of length 1 with effective twisting stiffness k_t/k_b and bending stiffness 1. In the manuscript and the remainder of the supplementary material, we drop the tildes over nondimensional variables for notational convenience.

Conjugate point test

For a given stationary solution of the elastic energy, the conjugate point test from the calculus of variations can be used to determine if the solution is stable, i.e., if it is a local minimum of energy among all nearby rod configurations satisfying the same boundary conditions. We apply a form of the conjugate point test described in (*35*), which involves solving the matrix differential equations

$$\mathbf{J}' = \mathbf{HJ} + \mathbf{GM} \qquad \mathbf{M}' = \mathbf{FM} \tag{S9}$$

with initial conditions $\mathbf{J}(0) = \mathbf{0}_{6\times6}$ and $\mathbf{M}(0) = \mathbf{I}_{6\times6}$. The coefficient matrices in this linear system are given by

$$\mathbf{H} = \begin{bmatrix} -\widehat{\boldsymbol{u}} & \mathbf{0}_{3\times3} \\ -\widehat{\mathbf{e}}_1 & -\widehat{\boldsymbol{u}} \end{bmatrix} = \begin{bmatrix} 0 & u_3 & -u_2 & 0 & 0 & 0 \\ -u_3 & 0 & u_1 & 0 & 0 & 0 \\ u_2 & -u_1 & 0 & 0 & 0 & 0 \\ 0 & 0 & 0 & 0 & u_3 & -u_2 \\ 0 & 0 & 1 & -u_3 & 0 & u_1 \\ 0 & -1 & 0 & u_2 & -u_1 & 0 \end{bmatrix} \tag{S10}$$

$$\mathbf{G} = \begin{bmatrix} k & 0 & 0 & 0 & 0 & 0 \\ 0 & 1 & 0 & 0 & 0 & 0 \\ 0 & 0 & 1 & 0 & 0 & 0 \\ 0 & 0 & 0 & 0 & 0 & 0 \\ 0 & 0 & 0 & 0 & 0 & 0 \\ 0 & 0 & 0 & 0 & 0 & 0 \end{bmatrix} \tag{S11}$$

$$\mathbf{F} = \begin{bmatrix} \widehat{\boldsymbol{m}} & \mathbf{0}_{3\times3} \\ \widehat{\boldsymbol{n}} & \mathbf{0}_{3\times3} \end{bmatrix} \mathbf{G} - \mathbf{H}^T = \begin{bmatrix} 0 & 0 & 0 & 0 & 0 & 0 \\ (k-1)m_3 & 0 & (k-1)m_1 & 0 & 0 & 1 \\ -(k-1)m_2 & -(k-1)m_1 & 0 & 0 & -1 & 0 \\ 0 & -n_3 & n_2 & 0 & m_3 & -m_2 \\ kn_3 & 0 & -n_1 & -m_3 & 0 & km_1 \\ -kn_2 & n_1 & 0 & m_2 & -km_1 & 0 \end{bmatrix} \tag{S12}$$

where $\mathbf{e}_1 = [1\ 0\ 0]^T$ and the map $\widehat{\cdot}$ satisfies $\widehat{a}b = a \times b$ for all $a, b \in \mathbb{R}^3$. After computing the matrix $\mathbf{J}(s)$ with $s \in [0, 1]$ (where, as described in the previous section, we are considering $L = 1$), the following conditions can be used to determine stability of the stationary solution:

- If $\det(\mathbf{J}(s)) \neq 0$ for all $s \in (0, 1]$, then the stationary solution is a local minimum of energy, and is therefore a stable equilibrium configuration.
- If $\det(\mathbf{J}(s)) = 0$ for some $s \in (0, 1)$, then the stationary solution is not a local minimum of energy, and is therefore an unstable equilibrium configuration.
- If $\det(\mathbf{J}(s)) \neq 0$ for all $s \in (0, 1)$ and $\det(\mathbf{J}(1)) = 0$, then the test is inconclusive, and the

stationary solution is on the boundary of stable and unstable equilibrium configurations.

We note that for a typical equilibrium configuration, the coefficient matrices **H**, **G**, and **F** will vary with arc length since they depend on **u**, **m**, and **n**. However, for equilibrium configurations with helical centerlines, a coordinate transformation can be applied to the linear system in (S9) so that its coefficient matrices are constant, as shown in (*36*).

Post-buckling trace

The conjugate point test from the calculus of variations, summarized in Sec. S1, can be applied to determine which helical solutions are stable, i.e., which are local minima of the elastic energy in Eq. (S1). Using this test, we establish the following properties:

1. For fixed $\kappa > 0$, τ, and ω, the stability of the corresponding helical configuration is independent of k.

2. For a fixed direction **S** defined in Eq. (1), there exists a unique $\lambda^* > 0$ such that the helical configuration with $(\kappa, \tau, \omega) = \lambda \mathbf{S}$ is stable if $\lambda < \lambda^*$ and unstable if $\lambda > \lambda^*$.

3. In particular, λ^* is the first conjugate point along the helical rod configuration corresponding to **S**, and is computed by solving the linear differential system in Eq. (S9).

By Property 1, all stable and unstable helical equilibria can be represented in the three-dimensional parameter space with axes $\kappa > 0$, τ, and ω. Properties 2 and 3 imply that, along each ray **S** in this space, there is a single loss of stability at $\lambda = \lambda^*$: as λ increases along the ray, the helical equilibrium undergoes a unique buckling transition from stable to unstable.

We now analyze the post-buckling behavior near this critical point. Starting from the helical configuration at $\lambda = \lambda^*$, we solve the equilibrium equations in Eq. (S2) with clamped boundary conditions determined by the critical helical configuration $\lambda^* \mathbf{S}$ at $s = 0$ and $s = 1$. A shooting method is used to compute solutions of Eq. (S2) that satisfy these boundary conditions. Treating the parameter λ together with the initial internal forces and moments $m_i(0)$ and $n_i(0)$ as continuation variables, we then perform pseudo-arclength continuation to trace non-helical solution branches emanating from the buckling point (*37*). The conjugate point test is applied along these branches to determine their stability.

Numerically, this stability transition corresponds to a pitchfork bifurcation of the equilibrium solutions, with two non-helical branches bifurcating from the helical branch at $\lambda = \lambda^*$. If these non-helical branches exist for $\lambda > \lambda^*$ and are stable, the pitchfork is supercritical; if instead they exist for $\lambda < \lambda^*$ and are unstable, the pitchfork is subcritical. In either case, we enforce the clamped boundary conditions for $\lambda > \lambda^*$: a supercritical bifurcation leads to continuous buckling into a nearby non-helical configuration, whereas a subcritical bifurcation results in snap-through buckling, where the rod jumps to a distant non-helical configuration, as illustrated in Fig. 2D–E.

Influence of the material properties

Although our primary interest is in how the loading directions $\mathbf{S}$ can be chosen to tune the buckling modes, the material parameter $k = k_b/k_t$ also influences which modes appear. Our theoretical framework shows that k has no effect on the critical buckling point $\lambda^*(\mathbf{S})$, but it does affect the post-buckling mode that emerges when the instability occurs, i.e., when the system transitions from a stable to an unstable helical configuration.

Under the assumption of a linearly elastic, isotropic material with a circular cross-section of radius r_0, the bending and torsional stiffnesses are

$$k_b = \frac{E\pi r_0^4}{4}, \qquad k_t = \frac{E\pi r_0^4}{4(1+\nu)}, \tag{S13}$$

where E is Young's modulus and ν is Poisson's ratio. It follows that

$$k = \frac{k_b}{k_t} = 1 + \nu. \tag{S14}$$

Since the admissible range of Poisson's ratio for isotropic linear elastic materials is $\nu \in (-1, 0.5)$, the corresponding range of k is $(0, 1.5)$.

We use our theoretical framework to systematically explore how k affects the distribution of buckling modes in helical filaments, obtaining the results shown in Fig. S2. Although varying k does modify the buckling-mode distribution, we observe pronounced qualitative changes only near $k \approx 0.33$ and $k \approx 0.67$, where the stability diagrams exhibit abrupt rearrangements that can be viewed as "snap" events in the mode distribution as material properties are varied.

For most common engineering materials, Poisson's ratio lies in the range $\nu \in [0, 0.5]$, implying $k \in [1, 1.5]$. Thus, these sharp transitions at small k fall outside the parameter range relevant for

the elastic limb used in our hopping robot and do not affect the design principles we derive here. However, composite or architected materials with negative Poisson's ratio (auxetic materials) could, in principle, be exploited to access these regimes and further tune the helical buckling behavior uncovered in this work.

Visualization of post-buckling behavior

Here, we focus on twist-free helical configurations with $\omega = 0$, which provide a canonical example of helical buckling. We set the material parameter $k = 1.33$. Following the approach described above, we derive solutions of Eq. (S2) with boundary conditions corresponding to a helical rod configuration with $(\kappa, \tau, \omega) = \lambda \mathbf{S}$ for a given unit vector $\mathbf{S}$. Fig. S3A shows 11 search directions in the $\omega = 0$ plane with κ/τ ranging from 1 to 5. The search directions with $\kappa/\tau \in \{1, 1.4, 1.8, 4.2, 4.6, 5\}$ produce snap-through buckling, while the search directions with $\kappa/\tau \in \{2.2, 2.6, 3, 3.4, 3.8\}$ produce continuous buckling.

Post-buckling behavior along search directions with $1 \leq \kappa/\tau \leq 3.39$ is shown in Fig. S3B. The horizontal axis denotes the distance λ in the search direction normalized by λ^*, so that all bifurcations occur at $\lambda/\lambda^* = 1$. The vertical axis denotes the normalized midpoint error e,

$$\mathcal{E} = \left\| \mathbf{q}_{\text{mid}} - \mathbf{q}_{\text{mid}}^{\text{helix}} \right\|, \qquad e = \frac{\kappa^2 + \tau^2}{\kappa}\, \mathcal{E}. \tag{S15}$$

Here, $\mathcal{E}$ is the Euclidean norm of the difference between the midpoint of the current rod configuration, $\mathbf{q}_{\text{mid}}$, and the midpoint of the reference helical configuration, $\mathbf{q}_{\text{mid}}^{\text{helix}}$. The scalar e is the corresponding nondimensional midpoint error, obtained by normalizing $\mathcal{E}$ using the helix radius factor $r = \kappa/(\kappa^2 + \tau^2)$.

Solid segments of the solution curves correspond to stable rod configurations, and dashed segments correspond to unstable rod configurations. Each curve in Fig. S3B represents two symmetric solution branches emanating from the bifurcation point, i.e., the two sides of a pitchfork bifurcation, both of which project onto the same curve when viewed in this two-dimensional plot. At the ends of the curves in Fig. S3B opposite to $\lambda/\lambda^* = 1$, the two symmetric solution branches converge to form a closed loop of non-helical solutions.

Near $\lambda/\lambda^* = 1$ in Fig. S3B, the non-helical solution branches for $\kappa/\tau = 1$, 1.4, and 1.8 are unstable and exist only for $\lambda/\lambda^* < 1$, thereby forming subcritical pitchfork bifurcations, consistent

with these search directions intersecting the red region of the stability boundary in Fig. S3A. The non-helical solution branches for $\kappa/\tau = 2.2$, 2.6, 3, and 3.39, on the other hand, are stable near $\lambda/\lambda^* = 1$ and exist for $\lambda/\lambda^* > 1$, thereby forming supercritical pitchfork bifurcations, consistent with these search directions intersecting the blue region of the stability boundary in Fig. S3A. After the initial supercritical buckling, these stable branches undergo a secondary fold bifurcation and become unstable, as indicated by the transition from solid to dashed lines. Although our theoretical analysis can capture this secondary fold bifurcation, it will not be further discussed and utilized in the hopping robot design. Following this instability, the two symmetric branches converge to form a closed loop, as described above.

Fig. S3C shows similar post-buckling behavior along search directions with $3.4 \leq \kappa/\tau \leq 5$, with $\kappa/\tau = 3.4$ and 3.8 buckling supercritically and $\kappa/\tau = 4.2$, 4.6, and 5 buckling subcritically, in agreement with Fig. S3A. As in Fig. S3B, each curve in Fig. S3C represents two symmetric solution branches emanating from the bifurcation point. The post-buckling behavior near $\lambda/\lambda^* = 1$ in Fig. S3B–C demonstrates the transitions from snap-through buckling to continuous buckling, and then back to snap-through buckling, that are observed in Fig. S3A as κ/τ increases from 1 to 5.

Also of interest is the behavior that occurs when symmetric solution branches meet at the ends of the curves in Fig. S3B–C opposite to $\lambda/\lambda^* = 1$. Four examples are shown in Fig. S3D. When symmetric branches converge, they are unstable and form the two sides of a subcritical pitchfork bifurcation. At this bifurcation, a stable branch exists for increasing λ/λ^*, while an unstable branch exists for decreasing λ/λ^*. For $\kappa/\tau \lesssim 3.39$, the unstable branch subsequently reverses direction and exists for increasing λ/λ^*. This fold in the solution branch does not correspond to a return to stability, but rather to the appearance of additional conjugate points. For $\kappa/\tau \gtrsim 3.4$, the unstable branch continues to exist for decreasing λ/λ^*. The shapes of these branches and their stability provide insight into the post-buckling behavior of helical rods, as indicated by red arrows in Fig. S3D.

S2 Numerical Framework for Simulation

In this section, we present the numerical framework used to formulate dynamic simulations of helical buckling. This framework allows us to quantitatively study the nonlinear behavior of the rod, compute the onset of buckling λ^* for comparison with theory, and track configuration changes before

and after instability. The simulation results guide our automated experiments that repeatedly probe helical buckling under controlled loading and unloading, including non-ideal boundary-condition changes. In addition to reproducing the intrinsic instability response under prescribed boundary loading, the same DER framework can incorporate external forces, such as contact, friction, and hydrodynamic loading, to examine how environmental interactions shape the observed motion. Together, these tools enable a closed-loop study of the mechanics of helical buckling and inform the design of the elastic limb used in our hopping robot. In what follows, we first introduce the numerical framework and then explain how boundary conditions are imposed in the simulations to probe stability numerically.

Numerical framework

The simulations used in this paper are based on the Discrete Elastic Rods (DER) formulation, which is a framework for simulating nonlinear behavior of slender elastic filaments (*30, 31*). DER has been extensively validated in prior studies across a range of filamentary phenomena, including knot tying (*38, 39*), rope deployment (*40, 41*), flagellar bundling (*42, 43*), and soft-actuator dynamics (*44, 45*). Here, we provide a brief overview of DER, and a more comprehensive exposition can be found in (*30, 31*).

A discrete representation of the rod centerline is shown in Fig. S4A. The rod is divided into $n+1$ nodes $\mathbf{q}_i \in \mathbb{R}^3$ $(0 \leq i \leq n)$ and n edges $\mathbf{e}^i = \mathbf{q}_{i+1} - \mathbf{q}_i$ $(0 \leq i \leq n-1)$. Along each edge, we attach an orthonormal reference frame $[\mathbf{d}_1^i, \bar{\mathbf{d}}_2^i, \bar{\mathbf{d}}_3^i]$ and an orthonormal material frame $[\mathbf{d}_1^i, \mathbf{d}_2^i, \mathbf{d}_3^i]$, where $\mathbf{d}_1^i$ denotes the unit tangent vector parallel to the edge direction $\mathbf{e}^i$, as shown in Fig. S4C. The reference frame is predefined at the initial simulation time $t = 0$ s and is updated in time using parallel transport (*31*), while the material frame is obtained by rotating the reference frame by a rotation angle θ^i along the shared tangent director $\mathbf{d}_1^i$. The resulting $4n+3 \times 1$ degree-of-freedom (DOF) vector is

$$\mathbf{q} = [\mathbf{q}_0, \theta^0, \mathbf{q}_1, \ldots, \theta^{n-1}, \mathbf{q}_n]. \tag{S16}$$

This DOF vector can be used to define the elastic energy of a rod configuration, which accounts for energies due to stretching, bending, and twisting. The stretching energy is

$$E_s = \sum_{i=0}^{n-1} \frac{1}{2} k_s \left(1 - \frac{\|\mathbf{e}^i\|}{\|\bar{\mathbf{e}}^i\|}\right)^2 \|\bar{\mathbf{e}}^i\|. \tag{S17}$$

In Eq. (S17), $k_s = EA$ is the axial stretching stiffness, where E is Young's modulus and A is the cross-sectional area. The length of edge $\mathbf{e}^i$ in its undeformed state is denoted by $\|\bar{\mathbf{e}}^i\|$. Moving forward, quantities denoted with a bar $\bar{}$ represent their values when the rod is undeformed.

The bending energy can be expressed as

$$E_b = \sum_{i=0}^{n-2} \frac{k_b}{\|\bar{\mathbf{e}}^i\| + \|\bar{\mathbf{e}}^{i+1}\|} \left(2\tan\frac{\phi_i}{2} - 2\tan\frac{\phi_i^0}{2} \right)^2, \tag{S18}$$

where $k_b = \frac{E\pi r_0^4}{4}$ is the bending stiffness, r_0 is the rod radius, ϕ_i is the turning angle at node i (Fig. S4C), and ϕ_i^0 is the turning angle in the undeformed state. For the naturally straight rod studied in our work, $\phi_i^0 = 0$.

Finally, the twisting energy is

$$E_t = \frac{1}{2} \sum_{i=1}^{n-1} \frac{k_t}{\|\mathbf{e}^i\|} (u_1^i)^2, \tag{S19}$$

where $k_t = \frac{E\pi r_0^4}{4(1+\nu)}$ is the twisting stiffness, ν is Poisson's ratio, $u_1^i = \theta^i - \theta^{i-1} + \Delta u_{1\mathrm{ref}}^i$ is the discrete twist at node $\mathbf{q}_i$, and $\Delta u_{1\mathrm{ref}}^i$ is the reference twist, which is determined by the twist between the reference frames along the edges $\mathbf{e}^{i-1}$ and $\mathbf{e}^i$.

Starting from an initial DOF vector $\mathbf{q}(0)$, the time-dependent DOF vector $\mathbf{q}(t)$ is computed by integrating the equations of motion at each time step using the implicit Euler method. These equations of motion are given by

$$\frac{\mathbb{M}}{\Delta t} \left(\frac{\mathbf{q}(t_{i+1}) - \mathbf{q}(t_i)}{\Delta t} - \dot{\mathbf{q}}(t_i) \right) = \mathbf{F}^{\mathrm{int}} + \mathbf{F}^{\mathrm{ext}}, \tag{S20a}$$

$$\mathbf{F}^{\mathrm{int}} = -\frac{\partial (E_s + E_b + E_t)}{\partial \mathbf{q}}, \tag{S20b}$$

$$\dot{\mathbf{q}}(t_{i+1}) = \frac{\mathbf{q}(t_{i+1}) - \mathbf{q}(t_i)}{\Delta t}, \tag{S20c}$$

where $\mathbb{M}$ is the lumped mass matrix, a diagonal matrix of size $4n + 3 \times 4n + 3$, $\mathbf{F}^{\mathrm{int}}$ denotes the internal elastic force vector and can be derived based on the gradient of the elastic energies, $\mathbf{F}^{\mathrm{ext}}$ denotes the external force vector, and $\dot{\mathbf{q}}(t_i)$ denotes the velocity vector of size $4n+3$ at time t_i. In the above time marching scheme, the previous DOF vector $\mathbf{q}(t_i)$ and the previous velocity vector $\dot{\mathbf{q}}(t_i)$ are known. By solving Eq. (S20a) with the Newton-Raphson method, we can obtain the updated degree of freedom $\mathbf{q}$ via time marching.

The external force term $\mathbf{F}^{\text{ext}}$ provides the interface through which environment–structure interactions can be incorporated into the numerical framework. In the pathway-selection analysis of this work, environmental loading is not included so that the intrinsic geometry- and boundary-loading-controlled instability pathway can be isolated. In more general environment-coupled simulations, $\mathbf{F}^{\text{ext}}$ can include frictional contact and reduced hydrodynamic loading, for example,

$$\mathbf{F}^{\text{ext}} = \mathbf{F}^{\text{contact}} + \mathbf{F}^{\text{friction}} + \mathbf{F}^{\text{hydro}}. \tag{S21}$$

For frictional contact, let $g_i(\mathbf{q}_i)$ denote the signed distance from rod node $\mathbf{q}_i$ to the object surface, with $g_i > 0$ outside the object and $g_i < 0$ under penetration. A regularized penetration depth can be written as

$$p_i = \ell_c \log\left[1 + \exp\left(-g_i/\ell_c\right)\right], \tag{S22}$$

where ℓ_c is a smoothing length. Eq. (S22) smoothly approximates $\max(-g_i, 0)$. The corresponding normal contact force can be written as

$$\mathbf{F}_i^{\text{contact}} = k_c p_i \mathbf{n}_i, \tag{S23}$$

where k_c is the contact stiffness and $\mathbf{n}_i$ is the outward surface normal. A regularized Coulomb-like friction force can then be applied along the tangential relative velocity direction:

$$\mathbf{F}_i^{\text{friction}} = -\mu \|\mathbf{F}_i^{\text{contact}}\| \Gamma(\|\mathbf{v}_i^{\text{rel,T}}\|) \frac{\mathbf{v}_i^{\text{rel,T}}}{\|\mathbf{v}_i^{\text{rel,T}}\| + \epsilon}. \tag{S24}$$

where μ is the friction coefficient, $\mathbf{v}_i^{\text{rel,T}}$ is the tangential relative velocity, ϵ is a small regularization constant, and Γ can be chosen as a smooth transition function such as

$$\Gamma(\|\mathbf{v}_i^{\text{rel,T}}\|) = \tanh\left(\frac{\|\mathbf{v}_i^{\text{rel,T}}\|}{v_{\text{tol}}}\right), \tag{S25}$$

where v_{tol} is a smoothing hyperparameter.

Hydrodynamic loading can be incorporated in the same external-force framework by applying distributed fluid forces along the rod. Let the local rod–fluid relative velocity be

$$\mathbf{v}_i^{\text{rel}} = \mathbf{v}_i - \mathbf{u}_f, \tag{S26}$$

where $\mathbf{v}_i$ is the local rod velocity and $\mathbf{u}_f$ is the local fluid velocity. Decomposing $\mathbf{v}_i^{\text{rel}}$ into tangential and normal components,

$$\begin{aligned} \mathbf{v}_i^{\parallel} &= (\mathbf{v}_i^{\text{rel}} \cdot \mathbf{t}_i)\mathbf{t}_i, \\ \mathbf{v}_i^{\perp} &= \mathbf{v}_i^{\text{rel}} - \mathbf{v}_i^{\parallel}, \end{aligned} \tag{S27}$$

a reduced-order anisotropic drag model can be written as

$$\mathbf{F}_i^{\text{hydro}} = -c^{\parallel}\mathbf{v}_i^{\parallel} - c^{\perp}\mathbf{v}_i^{\perp}, \tag{S28}$$

where $c^{\parallel}$ and $c^{\perp}$ are tangential and normal drag coefficients.

These external-force models are not used to construct the intrinsic pathway map in the main text. Instead, they indicate how the same numerical framework can be extended to environment-coupled settings after a snap-through-capable limb geometry and loading route have been selected. As a controlled example of this extension, we include a contact-constrained snap-through simulation in which blocking planes are placed along the snap-through trajectory and the resulting reaction forces are computed (section S4).

Boundary conditions

The numerical framework described in the previous section was used to simulate the behavior of elastic rods with helical centerlines. A helical rod centerline is determined by the constant values of curvature κ and torsion τ, and the twist about the centerline is determined by the constant twist moment ω. Each simulation involved selecting a unit vector $\mathbf{S}$ and then applying boundary conditions to the rod that correspond to a helix with parameters $\lambda\mathbf{S}$. When $\lambda\mathbf{S}$ denotes nondimensional boundary-condition parameters, the corresponding dimensional parameters used to prescribe the simulated rod of length L are obtained by dividing by L. The value of λ was slowly increased from zero until an instability was detected. The detection of the buckling point in the simulation and the classification of the buckling mode are detailed in the next section.

Below, we provide a brief description of the boundary conditions that were applied to the simulated rod during this process.

During the simulation, clamped boundary conditions were applied to both ends of the rod, resulting in constraints on the 14 degrees of freedom $\mathbf{q}_0$, θ^0, $\mathbf{q}_1$, $\mathbf{q}_{n-1}$, θ^{n-1}, and $\mathbf{q}_n$. The values of $\mathbf{q}_0$, θ^0, and $\mathbf{q}_1$ were held constant throughout the simulation so that the base of the rod remained fixed at the origin of the world frame, as shown in Fig. S4A. The degrees of freedom $\mathbf{q}_{n-1}$, θ^{n-1}, and $\mathbf{q}_n$ at the end of the rod were varied as the value of λ increased so that the boundary conditions applied at the rod's end corresponded to a helical configuration with parameters $\lambda\mathbf{S}$. The locations

of the nodes along this helical configuration are given by

$$\mathbf{q}_i^{\text{helix}} = \begin{bmatrix} \left(\frac{iL}{n}\right)\frac{\kappa\tau}{\kappa^2+\tau^2} - \frac{\kappa\tau}{(\kappa^2+\tau^2)^{3/2}}\sin\left(\frac{iL}{n}\sqrt{\kappa^2+\tau^2}\right) \\ \frac{\kappa}{\kappa^2+\tau^2}\left(\cos\left(\frac{iL}{n}\sqrt{\kappa^2+\tau^2}\right)-1\right) \\ \left(\frac{iL}{n}\right)\frac{\tau^2}{\kappa^2+\tau^2} + \frac{\kappa^2}{(\kappa^2+\tau^2)^{3/2}}\sin\left(\frac{iL}{n}\sqrt{\kappa^2+\tau^2}\right) \end{bmatrix}, 0 \leq i \leq n. \tag{S29}$$

During the numerical simulations, $\mathbf{q}_{n-1}$ and $\mathbf{q}_n$ were prescribed according to the above expression. Furthermore, the value of θ^{n-1} is determined by the internal twisting moment through the relationship

$$\theta^{n-1} = k\omega L = \frac{\omega L k_b}{k_t}, \tag{S30}$$

Eqs. (S29) and (S30) describe the relationship between the values of the parameters κ, τ, and ω and the boundary conditions applied at the end of the rod with respect to the world frame, as shown in Fig. S4A.

Classification of instabilities

During each simulation, the value of λ was slowly increased from zero until a bifurcation was detected, as described in the main text, and then λ was slowly returned to zero.

To determine when the discrete rod buckled, we used the midpoint position error $\mathcal{E}$ and the normalized midpoint error e defined in Eq. (S15) on the helix with parameters $\lambda\mathbf{S}$. Thresholds of $\mathcal{E}/L = 0.15$ and $e = 1.5$ were used to determine if the rod had undergone buckling from the helical configuration. Increasing λ until either $\mathcal{E}/L > 0.15$ or $e > 1.5$ defined a loading curve, and decreasing λ to 0 defined an unloading curve. This process resulted in loading and unloading curves $e = B^{\text{SIM}}_{\text{loading}}(\lambda)$ and $e = B^{\text{SIM}}_{\text{unloading}}(\lambda)$.

When the rod encounters a supercritical pitchfork bifurcation, we expect the rod to buckle continuously and reversibly, so that the loading and unloading curves are nearly identical. When the rod encounters a subcritical pitchfork bifurcation, on the other hand, we expect to see hysteresis during loading and unloading. We can therefore use the maximum vertical distance between the loading and unloading curves to distinguish between supercritical and subcritical pitchfork bifurcations. Specifically, we considered the condition

$$\max_{\lambda} |B^{\text{SIM}}_{\text{loading}}(\lambda) - B^{\text{SIM}}_{\text{unloading}}(\lambda)| < \frac{\epsilon\kappa_B}{L(\kappa_B^2+\tau_B^2)}, \tag{S31}$$

where $(\kappa_B, \tau_B, \omega_B)$ were the values of curvature, torsion, and twist moment at which buckling was detected, and $\epsilon = 0.01$. Note that the right-hand side of this inequality corresponds to ϵ times the helical centerline's radius normalized by its length at the point of instability. If this condition was satisfied, then the bifurcation was classified as supercritical, and it was otherwise classified as subcritical in numerical simulations.

Two examples of this process are shown in Fig. 2D–E. In Fig. 2D, a significant gap exists in the later portions of the loading and unloading curves, indicating a snap-through buckling (subcritical bifurcation). On the other hand, in Fig. 2E, the loading and unloading curves align, indicating a continuous buckling (supercritical bifurcation).

S3 Experimental methods

In this section, we present the automated testbed used to repeatedly probe the buckling of helical filaments, enabling us to connect theoretical and simulation-based analysis with experimentally observed buckling behavior. In what follows, we first introduce the robotic system, then describe the data collection and post-processing procedures, and finally discuss how instabilities are classified in the automated experiments.

Robotic system

Fig. S5 shows a flowchart of the automated robotic experimental stability testing process. The testing pipeline consists of three distinct components:

1. Motion planning, where the robot's joint path is computed in order to analyze buckling along directions in the κ-τ-ω parameter space.
2. Data collection, which involves the perception system for gathering experimental data.
3. Post-processing of experimental data, which is done to classify buckling types.

In the following sections, we provide additional details regarding each of these components.

Motion planning

To conduct the experiments, an industrial robot arm (Sawyer robot, Rethink Robotics) was used to manipulate a nitinol rod. One end of the nitinol rod is clamped with a static fixture, while the other end is subject to boundary conditions by the robotic arm that were obtained from the numerical simulations described in the previous section. The simulation-derived boundary conditions have two components: the centerline position at the end of the prescribed helix configuration, denoted by $\mathbf{q}_n$, and the material frame $[\mathbf{d}_1^{n-1}, \mathbf{d}_2^{n-1}, \mathbf{d}_3^{n-1}]$ which expresses the rotation at the rod's endpoint. We introduce an intermediate frame $[\mathbf{d}_1^{n-1}, \mathbf{d}_2^{n-1,\text{int}}, \mathbf{d}_3^{n-1,\text{int}}]$ shown in Fig. S4A–B, corresponding to a helical rod configuration with zero twisting moment, i.e. $\omega = 0$, that is obtained through parallel transport, which allows the intermediate frame to be moved along the centerline without twist. This process of parallel transport is described by the expressions

$$\begin{aligned}
\mathbf{b} &= \frac{\mathbf{d}_1^{i-1} \times \mathbf{d}_1^{i}}{\|\mathbf{d}_1^{i-1} \times \mathbf{d}_1^{i}\|} \text{ where } \mathbf{d}_1^{i-1} = \frac{\mathbf{e}^{i-1}}{\|\mathbf{e}^{i-1}\|}, \mathbf{d}_1^{i} = \frac{\mathbf{e}^{i}}{\|\mathbf{e}^{i}\|}, \\
\mathbf{d}_2^{i,\text{int}} &= \frac{(\mathbf{d}_2^{i-1,\text{int}} \cdot (\mathbf{d}_1^{i-1} \times \mathbf{b}))(\mathbf{d}_1^{i} \times \mathbf{b}) + (\mathbf{d}_2^{i-1,\text{int}} \cdot \mathbf{b})\mathbf{b}}{\|(\mathbf{d}_2^{i-1,\text{int}} \cdot (\mathbf{d}_1^{i-1} \times \mathbf{b}))(\mathbf{d}_1^{i} \times \mathbf{b}) + (\mathbf{d}_2^{i-1,\text{int}} \cdot \mathbf{b})\mathbf{b}\|}, \\
\mathbf{d}_3^{i,\text{int}} &= \mathbf{d}_1^{i} \times \mathbf{d}_2^{i,\text{int}},
\end{aligned} \tag{S32}$$

where $[\mathbf{d}_1^{i}, \mathbf{d}_2^{i,\text{int}}, \mathbf{d}_3^{i,\text{int}}]$ is the intermediate frame along the i-th edge with zero twist compared with the material frame $[\mathbf{d}_1^0, \mathbf{d}_2^0, \mathbf{d}_3^0]$ on the static clamped end. By rotating the endpoint intermediate frame $[\mathbf{d}_1^{n-1}, \mathbf{d}_2^{n-1,\text{int}}, \mathbf{d}_3^{n-1,\text{int}}]$ about the tangent direction by $\theta^{n-1} = k\omega = \omega k_b/k_t$ (ω is nondimensional twist moment), the endpoint material frame $[\mathbf{d}_1^{n-1}, \mathbf{d}_2^{n-1}, \mathbf{d}_3^{n-1}]$ can be obtained. The 7 DOF robot applies boundary conditions corresponding to a helix with curvature κ, torsion τ, and zero twist moment (i.e., the robot applies boundary conditions corresponding to the position q_n and orientation $[\mathbf{d}_1^{n-1}, \mathbf{d}_2^{n-1,\text{int}}, \mathbf{d}_3^{n-1,\text{int}}]$), while the external stepper motor mounted on the robot's end effector applies a rotation about $\mathbf{d}_1^{n-1}$ corresponding to a twist moment ω. This enables the robotic manipulator to apply a sequence of boundary conditions corresponding to any exploring direction $\mathbf{S}$ in the κ-τ-ω parameter space.

For a given exploring direction $\mathbf{S}$ in the κ-τ-ω parameter space, we can construct a constrained path of boundary conditions in Cartesian space to apply at the rod's endpoint. During motion planning, it is essential to ensure that the planned robot configurations are continuous and do not encounter joint singularities. Abrupt changes in the robot's configuration or joint singularities can

result in high speeds of the manipulator, which can introduce dynamic oscillations in the rod's configuration and affect stability. We implemented the Descartes planner (*33*), which is able to compute a continuous joint trajectory while avoiding such singular configurations.

The collaborative robot used in the testbed has 7 degrees of freedom, and therefore multiple joint solutions exist for a given end effector pose. To plan the joint trajectory, the Descartes planner discretizes the constrained path into a series of N poses in the robot workspace. The planner then minimizes the cost function $f = \sum_{i=1}^{N-1} \|\Theta_{i+1} - \Theta_i\|$, where Θ_i is the robot joint solution at discretization point i, which measures the total distance traveled in the robot's configuration space. The minimization of f reduces the likelihood of encountering sudden changes in the robot's joint angles due to singularities. As a result, a smooth joint trajectory corresponding to the desired boundary conditions can be generated for the robotic experiments.

Experimental Data collection and post-processing

During the experiments, an RGB-D camera (Intel RealSense D435) tracked a blue foam marker attached at the filament midpoint. The marker was segmented by color thresholding in the RGB image, yielding its pixel coordinates (u, v) and depth z_b. The 3D marker position in the camera frame, (x_b, y_b, z_b), was reconstructed using the camera intrinsics (f_x, f_y, c_x, c_y):

$$\begin{aligned} x_b &= \frac{z_b}{f_x}(u - c_x), \\ y_b &= \frac{z_b}{f_y}(v - c_y), \end{aligned} \tag{S33}$$

The detected 3D position provides the Cartesian coordinates of the midpoint marker with respect to the camera's coordinate frame. The resulting 3D midpoint positions were rigidly registered to the simulation-derived reference trajectory using the Kabsch algorithm (*46*), which computes the least-squares rotation and translation aligning the measured and simulated point sets. After registration, the measured midpoint position was compared to the corresponding helical reference configuration at the same loading parameter λ.

Experimental classification of instabilities

Compared with simulations, experimental midpoint measurements were noisier and lower-rate. We therefore classified buckling mode using a post-buckled error band and the corresponding loading

parameter λ, rather than directly comparing the maximum loading–unloading gap in e.

After detecting buckling (same criteria as in simulations), we obtained the loading and unloading curves $e = B^{\text{EXP}}_{\text{loading}}(\lambda)$ and $e = B^{\text{EXP}}_{\text{unloading}}(\lambda)$. We defined

$$e_{\max} = \min\left\{\max_{\lambda} B^{\text{EXP}}_{\text{loading}}(\lambda),\ \max_{\lambda} B^{\text{EXP}}_{\text{unloading}}(\lambda)\right\}, \qquad e_{\text{mean}} = \text{mean}\left\{B^{\text{EXP}}_{\text{loading}}, B^{\text{EXP}}_{\text{unloading}}\right\}, \tag{S34}$$

and set $\bar{e} = \frac{1}{2}(e_{\max} + e_{\text{mean}})$ with a narrow band $[\bar{e} - \Delta e, \bar{e} + \Delta e]$, where $\Delta e = 0.1\,(e_{\max} - \bar{e})$. We then collected the sets of λ samples whose measured errors fell within this band during loading and unloading.

We first applied DBSCAN clustering to the band-limited samples. If two or more clusters were returned during loading or unloading, or if no samples fell within the band (due to a rapid jump between frames), the instability was classified as subcritical (snap-through). Otherwise, we computed the mean λ values in the band,

$$\lambda_{\text{loading}} = \text{mean}\{\lambda : e \in [\bar{e}-\Delta e, \bar{e}+\Delta e]\}_{\text{loading}}, \qquad \lambda_{\text{unloading}} = \text{mean}\{\lambda : e \in [\bar{e}-\Delta e, \bar{e}+\Delta e]\}_{\text{unloading}}, \tag{S35}$$

and classified the instability as supercritical (continuous) if

$$|\lambda_{\text{loading}} - \lambda_{\text{unloading}}| < \epsilon\,\lambda_B, \qquad \lambda_B = \sqrt{\kappa_B^2 + \tau_B^2 + \omega_B^2}, \qquad \epsilon = 0.05, \tag{S36}$$

where $(\kappa_B, \tau_B, \omega_B)$ denotes the boundary-condition parameters at the detected instability. Example datasets and classifications are shown in Fig. S6.

S4 Analysis of the snap-actuated helical limb

When designing the snap-actuated helical limb, we select a critical helical configuration with zero twist moment (i.e., $\omega = 0$) to simplify both actuation and fabrication. As shown in Fig. S7, the limb consists of a single rigid holder, two connectors, and a flexible filament bent into a near-critical helix. In this section, we (i) describe the holder-design protocol, (ii) analyze limb actuation and snap-through under end rotation, (iii) compare experiments and simulations for representative candidate limb geometries, (iv) provide criteria for selecting the critical helix geometry, and (v) characterize the resulting limb through impact and blocking-force measurements, finite-speed command analysis, and repeatability tests.

Design of the rigid holder for the limb

The holder sets the boundary conditions that define the helix: it fixes the relative end-to-end displacement and the terminal tangent of the rod to match a target helical geometry parameterized by curvature κ and torsion τ (Fig. S7C). Specifically, we design the holder such that the relative end displacement $\Delta\mathbf{r}(L) = \mathbf{r}(L) - \mathbf{r}(0)$ matches that of the target helix over the rod length L.

One end of the rod is clamped in the holder via a push-fit connector, with its tangent aligned parallel to the holder surface (Fig. S7B). Given the pose of this first end and the target (κ, τ), we compute the required position $\mathbf{r}(L)$ and tangent $\mathbf{t}(L)$ at the second end, which is connected to the servo output shaft.

Without loss of generality, we define the first clamped end as $\mathbf{r}(0) = (0, 0, 0)$ and $\mathbf{t}(0) = [1, 0, 0]^T$. For a helix with $\kappa_h = \sqrt{\kappa^2 + \tau^2}$, the end position and tangent after arclength L are

$$\mathbf{r}(L) = \begin{bmatrix} \dfrac{\kappa^2 \sin(\kappa_h L)}{(\kappa^2 + \tau^2)^{3/2}} + \dfrac{L\,\tau^2}{\kappa^2 + \tau^2} \\ \dfrac{\kappa\big(\cos(\kappa_h L) - 1\big)}{\kappa^2 + \tau^2} \\ \dfrac{\kappa\tau\big(\kappa_h L - \sin(\kappa_h L)\big)}{(\kappa^2 + \tau^2)^{3/2}} \end{bmatrix}, \tag{S37}$$

and

$$\mathbf{t}(L) = \begin{bmatrix} \dfrac{\kappa^2 \cos(\kappa_h L) + \tau^2}{\kappa^2 + \tau^2} \\ -\dfrac{\kappa \sin(\kappa_h L)}{\kappa_h} \\ \dfrac{\kappa\tau\big(1 - \cos(\kappa_h L)\big)}{\kappa^2 + \tau^2} \end{bmatrix}. \tag{S38}$$

These relations directly specify the geometric constraints encoded in the holder, enabling assembly of limbs with arbitrary target (κ, τ).

Actuation of the limb

When designing the elastic limb, we leverage snap-through buckling of a twist-free helical filament (i.e., $\omega = 0$). The family of helical equilibria can be parameterized as

$$(\kappa, \tau) = \lambda(\cos\alpha, \sin\alpha), \tag{S39}$$

where κ is the curvature, τ is the torsion, α is the helix angle, and $\lambda > 0$ is a scalar loading parameter.

We exploit a full snap-through–snap-back cycle of this helical filament. As shown in Fig. S8A, during loading, the filament follows the helical equilibrium branch until it reaches the critical helical shape,

$$(\kappa^*, \tau^*) = \lambda^*(\cos\alpha, \sin\alpha), \tag{S40}$$

at which point the helical configuration loses stability and snaps through to a non-helical shape. Upon reversing the loading, the boundary conditions are driven back along the non-helical branch until the system reaches a second instability and snaps back to a reset helical shape,

$$(\kappa^{\downarrow}, \tau^{\downarrow}) = \lambda^{\downarrow}(\cos\alpha, \sin\alpha). \tag{S41}$$

In our theoretical analysis, the ideal loading path is obtained by tracking the helical equilibria as λ increases from $\lambda^{\downarrow}$ to λ^*. This path defines a coupled trajectory for both the position and rotation of the actuated end, as illustrated in Fig. S8B.

We now use this ideal loading path to design an actuation strategy that uses only a single degree of freedom. In the main text, we restrict attention to critical helical shapes with helix angles $\alpha \in (0, 0.24)$ when selecting limb candidates. For these configurations, Fig. S8C shows the normalized displacement change $\Delta r/L$ of the actuated end along the ideal loading path. The displacement variation is modest (typically below 16%), indicating that the snap-through cycle is dominated by rotation rather than translation. Motivated by this observation, we neglect the displacement change in our actuation scheme.

Next, we analyze the rotation along the loading path. We introduce two orthonormal axes in the rotation plane. The principal rotation axis $\mathbf{e}_1$ is chosen such that the rotation about $\mathbf{e}_1$ has the largest amplitude along the loading path, while the secondary axis $\mathbf{e}_2$ is orthogonal to $\mathbf{e}_1$ and captures the remaining, smaller rotation component. We denote the corresponding rotation angles by θ_1 and θ_2, respectively. As shown in Fig. S8D, the secondary angle θ_2 remains much smaller than the primary angle θ_1 over the entire loading path.

These results suggest a natural simplification: instead of following the full ideal loading path, we actuate the limb by rotating the tangent at the actuated end of the critical helical shape about the principal axis $\mathbf{e}_1$ to trigger the snap-through–snap-back cycle. In other words, we replace a coupled position–rotation loading protocol with an effectively single-axis rotational actuation.

Fig. S8E–F compare the normalized system energy over a full snap-through cycle under the

ideal loading path and under this simplified actuation for two limb candidates, $\alpha = 0.11$ and $\alpha = 0.20$. The loading cycles exhibit good agreement, and the energy release is nearly identical. Thus, the simplified actuation preserves the essential energetics of the ideal loading, while being much easier to realize mechanically. We therefore adopt this design principle to implement the actuation mechanism for helical buckling in the elastic limb.

Experiment–simulation comparison for candidate limb geometries

Before selecting the final operating geometry, we fabricated and tested representative snap-actuated limbs with critical helix angles $\alpha = 0.01$, $\alpha = 0.11$, and $\alpha = 0.20$. The experiment–simulation comparison for the selected $\alpha = 0.11$ limb is shown in Fig. 3C–E in the main text. Additional snapshots comparing experiments and dynamic simulations for the $\alpha = 0.01$ and $\alpha = 0.20$ limbs are provided in Fig. S9. These comparisons confirm that the dynamic simulations capture the observed snap-through reconfiguration across the candidate limb geometries used in the subsequent selection analysis.

Selection of the critical helical configuration

Since many critical helical configurations can, in principle, be used to design a powerful elastic limb for hopping, we first establish a rule for selecting the most suitable one for our task. A schematic of a single hop is shown in Fig. S10A. We identify two key requirements for a strong, effective hop: (i) the robot should achieve a substantial horizontal jump distance Δx with a finite hop height h, so that it can move quickly while retaining the ability to clear small obstacles; (ii) the robot should be able to reset its limb in the ballistic flight phase, thereby minimizing ground contact time and mitigating the adverse effects typically associated with resetting snap-through structures (Fig. S10A). These hopping requirements translate into two design criteria for the elastic limb: (i) the energy released during snap-through, ΔU, should be large; (ii) the critical actuation angle $\Delta\theta$ should not be too large (Fig. S10B).

We have previously carried out a static theoretical analysis of all twist-free helical buckling modes, as summarized in Fig. 3B. However, that analysis is purely static and is based on an idealized loading path. In our robot, the actuation strategy is simplified as described above, so we perform dynamic simulations to compute the energy release ΔU (normalized according to Eq. (S6)) and the

critical actuation angle $\Delta\theta$ for different critical helical shapes. As discussed in the main text, we restrict attention to critical helical shapes with helix angle $\alpha \in (0, 0.24)$. The results are shown in Fig. S10C–E.

Overall, as the helix angle α increases, the helical buckling releases more energy, in agreement with the static theoretical predictions. Interestingly, the simulations also reveal a decrease in energy release for very small helix angles $\alpha \in (0, 0.02)$, in contrast to the static analysis. This discrepancy arises from the delay in snap-through when the helical filament is actuated at relatively high speed, a dynamic effect that has been discussed in previous studies (*1, 47, 12, 11*). Another key observation is that the critical actuation angle $\Delta\theta$ increases monotonically with α. In other words, larger energy release ΔU in helical buckling is typically accompanied by a larger critical actuation (reset) angle, and hence longer reset times.

To balance these competing trends, we introduce an angular actuation efficiency

$$\eta_\theta(\alpha) = \frac{\Delta U}{\Delta\theta}, \tag{S42}$$

which quantifies the energy released per unit actuation angle. We find that this efficiency is maximized at a helix angle $\alpha^* \approx 0.11$, and we therefore select this critical helical shape to build the elastic limb used in the hopping robot. We set L and r_0 from direct measurements of the nitinol filament and used Poisson's ratio $\nu = 0.33$. Because Young's modulus E is difficult to measure reliably for our thin superelastic filament ($r_0 = 0.4$ mm), we used an effective modulus calibrated to experiments by matching the simulated midpoint-speed profiles during snap-through for multiple helical limbs, and then held this E fixed across all simulations in this section. The calibrated modulus was $E = 33$ GPa, which is consistent with reported values for superelastic nitinol rods (*48*).

Finite-speed actuation, command design, and repeatability of snap-through/reset

The pathway-selection map in the main text is constructed under quasi-static boundary loading and identifies the geometry/loading regimes that select snap-through or continuous buckling. This pathway-selection framework also motivates a simple boundary-command strategy: along the stable branches, the limb configuration is governed by the imposed boundary rotation, and the rapid transition is triggered once the actuated boundary crosses the snap-through or reset threshold. In the physical limb, boundary rotation is imposed by a servo motor at finite speed (Fig. S11A). We

therefore performed limb-level simulations and experiments to determine how the selected snap-through pathway is dynamically realized under finite-speed actuation and how the discontinuous snap-through/reset transition can be commanded repeatably without high-bandwidth tracking of the rapid transition.

The finite-speed simulations used the same limb geometry and calibrated material parameters described in Materials and Methods. From the quasi-static response of the selected limb, we identify the snap-through threshold, θ_{snap}, and the reset threshold, θ_{reset} (Fig. S11B). In the implemented command, the servo is not commanded to stop exactly at these marginal thresholds. Instead, the target angles are chosen to lie beyond the thresholds:

$$\theta_{\text{snap,tar}} = \theta_{\text{snap}} + \delta\theta, \qquad \theta_{\text{reset,tar}} = \theta_{\text{reset}} - \delta\theta, \tag{S43}$$

The offset $\delta\theta$ between each target angle and its corresponding threshold serves as a command margin. This margin is an engineering choice whose role is to ensure that the actuated boundary crosses the snap-through and reset thresholds reliably despite finite motor speed, motor tolerance, and uncertainty in the threshold locations. Increasing the command margin generally reduces the post-target delay and therefore improves the robustness of snap-through/reset.

To define the transition timing, we track the limb midpoint and introduce a normalized transition coordinate:

$$\eta(t) = \frac{x_{\text{mid}}(t) - x_{\text{mid},A}}{x_{\text{mid},B} - x_{\text{mid},A}}, \tag{S44}$$

where $x_{\text{mid},A}$ and $x_{\text{mid},B}$ denote representative midpoint positions on the initial helical branch and the post-snap branch, respectively. The transition onset is defined as the first time $t_{\eta=0.1}$ at which $\eta(t) \geq 0.1$, and the transition completion is defined as the first post-snap peak t_{peak} satisfying $\eta(t_{\text{peak}}) \geq 0.9$. The snap-through duration is then

$$T_{\text{snap}} = t_{\text{peak}} - t_{\eta=0.1}. \tag{S45}$$

For command design, the more relevant quantity is the post-target delay. We define:

$$T_{\text{delay}} = \max\left(0, t_{\text{peak}} - t_{\text{tar}}\right), \tag{S46}$$

where t_{tar} is the first time at which the actuated end reaches the prescribed snap-through target angle $\theta_{\text{snap,tar}}$. If snap-through completes before or at the instant when the target angle is reached, the

delay is set to zero. Thus, T_{snap} characterizes the intrinsic fast transition, whereas T_{delay} determines the waiting time required after the target angle is reached before commanding reset (Fig. S11C).

Dynamic simulations were used to examine how T_{snap} and T_{delay} depend on actuation speed and command margin (Fig. S11D–E). Across the tested command conditions, T_{snap} remained bounded and comparable to the elastic-inertial time scale of the limb:

$$T_0 \sim \sqrt{\frac{\rho A L^4}{EI}}, \tag{S47}$$

where ρ is the material density, A is the cross-sectional area, L is the limb length, E is Young's modulus, and I is the second moment of area. This result indicates that the rapid transition is governed primarily by the limb material and geometry rather than by direct motor tracking. The computed post-target delay T_{delay} decreases when the commanded target is moved farther beyond the quasi-static instability threshold. It remains below the dwell period selected for the implemented command.

The implemented command cycle consists of actuation, dwell, reset, and dwell, as illustrated by the idealized command sequence in Fig. S11F:

$$T_{\text{cycle}} = T_{\text{act}} + T_{\text{dwell}} + T_{\text{reset}} + T_{\text{dwell}}, \quad f_{\text{act}} = 1/T_{\text{cycle}} \tag{S48}$$

Here, T_{act} is the loading interval before snap-through, T_{reset} is the reset interval, and T_{dwell} is the prescribed dwell period inserted after both the snap-through and reset commands. The simulation-based timing analysis shows that both T_{snap} and T_{delay} are bounded and remain on the order of the elastic-inertial time scale T_0, which is consistent with Ref. (*1*). We therefore used a conservative dwell-time rule, $T_{\text{dwell}} > 2T_0$, so that the dwell interval exceeds the characteristic snap-through/delay time scale before reset is commanded. For the selected limb, $T_0 = 0.04$ s, and we used $T_{\text{dwell}} = 0.1$ s in all robotic experiments. This dwell time also exceeds the computed post-target delay over the tested command conditions. Thus, the controller does not need to detect or track the exact snap-through instant in real time: it drives the boundary beyond the target angle, holds for a conservative dwell interval, and then reverses the boundary motion for reset (Fig. S11F).

We next performed limb-level finite-speed experiments and simulations over a range of measured actuation speeds. In the experiments, the servo was driven by discrete target-position commands with prescribed timing (Fig. S12A). Because the realized servo motion depends on load and the internal

servo controller, we report the measured actuation speed extracted from the recorded boundary motion rather than the nominal command speed. For each finite-speed experiment–simulation comparison, the command margin was set to match the corresponding experimental protocol, so that simulation and experiment used the same prescribed boundary path and the effect of actuation speed could be isolated.

Across all tested actuation speeds, snap-through occurred successfully. Increasing actuation speed increased the peak midpoint velocity and modified the post-snap oscillatory response, but did not suppress the selected snap-through pathway. The experimental and simulated midpoint-velocity trajectories agree well in the snap-through window (Fig. S12B–F), and the peak midpoint velocity approaches a bounded range over the tested speeds (Fig. S12G). When time is shifted by the first velocity peak and velocity is normalized by the peak value, the pre-peak growth profiles show comparable rapid acceleration before snap-through completion (Fig. S12H). These results show that the quasi-static pathway map predicts which instability pathway is selected, while the finite-speed actuator-level analysis characterizes how that pathway is dynamically realized under motor actuation.

For robot locomotion, we used the same conservative command principle with a prescribed servo command range chosen to robustly cross the snap-through and reset thresholds during repeated operation. This conservative command is not required to identify the snap-through threshold itself; rather, it provides an engineering margin for reliable cyclic locomotion. The same prescribed servo command sequence and timing were used for the rigid-legged control, providing a fair comparison in which both robots were driven by the same finite motor swing.

Finally, we quantified repeatability using a long-duration limb-level actuation test. A successful cycle was defined as completion of both the snap-through transition and the motor-driven reset to the initial helical configuration. Across 1200 consecutive cycles, we observed 1200 successful snap-through/reset cycles and no failed resets, corresponding to a 0% observed failure rate under the tested command protocol. The corresponding representative video is provided as Movie S3. Together, these results show that the snap-through event is discontinuous but mechanically programmed and repeatable: the low-level controller imposes boundary loading, dwell, and reset, while the rapid transition is produced by the elastic limb mechanics.

Characterization of the elastic limb

To further evaluate the performance of the elastic limb designed from the critical helical configuration with $\alpha = 0.11$, we conduct impact tests using a hanging lightweight ping-pong ball. As shown in Fig. S13A, a lightweight ping-pong ball with measured mass of 1 g is suspended from a string. We then actuated the limb to "kick" the ball and compared the transmitted motion with that generated by a rigid limb under the same command. Using the motion tracking experimental setup, we extract the displacement–time trajectories of the ball after impact. Fig. S13B compares the resulting motion for the snap-actuated helical limb and a rigid limb. After impact, the ball reaches an initial speed of approximately 5.85 m/s with the snap-actuated helical limb, but only 1.29 m/s with the rigid limb. Thus, the snap–actuated limb releases and transmits substantially more energy than the rigid leg under the same actuation conditions. These impact experiments qualitatively demonstrate the large impulse that the elastic limb can impart to its environment, relative to a rigid counterpart.

Second, we characterized the blocking force generated by the elastic limb, defined as the maximum force it can exert when its tip motion is constrained. We utilized the force measurement experimental setup shown in Fig. S14A. The limb holder is mounted on the bottom clamp of a tensile testing machine (Instron 68SC-5) at a prescribed angle relative to the horizontal, using a custom 3D-printed spline-like radial slot (White resin, Formlabs) that allows precise angle adjustment. A rigid 3D-printed plate (PC, Bambu Lab), mounted on the top clamp in series with a load cell, is used to constrain the limb tip. The plate displacement d is defined as the downward displacement of the top clamp. When $d = 0$, the limb remains fully unconstrained and does not contact the plate during actuation. As d increases, the plate progressively constrains the limb, and when $d = d_{\max}$ the limb is fully constrained and can no longer reconfigure during actuation. The actuation voltage is 7.4 V, and the actuation time is set to be identical to that used in the untethered robot tests, enabling direct comparison between the two experiments. A translation stage is mounted on the bottom clamp to ensure precise alignment between the limb and the top plate.

When scanning the entire range of plate displacements d, we observe a non-monotonic (inverted U-shaped) dependence of the peak blocking force on d: the force first increases and then decreases. For relatively small d, the limb can snap through from the critical helical state to a non-helical state.

As d increases, more of the released elastic energy is redirected into overcoming the constraint, thereby generating larger forces. The peak blocking force is typically observed around $d/d_{\max} \approx 0.24$. For larger d, the elastic limb is sufficiently constrained that it can no longer snap through to a non-helical state; consequently, the peak blocking force decreases with further increases in d.

We measure a maximum peak force of $\approx 3.3\,\mathrm{N}$ for a single limb mounted at $0°$ when $d/d_{\max} \approx 0.24$. For a mounting angle of $15°$, the maximum peak force is slightly lower, $\approx 3.2\,\mathrm{N}$, and is also attained near $d/d_{\max} \approx 0.24$. These measurements show that the limb generates comparable peak blocking forces over the mounting-angle range used in the robot design, which we further refine through iterative design and fabrication.

Contact-constrained snap-through and blocking-force simulation

To provide a controlled example of environment–structure coupling and to examine the burst-like forces generated by the snap-actuated limb, we performed a contact-constrained snap-through simulation using the selected helical limb. The goal of this test is not to construct a complete model of terrain or fluid interaction, but to illustrate how external contact constraints can be incorporated into the rod-based simulation framework through the external force term $\mathbf{F}^{\text{ext}}$ and how the resulting environment-side reaction forces can be quantified.

We first computed the unconstrained snap-through trajectory of the selected limb under the prescribed boundary rotation. We then placed rigid blocking planes at different locations along the midpoint trajectory (Fig. S15A). For each blocking location, the plane normal was aligned with the local tangent direction of the unconstrained midpoint trajectory, so that the plane locally opposed the dominant snap-through motion. When the snapping limb contacted the blocking plane, the simulation computed the resulting contact reaction force. This setup provides a simple contact-constrained model problem for characterizing the burst-like environment-side output generated by the selected snap-through pathway. All blocking-force simulations used the selected limb geometry and the same calibrated material parameters, discretization, damping, time-integration scheme, contact stiffness, and blocking-plane size across all blocking locations.

For each blocking location, we recorded both the actuated-end input torque and the reaction force generated on the blocking plane. The blocking location was parameterized by the normalized arc length $\bar{s}$ along the unconstrained midpoint trajectory. To reduce sensitivity to high-frequency

numerical contact oscillations, the contact-force magnitude was smoothed using a 1-ms moving-average window, and the peak of the smoothed signal was reported as the peak blocking force $F_{\text{block,max}}$. The peak blocking force increases along the trajectory and reaches approximately 3.35 N over part of the snap-through pathway (Fig. S15B).

We also computed an effective output moment to characterize the environment-side moment generated by constrained snap-through. The base reference point $\mathbf{q}_{\text{base}}$ was defined as the midpoint between the actuated-end reference point and the clamped-end center, as illustrated in Fig. S15A. Because the dominant midpoint trajectory lies primarily in the x–z plane, the effective output moment was projected along the corresponding y-axis:

$$\tau_{\text{out}} = \mathbf{e}_y \cdot [(\mathbf{q}_{\text{block}} - \mathbf{q}_{\text{base}}) \times \mathbf{F}_{\text{block}}] , \tag{S49}$$

where $\mathbf{q}_{\text{block}}$ is the blocking-contact location and $\mathbf{F}_{\text{block}}$ is the reaction force on the blocking plane. The effective output moment reaches approximately 0.12 $\text{N} \cdot \text{m}$ along part of the constrained snap-through pathway (Fig. S15C). We further computed the effective output/input torque ratio as $R_\tau = \tau_{\text{out}}/\tau_{\text{in}}$, where τ_{in} is the actuated-end input torque measured in the same simulation. The effective output/input torque ratio reaches values around 3.30 along part of the pathway (Fig. S15D).

These contact-constrained simulations show that the selected snap-through pathway can generate substantial environment-side reaction forces when its motion is locally blocked. The results demonstrate that the same rod-based simulation framework used for boundary-driven snap-through can be extended to contact-constrained interactions and can quantify reaction forces generated during constrained snap-through. These protocol-specific measurements complement the free-snap-through pathway analysis by quantifying how the selected limb transfers stored elastic energy into environment-side reaction forces when constrained by contact.

S5 Additional tests and analysis of the untethered robot

In this section, we report additional results and analyses for the untethered hopping robot. We first summarize the robot and rigid-legged control designs and the shared power/control architecture used for controlled comparisons. We then analyze actuation-cycle timing, additional locomotion modes, aquatic tunability, terrain-dependent hopping performance, electrical cost of transport, robot-level reaction forces, and robot–substrate friction. Finally, we describe the teleoperation framework used

in unstructured environments, the light-guided feedback demonstration using onboard sensing, and the comparison with animals and previously reported untethered terrestrial robots.

The snap-actuated robot and the rigid-legged control are shown in Fig. S16. Both platforms use the same body architecture, onboard battery, control board, micro-servos, holder inclination, effective limb length, contact-pad material, and substrate set; the controlled design variables are summarized in Table S1. The shared power and control architecture is shown in Fig. S17. This architecture sends the same prescribed servo command sequence to the snap-actuated and rigid-legged robots, ensuring that the controlled comparison isolates the effect of replacing the snap-actuated helical limbs with rigid counterparts.

Actuation-cycle frequency dependence of robot hopping performance

To quantify how command timing affects robot-level locomotion, we performed an actuation-cycle frequency sweep using the snap-actuated hopping robot. Unlike a sinusoidal gait, the robot is driven by a discrete snap-through/reset command. One actuation cycle consists of four phases: loading, dwell, reset, and dwell. The command-cycle duration is therefore as defined in Eq. (S48) and the actuation frequency is defined as $f_{\text{act}} = 1/T_{\text{cycle}}$. In this sweep, we fixed $T_{\text{dwell}} = 0.1s$, following the limb-level timing analysis, and used the fastest reliable reset command, giving $T_{\text{reset}} \approx 0.13s$. We varied T_{act} by changing the timing of the servo target-position commands. This changes the actuation speed before snap-through and therefore changes f_{act}.

The sweep was performed over $f_{\text{act}} = 0.47 - 2.13$ Hz. For each frequency, we measured the maximum normalized body height and the normalized forward speed from side-view videos. The maximum body height was normalized by the robot body height, and the forward speed was normalized by body length. Repeated trials were used to compute the mean and standard deviation (Fig. S18A–B).

The results show that robot-level hopping performance depends strongly on actuation-cycle frequency. At low actuation-cycle frequencies, the limb can still complete the snap-through/reset command, but the robot remains in contact with the substrate for much of the cycle. In this regime, the body height remains low, and the robot exhibits a crawling-like contact motion rather than sustained hopping. This behavior is consistent with the limb-level finite-speed analysis: lower actuation speed is expected to reduce the burst-like character of the snap-through impulse, while

the longer loading interval also gives the body more time to settle or pitch before the transition is triggered.

As the actuation-cycle frequency increases, the robot transitions from the crawling-like contact regime to marginal hopping and then to stable hopping. The measurements reveal an empirical transition in body height: when the maximum normalized body height is below approximately 0.54 body heights, the robot does not maintain a sufficient flight phase for robust reset before the next ground contact. Near $f_{\text{act}} \approx 1.34$ Hz, the robot enters a marginal hopping regime, where short flight phases begin to appear but the motion remains less repeatable. At higher actuation-cycle frequencies, the body height increases beyond this empirical transition, reset can occur during the airborne phase, and the robot enters a stable hopping regime with higher and more repeatable forward speed.

These results show that actuation-cycle frequency affects robot performance through both actuator output and gait coordination. At the limb level, snap-through and reset can be completed over the tested frequency range. At the robot level, however, stable hopping requires a sufficiently strong burst-like impulse and a flight phase long enough for the limb to reset before the next ground contact (Fig. S18C).

Untethered robot turning and swimming

We evaluated the capabilities of the untethered hopping robot in two additional motion modes: turning and swimming. Benefiting from the powerful elastic limbs enabled by helical snap-through, actuating only a single limb generates an agile turning gait. Snapshots of the robot turning and example time traces of its orientation are shown in Fig. S19A–B. Using the robot's center as the tracked point, we find that the turning radius is $\approx$ 3.22cm (0.29 BL), indicating high maneuverability. The example time traces further show that the robot's instantaneous turning speed reaches $151^\circ/\text{s}$ (Fig. S19B). A comprehensive set of experiments reveals that the robot achieves an average turning speed of $163 \pm 12.5^\circ/\text{s}$, demonstrating fast and repeatable turning performance.

Leveraging the waterproof design shown in Fig. S20, the same platform is also capable of amphibious swimming. Snapshots of the robot hopping into a water tank and swimming are presented in Fig. S19C, and the tracked trajectory of its center is shown in Fig. S19D. Driven by its elastic limbs, the robot paddles through the water in a manner reminiscent of a duck using its feet.

Under this gait, the robot reaches a swimming speed of approximately 0.5 BL/s.

Exploratory aquatic-locomotion study: effects of actuation-cycle frequency and limb installation angle

The swimming demonstration in the main text used the same limb installation angle as the terrestrial hopping robot. This hopping configuration was chosen to demonstrate that the snap-through limb can generate propulsion in both terrestrial and aquatic environments, but it was not optimized for swimming. To explore how aquatic performance depends on command timing and limb reconfiguration, we performed two exploratory aquatic-locomotion studies.

First, we varied the actuation-cycle frequency while keeping the hopping-robot limb configuration fixed. The actuation-cycle frequency f_{act} follows the same command-cycle definition introduced in Eq. (S48). In the aquatic frequency sweep, the snap-through stroke was treated as the power stroke, while the reset stroke was kept slower and fixed as a recovery stroke to reduce adverse reverse thrust. Specifically, we varied the snap-through power-stroke duration T_{act} while keeping $T_{reset} = 0.8$ s and $T_{dwell} = 0.1$ s fixed. Therefore, changing T_{act} changed both the actuation speed of the snap-through power stroke and the actuation-cycle frequency f_{act}, without changing the robot body, limb installation angle, or actuation hardware.

The normalized swimming speed increased over the tested range, from approximately 0.14 body lengths per second at $f_{act} = 0.65$ Hz to approximately 0.49 body lengths per second at $f_{act} = 0.88$ Hz (Fig. S21A and C). This result shows that aquatic performance is tunable through command timing. The lower swimming speed at lower actuation-cycle frequency is expected because fewer snap-through power strokes are generated per unit time, and the slower power stroke also reduces the thrust generated in each cycle.

Second, we varied the limb installation angle at fixed actuation-cycle frequency $f_{act} = 0.88$ Hz. The limb installation angle, denoted by ϕ, is defined as the angle between the motor base and the horizontal direction, with counterclockwise rotation taken as positive. The swimming speed depended strongly on ϕ (Fig. S21B and D). The hopping configuration, corresponding to the limb installation angle used for terrestrial hopping, enabled swimming but was not the highest-speed configuration under the tested conditions. A more swimming-favorable installation angle produced higher swimming speed, indicating that limb installation geometry provides an additional design

parameter for aquatic propulsion.

These results are exploratory and are not intended to identify a globally optimized swimming design. Instead, they show that aquatic locomotion performance is tunable through both actuation-cycle frequency and limb installation angle. Swimming-specific co-design of snap-through geometry, limb installation, body morphology, and fluid–structure interaction remains an important direction for future work.

Untethered robot back-flipping

By adjusting the design of the front-balanced limbs to shift the center of mass, our untethered robot can also perform consecutive back-flips. The updated limb design parameters are given in Fig. S22. Two main modifications are introduced: (i) the limb height is slightly reduced, which changes the contact angle with the ground, and (ii) the limb is shortened in the horizontal direction, shifting the robot's center of mass toward the elastic limbs that provide the snap-through power. These adjustments increase the contribution of the elastic limbs to pitching motion and enable the robot to execute robust, consecutive back-flips, a behavior that requires large, rapid body reorientation. Fig. S22B–I illustrate one back-flip cycle, during which the robot performs a bicycle kick to strike a hanging ping-pong ball.

Untethered robot hopping across different terrains

We evaluated the locomotion performance of the snap-actuated robot and the rigid-legged control on a range of terrains. Example time traces of the tracked robot positions are shown in Fig. S23. The snap-actuated robot exhibits a repeatable hopping gait and traverses all tested terrains with stable forward progression. It achieves its highest speed on the wooden surface and its lowest speed on grass.

The measured terrain-dependent speeds reflect both effective robot–substrate friction and substrate mechanical response, including compliance, slipping, and local deformation. The effective robot–substrate friction conditions are characterized below in the robot-level friction measurements. On wood, the snap-actuated robot achieves its maximum speed of 35.31 ± 1.04 cm/s (3.21 ± 0.10 BL/s), whereas the rigid-legged control reaches 13.2 ± 0.43 cm/s (1.20 ± 0.04 BL/s). Acrylic and leather have similar friction coefficients, and the rigid-legged control attains compa-

rable speeds on these two terrains, 12.18 ± 1.84 cm/s (1.11 ± 0.21 BL/s) and 11.91 ± 2.98 cm/s (1.08±0.27 BL/s), respectively. In contrast, the snap-actuated robot moves slightly slower on leather, 22.96 ± 1.15 cm/s (2.09 ± 0.10 BL/s), than on acrylic, 27.33 ± 1.11 cm/s (2.48 ± 0.02 BL/s), consistent with additional energy dissipation in the softer leather substrate.

A particularly striking case is the cloth surface. The snap-actuated robot moves rapidly on cloth, 27.51 ± 0.18 cm/s (2.50 ± 0.02 BL/s), whereas the rigid-legged control nearly stalls, reaching only 1.06 ± 1.84 cm/s (0.10 ± 0.17 BL/s). This difference is consistent with the cloth layer sliding relative to the rigid substrate underneath. Under the sustained tangential loading produced by the rigid-legged gait, the cloth–substrate interface can shear and slip, so much of the leg motion is absorbed by cloth motion rather than producing forward body translation. By contrast, the hopping gait enabled by the snap-through kick reduces the duration of surface contact and mitigates slippage, allowing the snap-actuated robot to maintain high speed.

Another challenging case is grass. The rigid-legged control again barely moves, with a speed of 2.44 ± 0.45 cm/s (0.22 ± 0.04 BL/s), because grass forms a deformable and geometrically irregular terrain with local height variations that hinder locomotion. The snap-actuated robot also attains its slowest speed on grass, 21.00 ± 0.65 cm/s (1.91 ± 0.06 BL/s). Nonetheless, even on this challenging terrain, its speed remains approximately 60% of its maximum value on wood. This comparison shows that the snap-through-enabled hopping gait improves terrain robustness relative to the rigid-legged control by combining rapid impulse generation with reduced sustained ground contact.

Minimizing the cost of transport of the untethered robot

We calculate the total cost of transport CoT of the untethered robot as

$$CoT = \frac{E_{\text{in}}}{mgd}, \tag{S50}$$

where E_{in} is the electrical input energy from the LiPo battery, m is the robot mass, and d is the distance traveled using that input energy. Since $E_{\text{in}} = P_{\text{in}}T$ and $d = vT$, where P_{in} is the input power, v is the average speed, and T is the duration of the experiment, Eq. (S50) can be rewritten as

$$CoT = \frac{P_{\text{in}}}{mgv}. \tag{S51}$$

To evaluate P_{in}, we measure the electrical power drawn from the LiPo battery that drives the servo motors. The servos control the actuation angle at the clamped ends of the nitinol limbs, causing them to switch between helical and non-helical configurations. During snap-through, each limb releases stored elastic energy and delivers an impulsive force against the ground, propelling the robot forward. During the flight phase, the motors reverse the actuation angle to reset the limb to its helical configuration in preparation for the next hop. In our experiments, the actuation frequency is approximately 2 Hz. To measure the input power, we fix the input voltage at 7.4 V using a regulated power supply and measure the total system current in real time. The electrical schematic of the system is shown in Fig. S24E. The system current is measured using a current sensor (INA219, Adafruit), and the data are transmitted to a microcontroller (Arduino Uno, Arduino) at a sampling rate of 100 Hz through the I2C bus. We test both the hopping robot and the rigid-legged robot under the same experimental conditions as in the locomotion tests (Fig. 5). The corresponding current time traces are shown in Fig. S24A–D.

From these measurements, we obtain average currents of 0.22 A for the untethered hopping robot and 0.19 A for the rigid-legged robot, corresponding to average electrical powers of 1.63 W and 1.41 W, respectively. Thus, the elastic-limb robot draws only slightly more electrical power (a $\sim 16\%$ increase), which is expected since its compliant limb undergoes large cyclic deformations to store and rapidly release elastic energy, requiring additional work from the actuators and incurring internal dissipation. As shown below, this modest increase in power is more than compensated by the substantially higher locomotion speed, resulting in a lower cost of transport compared with the rigid-legged robot.

The robot's mass is $m = 98.2$ g. Using Eq. (S51) and the measured speeds on each terrain, we compute the cost of transport for both robots across all tested terrains. The full set of CoT values is listed in Table S3. The minimum CoT of the untethered hopping robot is $CoT = 4.79$ on wood, whereas the minimum CoT of the rigid-legged robot is $CoT = 11.76$, also on wood. On more challenging terrains, the rigid-legged robot becomes highly inefficient, with CoT values of approximately 141.11 and 64.14 on cloth and grass, respectively. By contrast, our untethered hopping robot maintains $CoT < 10$ on all tested terrains. These results demonstrate that the snap-through-enabled hopping gait not only enhances speed and terrain robustness, but also significantly improves energetic efficiency relative to a rigid-legged design.

Robot-level reaction-force and substrate-friction characterization

To complement the kinematic and cost-of-transport measurements, we characterized the robot-level vertical reaction force during hopping and the effective robot–substrate friction conditions used in the locomotion experiments.

For the reaction-force measurement, the robot was operated on an acrylic substrate mounted on a load cell (Fig. S25A). We measured the raw vertical reaction force $F_{\text{react}}(t)$ for both the snap-actuated robot and the rigid-legged control under matched hardware and command conditions. The raw force includes the initial static robot weight and preload before hopping. To quantify the impulsive force transmission during hopping, we first subtracted the baseline force F_{base} measured before the hopping phase. The excess vertical impulse was then computed as

$$J_z = \int_{t_1}^{t_2} \left[F_{\text{react}}(t) - F_{\text{base}}\right]\, dt, \tag{S52}$$

where t_1 and t_2 denote the onset of the force rise and the time when the baseline-subtracted force returned to zero, respectively.

The snap-actuated robot generated a larger and more rapid increase in vertical reaction force than the rigid-legged control (Fig. S25C). The peak raw vertical reaction force reached approximately 2.46 N for the snap-actuated robot and 1.53 N for the rigid-legged control. After subtracting the baseline force, the excess vertical impulse over the hopping phase was $0.106\ \text{N}\cdot\text{s}$ for the snap-actuated robot and $0.027\ \text{N}\cdot\text{s}$ for the rigid-legged control, corresponding to an approximately fourfold increase. This force-level comparison supports the interpretation that the snap-through limb converts motor-imposed boundary loading into impulsive mechanical output through rapid elastic energy release.

We also characterized the effective frictional interaction between the assembled robot and the tested substrates (Fig. S25B). Static robot–substrate friction coefficients were measured using the critical tilt-angle method, in which the substrate was gradually tilted until sliding began; the static friction coefficient was computed as $\mu_s = \tan\theta_c$, where θ_c is the critical tilt angle. Kinetic robot–substrate friction coefficients were measured by pulling the robot across each substrate with a force gauge and computing $\mu_k = F_{\text{pull}}/N$, where F_{pull} is the measured pulling force and N is the normal load. For outdoor grass and sand, where the tilt-angle method was not practical, we report the kinetic friction coefficient only.

The measured effective robot–substrate friction coefficients are summarized in Fig. S25D. These measurements define the substrate interaction conditions used in the locomotion experiments and support the comparison between the snap-actuated robot and the rigid-legged control across terrains. Together, the reaction-force and friction measurements provide a mechanism-level characterization of how the snap-through limb transmits impulsive forces to the environment under comparable contact conditions.

Teleoperation control in unstructured environments

To evaluate controllability and robustness in unstructured environments, we implemented a teleoperation system with a two-layer architecture (Fig. S26). A handheld game controller provides user input to a host PC, which maps joystick commands and mode selections to predefined gait parameters (e.g., hopping and swimming) at ~ 50 Hz. Command parameters are transmitted to the onboard microcontroller via Bluetooth Low Energy (BLE) at an effective update rate of 20–30 Hz. The onboard controller (Seeed Studio XIAO) runs a local loop at 200 Hz to generate synchronized PWM signals for the two servo motors using the most recent received command. This separation enables flexible high-level mode switching while maintaining deterministic low-level motor actuation.

Light-guided locomotion using onboard sensory feedback

To demonstrate that the snap-actuated platform can be integrated with simple onboard sensing and feedback control, we performed a light-guided locomotion experiment. The robot was equipped with two light-dependent resistors (LDRs), one on each side of the robot body. The relative light intensity measured by the two LDRs was used to switch the activation of the left and right snap-actuated limbs.

The control rule was designed to steer the robot toward the light source. When the left LDR received stronger illumination, the right limb was activated to turn the robot left. When the right LDR received stronger illumination, the left limb was activated to turn the robot right. When both LDRs detected comparable illumination from the light source, both limbs were activated to drive the robot forward. Thus, the same differential limb-actuation strategy used for turning was coupled to onboard sensory feedback.

We tested the light-guided behavior in three independent runs starting from different initial poses. In all three trials, the robot reoriented and moved toward the same light source (Fig. 6E and Movie S9). These experiments demonstrate that the snap-actuated limb architecture can support simple sensor-guided locomotion using onboard feedback. The goal of this experiment is not to demonstrate complex autonomous navigation, but to show that the proposed snap-actuated platform can be combined with lightweight sensing and simple feedback control for environment-responsive locomotion.

Additional materials and data for the robot locomotion in unstructured environments

The custom stairs used in Fig. S27 have a stair height of 2 cm (0.6 body height) and were fabricated from acrylic plates (3 mm thickness, Kinlink) with 3D-printed supports (Formlabs White Resin V5). Sand-playground experiments were conducted at the Mars Yard (Ford Robotics Building, University of Michigan), and outdoor swimming tests were conducted at Olson Park (Ann Arbor, MI). During field swimming trials, wind speeds reached 12–13 m/s (Fig. S28), and the robot remained controllable with stable swimming trajectories.

Comparison with state-of-the-art untethered soft robots

We frame the performance of our robots in the context of state-of-the-art soft robots, focusing exclusively on untethered systems. We define an “untethered” soft robot as one that carries both its power supply and control electronics on board and does not rely on any electrical tether during operation.

In Fig. 5H, we have already compared our robot with the state-of-the-art robots and the comparable animals. The data and references used in Fig. 5H are listed in Tables S4, S5 and S6.

Supplementary Figures

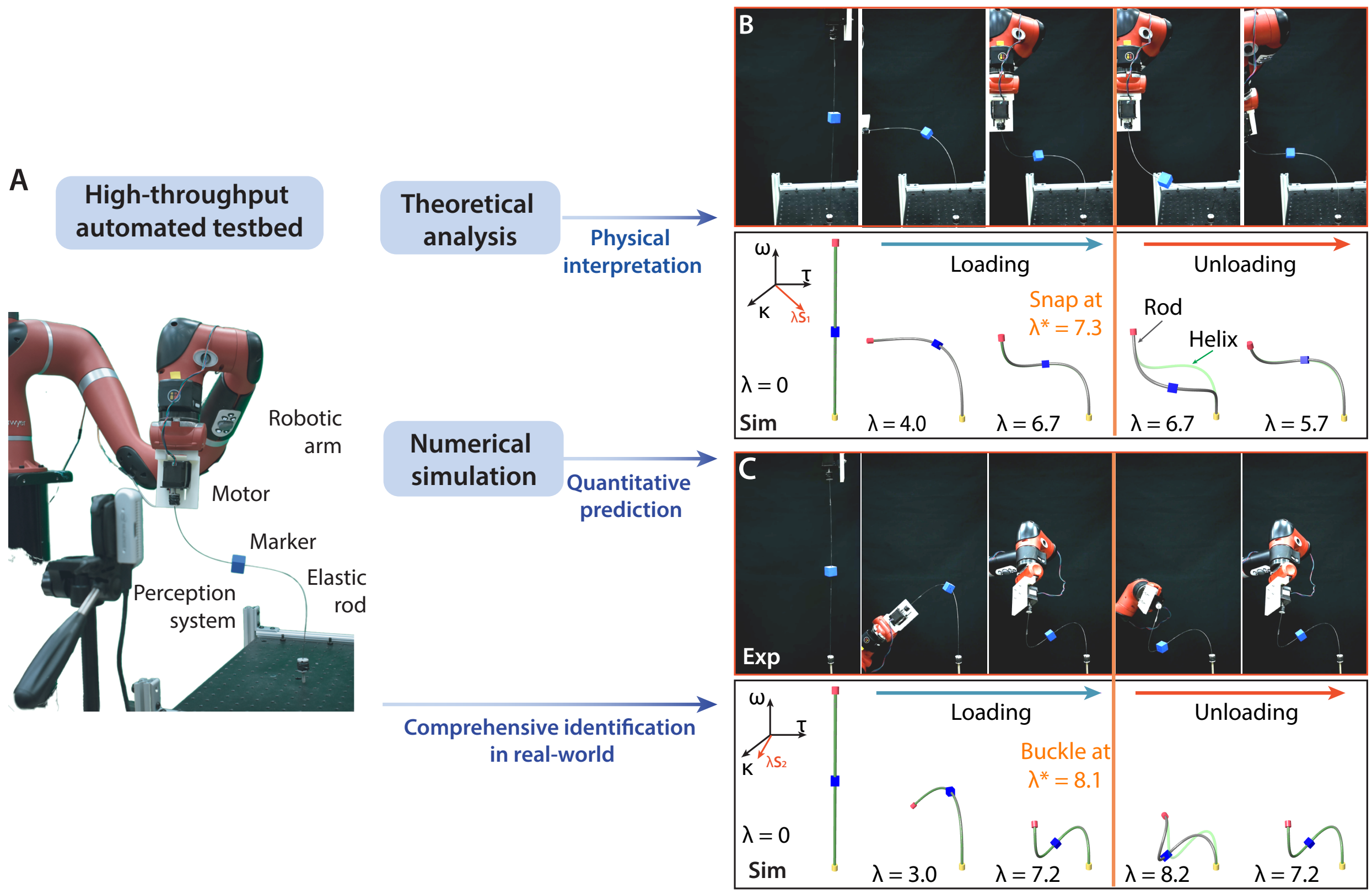

Figure S1: **A theory–simulation–high-throughput experimental framework reveals two distinct buckling pathways in helical filaments.** (A) Overview of the integrated framework combining theory, dynamic simulation, and automated high-throughput experiments. Representative loading–unloading cycles along rays in (κ, τ, ω) space show two post-buckling responses from stable helical equilibria: (B) snap-through to a distant non-helical configuration and (C) continuous buckling to a nearby non-helical configuration. Automated experiments (top) and simulations (bottom) show consistent behavior. Unit loading directions are $\mathbf{S}_1 = [0.704, 0.700, 0.120]$ and $\mathbf{S}_2 = [0.901, 0.407, 0.148]$.

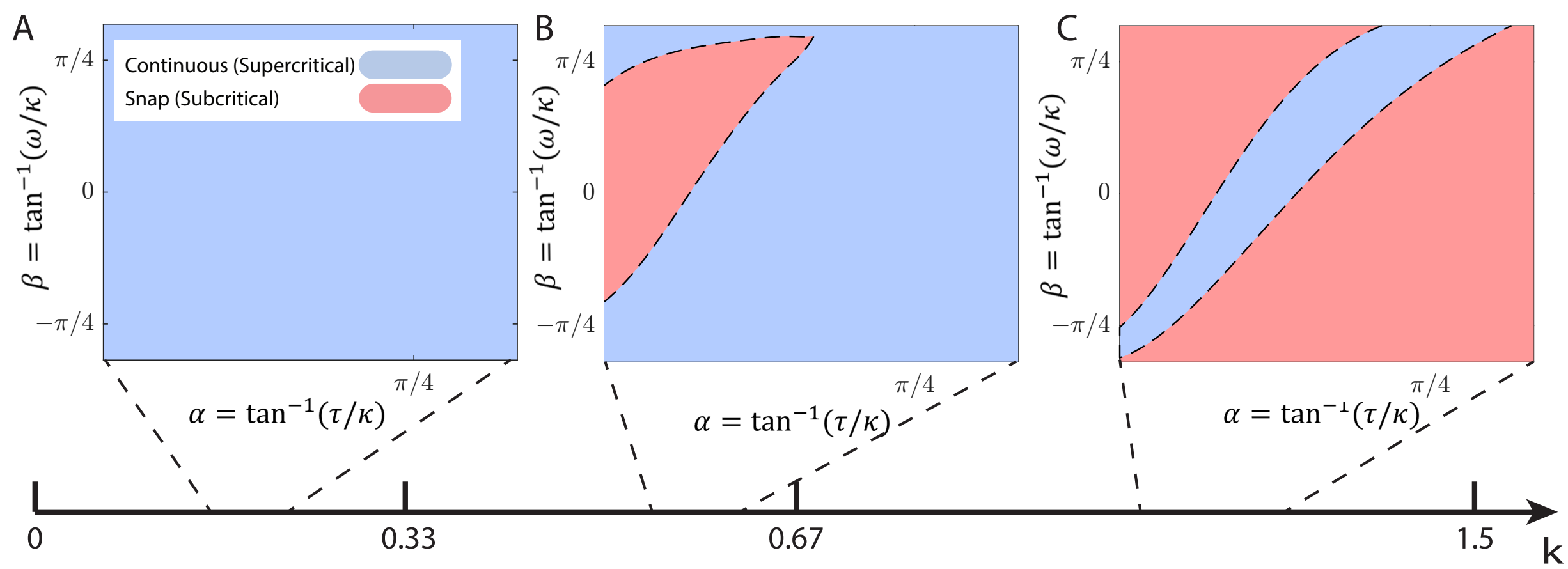

Figure S2: **Influence of material properties on buckling mode selection.** Phase diagrams showing loading directions, parameterized by (α, β), that lead to snap-through (subcritical) versus continuous (supercritical) helical buckling for different values of the stiffness ratio $k = k_b/k_t$. (A) Representative phase diagram for $k \in (0,\ 0.33]$. (B) Representative phase diagram for $k \in (0.33,\ 0.67]$. (C) Representative phase diagram for $k \in (0.67,\ 1.5]$.

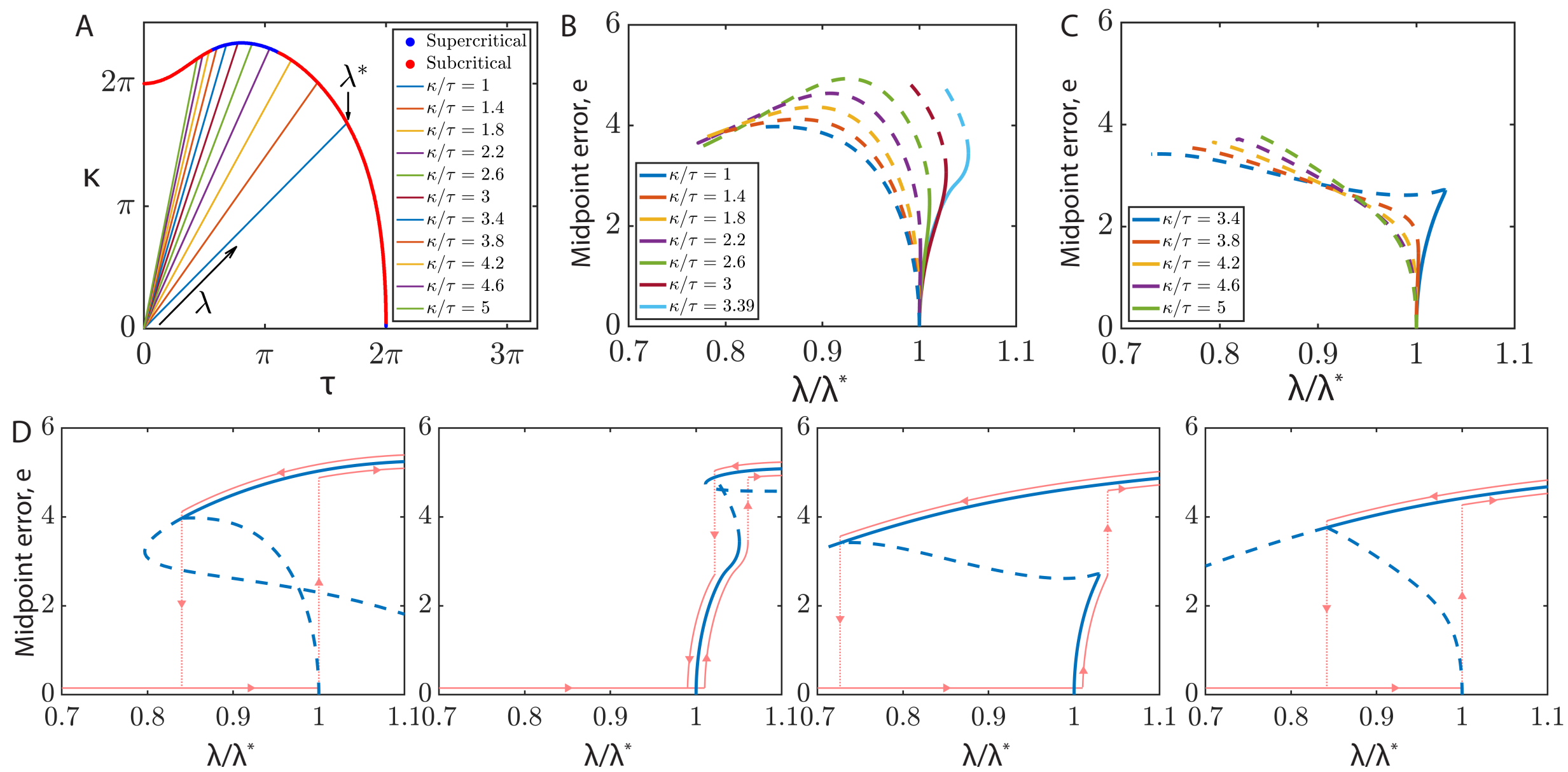

Figure S3: **Visualization of post-buckling behavior.** (A) Search directions **S** in the $\omega = 0$ plane, with the onset of buckling points λ^* at supercritical (blue) and subcritical (red) bifurcations. (B–C) Normalized midpoint error e between post-buckled non-helical rod configurations and helical rod configurations as a function of normalized distance λ/λ^* along search directions. Solid curves indicate stable solutions, dashed curves indicate unstable solutions, and each curve represents two symmetric solution branches emanating from a pitchfork bifurcation at $\lambda/\lambda^* = 1$. (D) Secondary subcritical pitchfork bifurcations occur when the two symmetric solution branches emanating from $\lambda/\lambda^* = 1$ converge. Directed red lines indicate the expected post-buckling behavior of a physical rod, with solid lines representing continuous motion and dotted lines representing jumps.

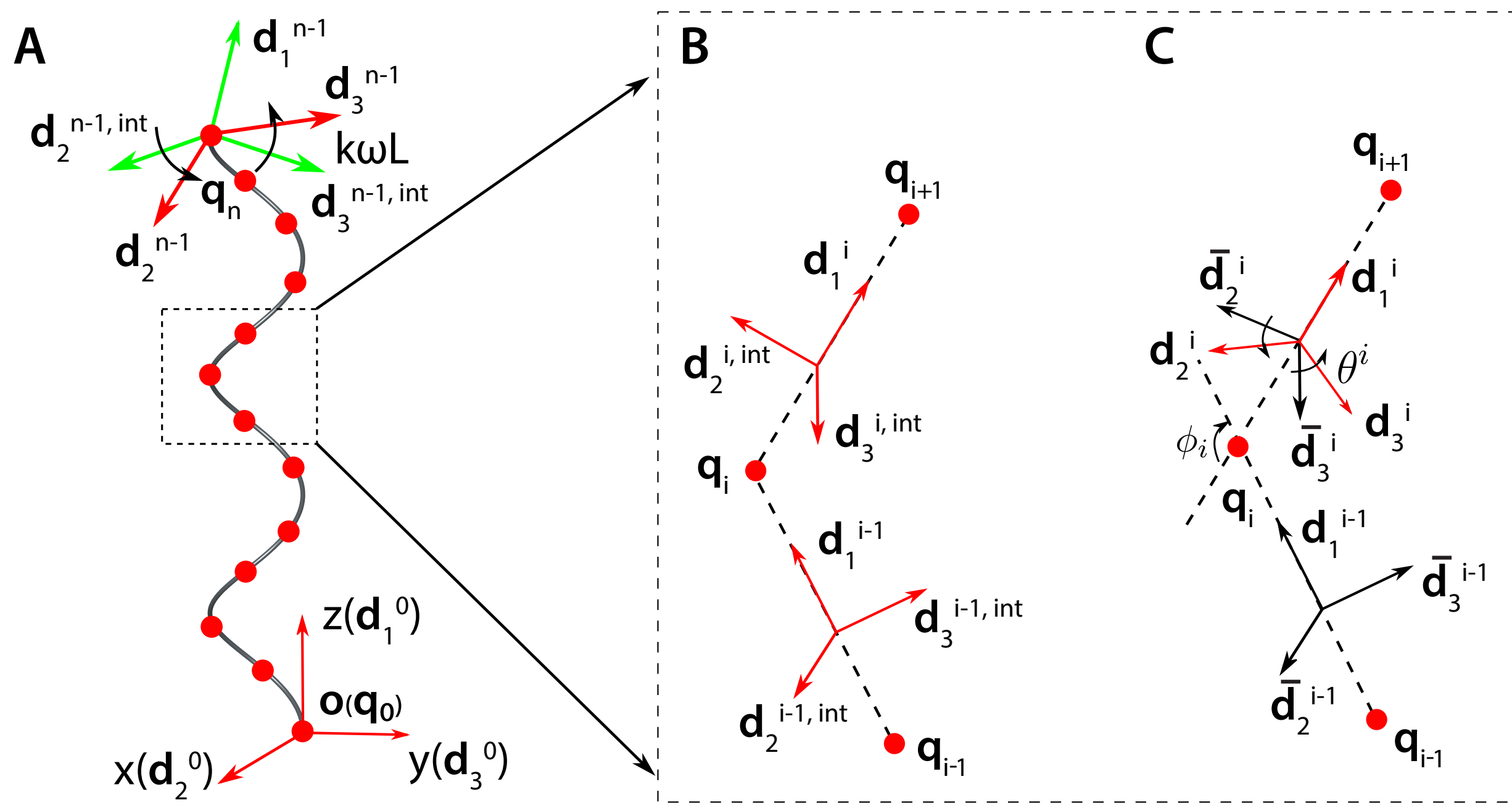

Figure S4: **Discrete schematic of helical filament for simulation.** (A) The schematic of the filament and illustration of the boundary conditions at two ends; (B) schematic diagram demonstrating parallel transport of the reference frame; (C) schematic showing the tuning angle ϕ_i, twisting angle θ_i, reference frames, and material frames along edges $\mathbf{e}^{i-1}$ and $\mathbf{e}^{i}$.

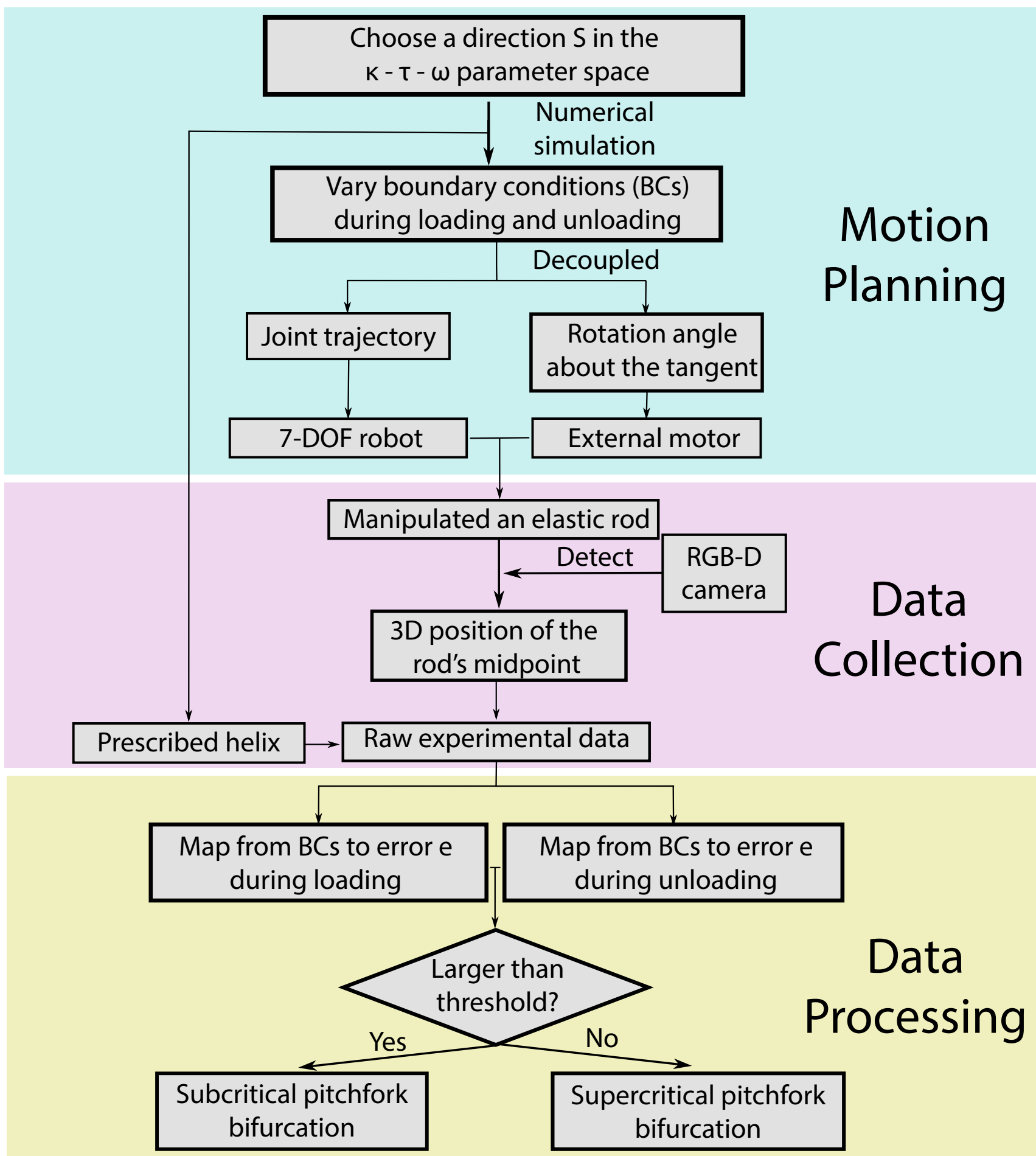

Figure S5: Flowchart of the automated robotic system for experimentally analyzing buckling of helical rod configurations.

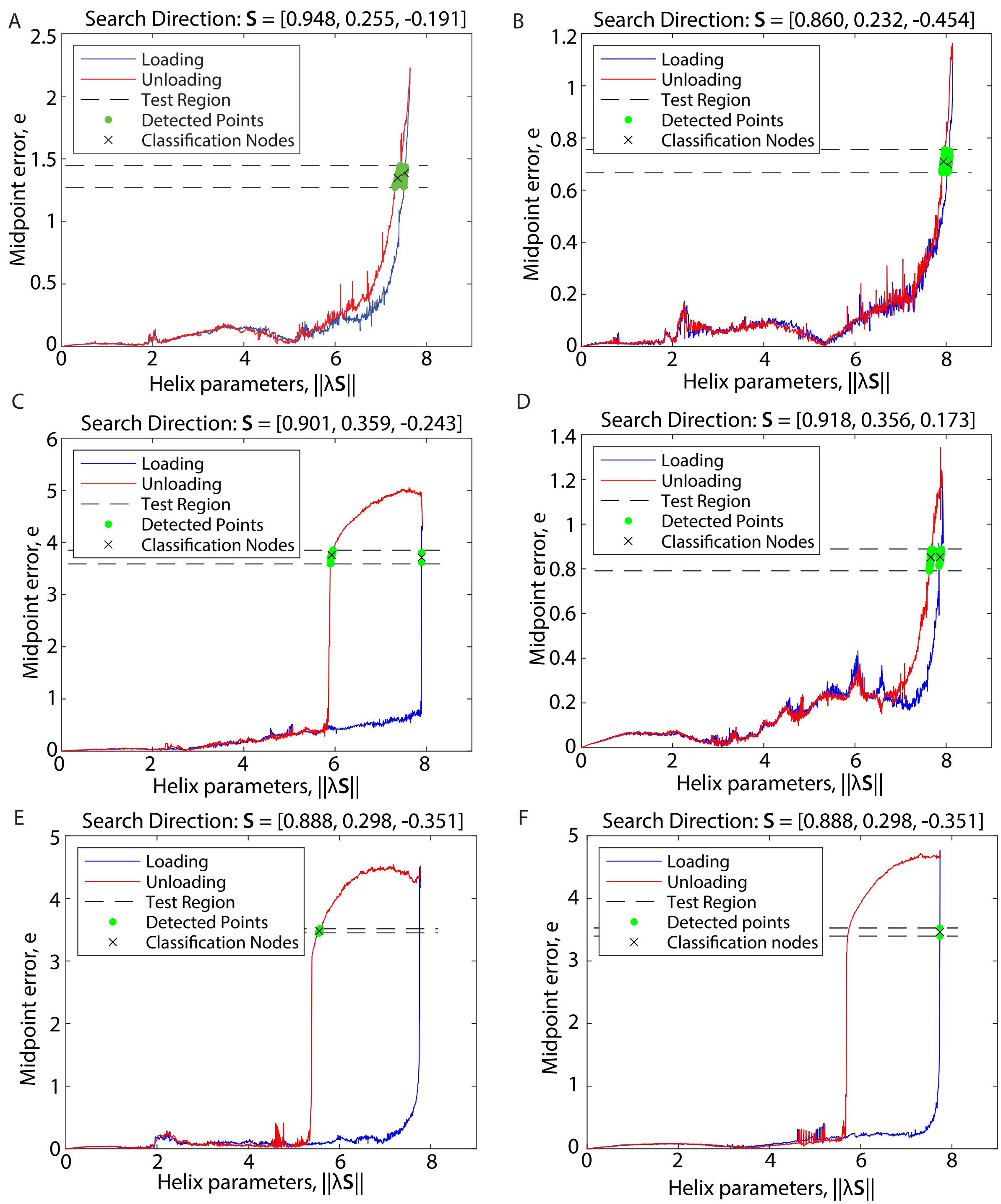

Figure S6: **Experimental data and the error interval** $[\bar{e}-\Delta e, \bar{e}+\Delta e]$ **used to classify instabilities** as either supercritical bifurcations (A-C) or subcritical bifurcations (D-F).

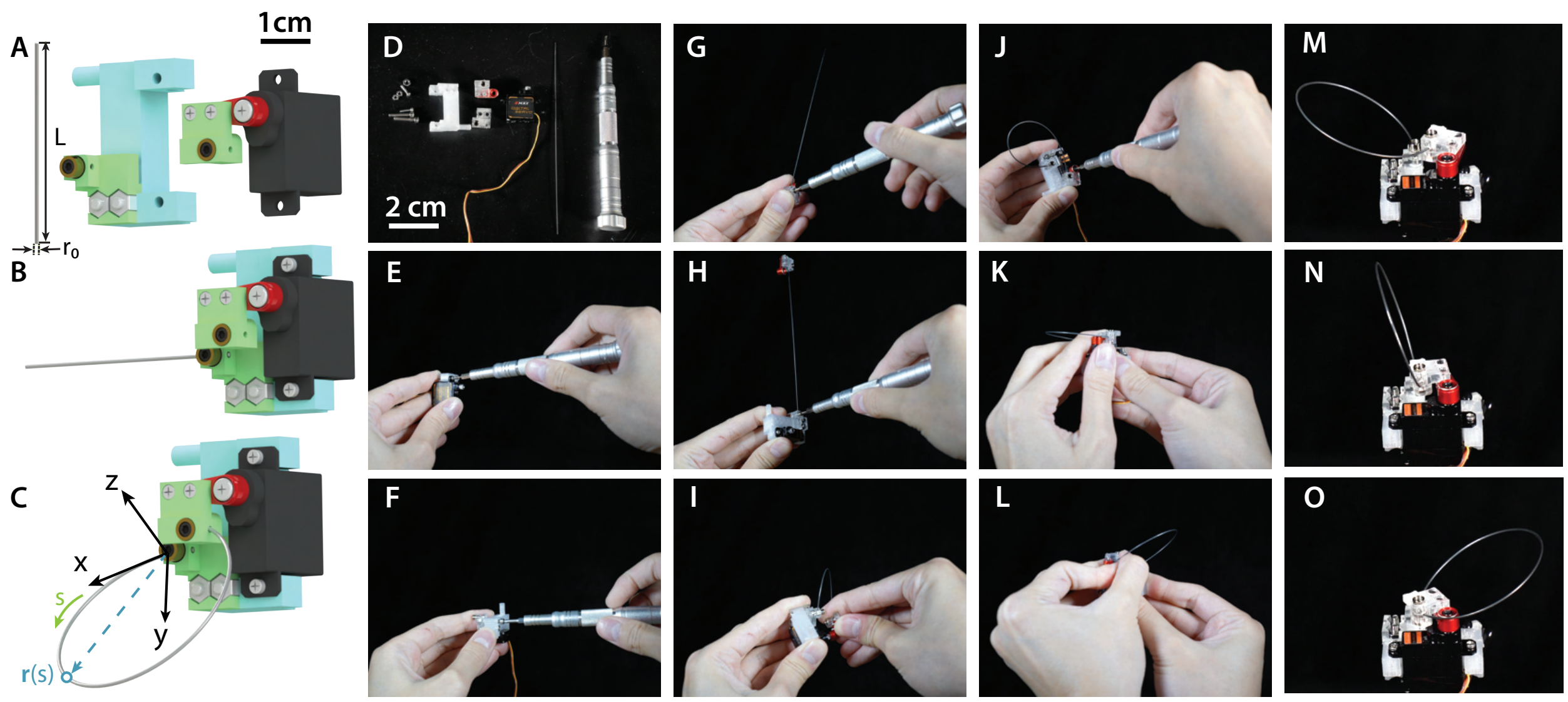

Figure S7: **Manufacturing of the snap-actuated helical limb.** Fabrication of the elastic limb: (A) we 3D-print the holder and connectors and cut a nitinol superelastic rod to the desired length. (B) One end of the rod is inserted into a connector and clamped to fix its position and orientation. (C) The other end is bent and inserted into a prescribed hole on the holder, so that both ends are clamped and a helical configuration is formed. Assembly steps: (D) After preparing the required materials and tools (3D-printed holder, 3D-printed connectors, nitinol rod, screwdrivers, a servo motor, screws, and nuts), (E–F) we mount the servo motor on the holder, and (G–H) clamp one end of the rod to the connector and the other end to the holder. (I–J) The connector is aligned with the motor shaft and secured with screws, yielding a complete elastic limb assembly. Demonstration of the limb: When the servo motor rotates, the helical rod snaps through from a stable helical configuration to a non-helical configuration. (M–O) Photographs of a fully manufactured elastic limb.

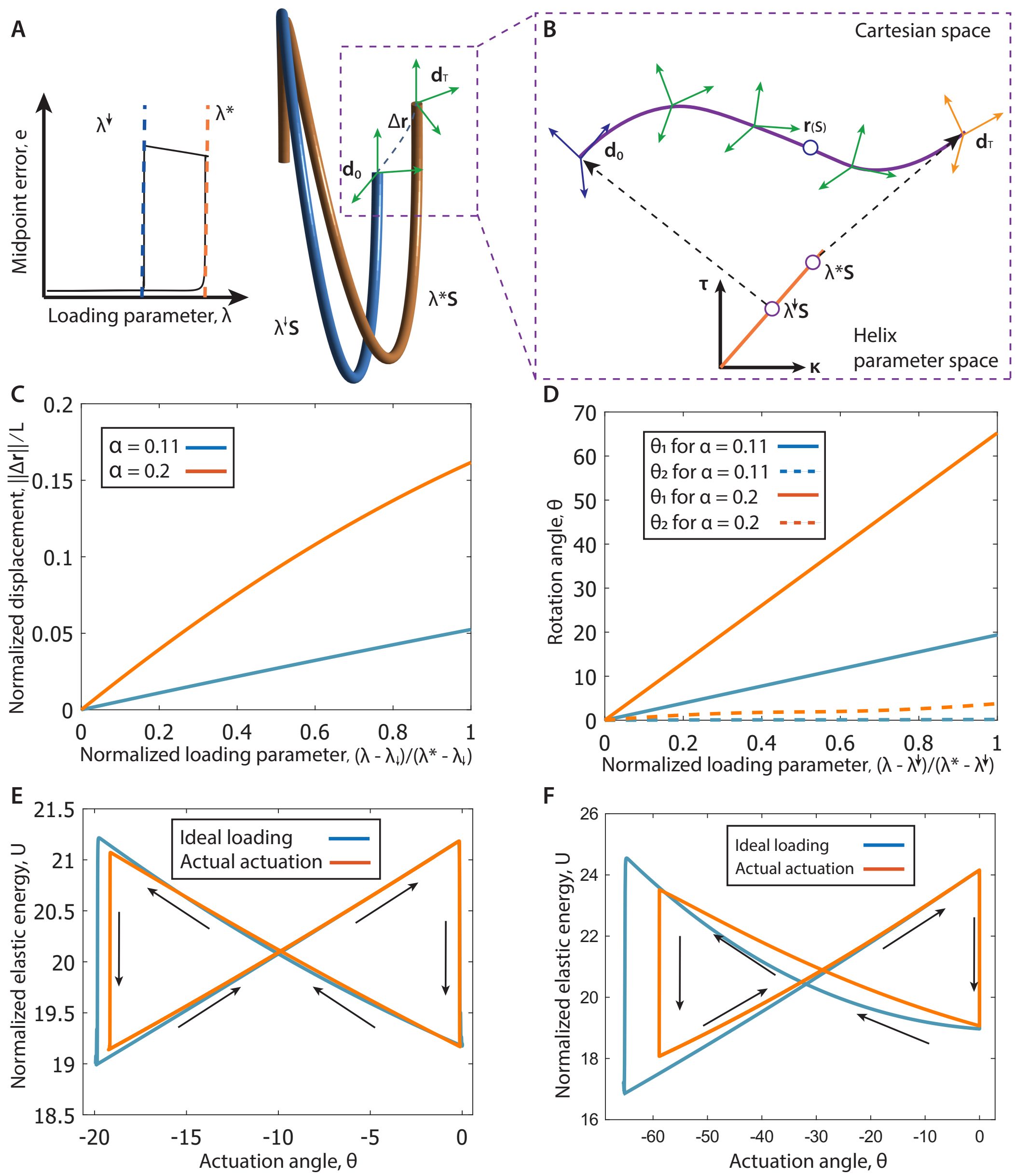

Figure S8: **Comparison between ideal loading and actual actuation of the helical limb.** (A) Critical helical shape (helical configuration immediately before snap-through) and reset helical shape (helical configuration immediately after snap-back). (B) Ideal loading path from the reset helical shape to the critical helical shape during loading. (C) Normalized displacement of the actuated end along this loading path. (D) Rotation angles of the actuated end decomposed along the principal axis $\mathbf{e}_1$ and the secondary axis $\mathbf{e}_2$. (E–F) Comparison of the normalized system energy over a full snap-through cycle under the ideal loading path and under the actual actuation, for critical helical shapes with helix angles (E) $\alpha = 0.11$ and (F) $\alpha = 0.20$.

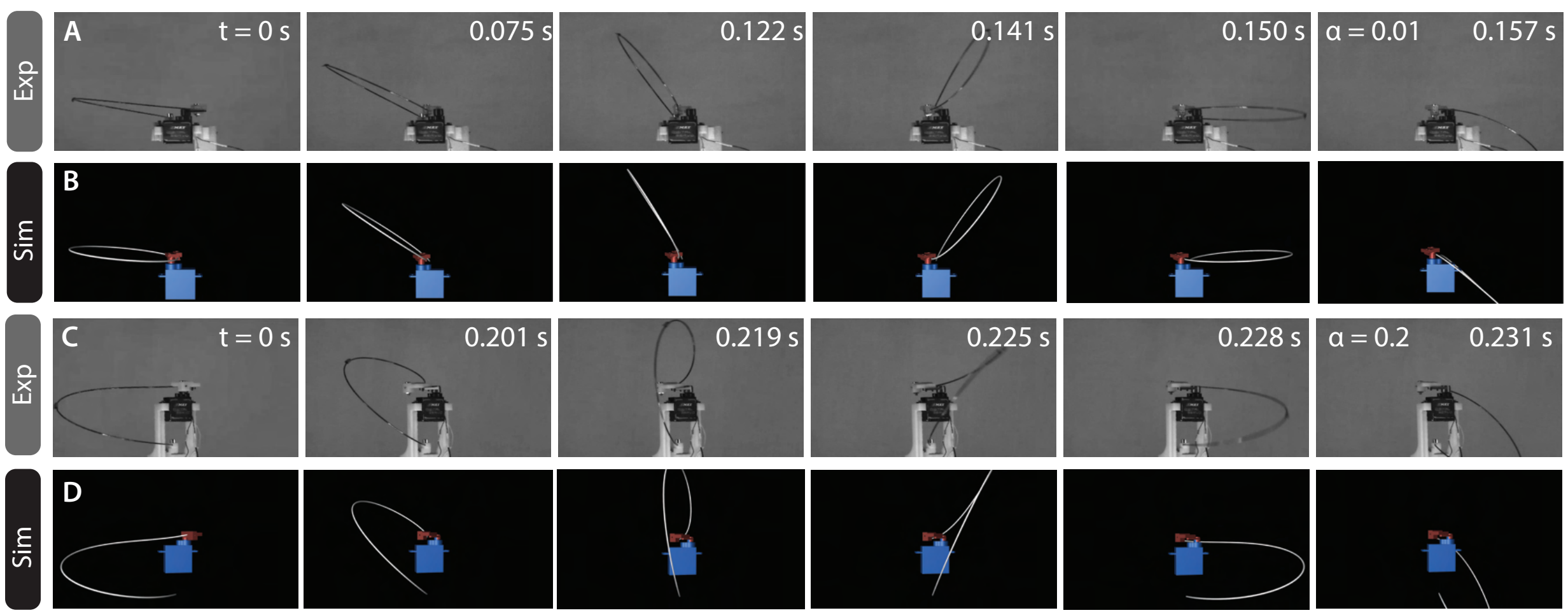

Figure S9: **Representative snapshots comparing experiments and dynamic simulations for limbs with different critical helical shapes.** (A) Experimental snapshots and (B) dynamic simulations of snap-through buckling for a limb with critical helical angle $\alpha = 0.01$. (C) Experimental snapshots and (D) dynamic simulations for a limb with critical helical angle $\alpha = 0.20$.

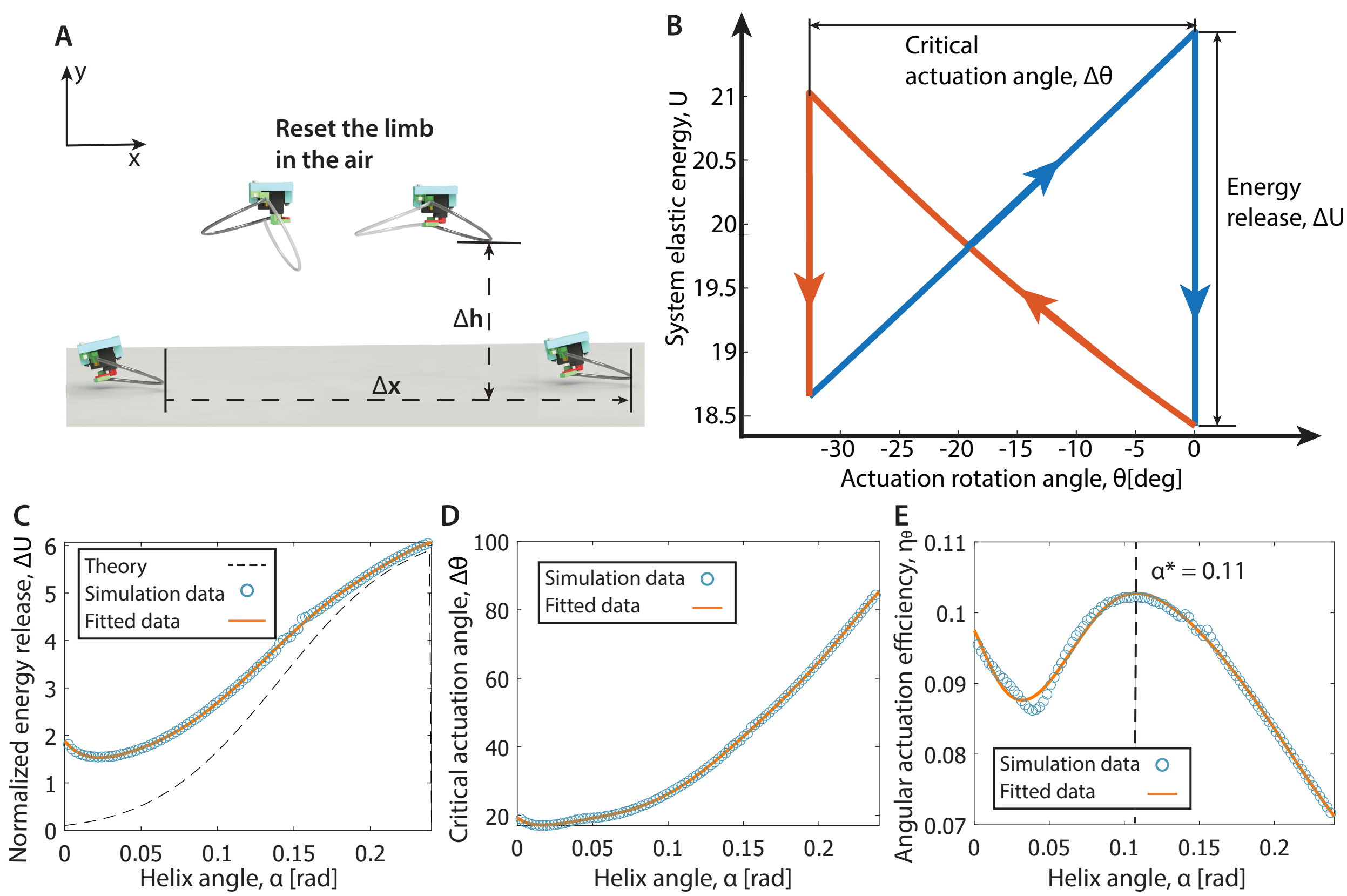

Figure S10: **Selection rule for the critical helical configuration.** (A) Schematic of a single hop of the robot with the elastic limb. (B) Illustration of one full snap-through–snap-back cycle for helix angle $\alpha = 0.11$ along the actuation path. (C) Comparison of snap-through energy release for different critical helical shapes, computed from static theoretical analysis and dynamic simulations. (D) Critical actuation angle of different critical helical shapes. (E) Angular actuation efficiency η_θ for different critical helical shapes.

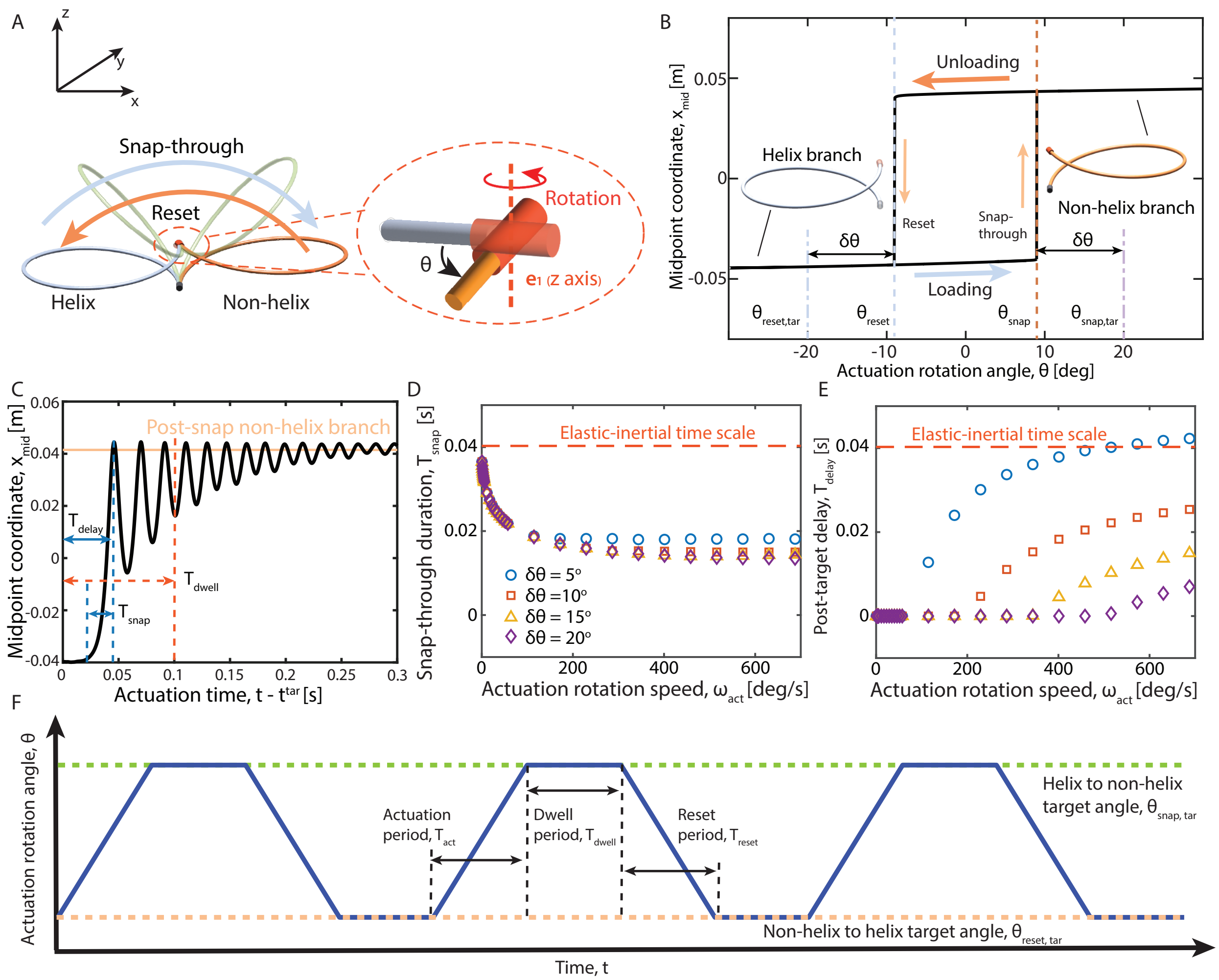

Figure S11: **Command design for repeatable snap-through/reset.** (A) Schematic of the limb-level snap-through/reset cycle driven by rotation of the actuated end. (B) Quasi-static response defining the snap-through threshold, reset threshold, and command target angles. (C) Dynamic response used to define the snap-through duration T_{snap}, post-target delay T_{delay}, and dwell interval T_{dwell}. (D, E) Transition timing under different actuation speeds and command margins. T_{snap} remains bounded and comparable to the elastic-inertial time scale, whereas T_{delay} decreases as the command target is moved farther beyond the instability threshold. (F) Idealized servo command profile: the controller drives beyond the snap-through target, dwells, and then reverses for reset, avoiding high-bandwidth tracking of the discontinuous transition.

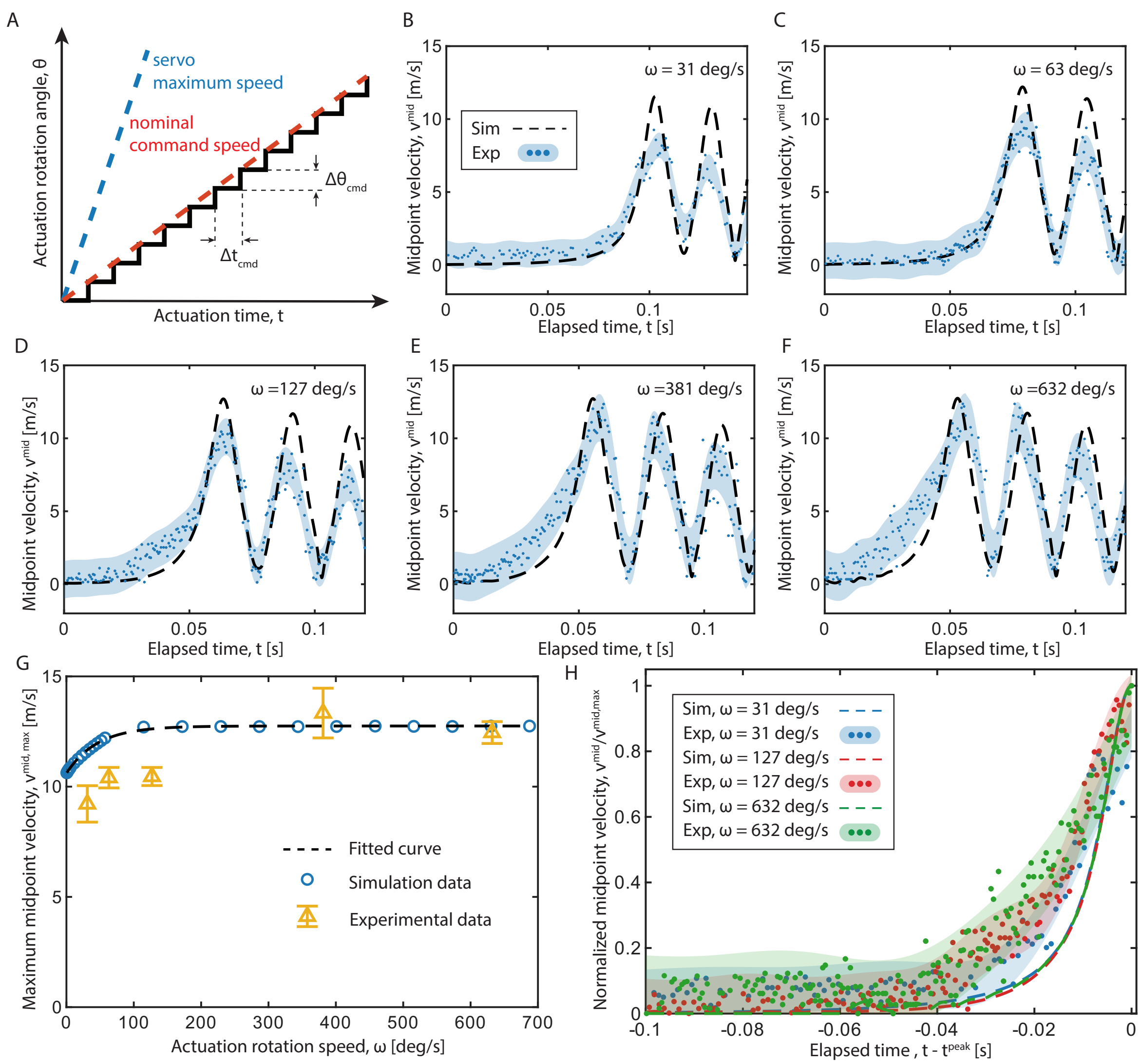

Figure S12: **Finite-speed actuator-level experiments and simulations.** (A) Experimental command protocols for different servo actuation speeds. (B–F) Simulated and experimental midpoint-velocity trajectories at different actuation speeds. (G) Maximum midpoint velocity $v_{\text{mid,max}}$ as a function of measured actuation speed. (H) Normalized midpoint-velocity growth before the first peak. Finite-speed actuation changes the dynamic response but does not suppress the selected snap-through pathway over the tested range.

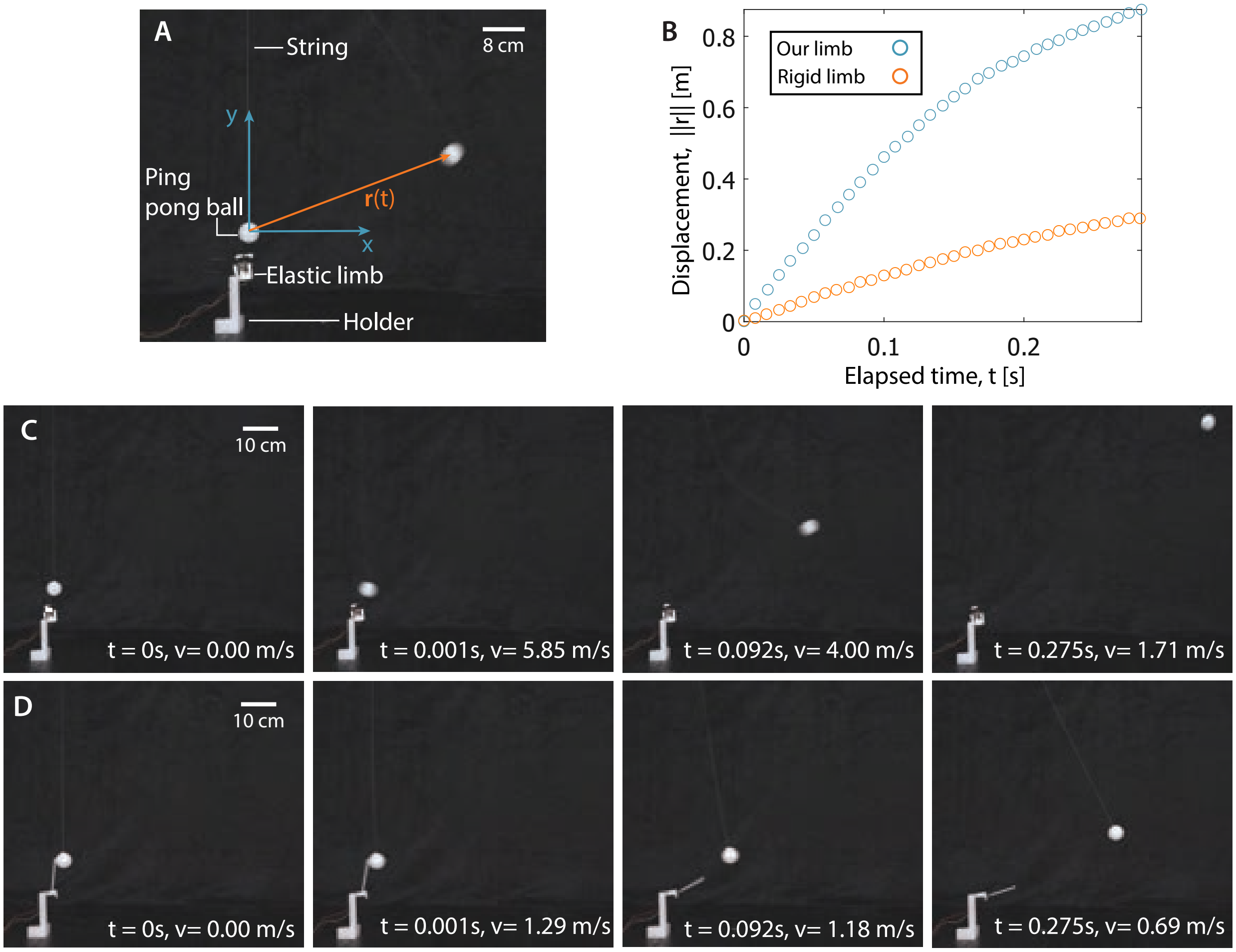

Figure S13: **Demonstration of the snap-through actuated limb hitting a hanging ping-pong ball.** (A) Experimental setup for the ping-pong ball impact test. (B) Comparison of displacement–time histories after impact for the snap-actuated limb and a rigid limb. (C) Sequential snapshots of (C) the snap–actuated limb hitting the ball and (D) the rigid limb hitting the ball.

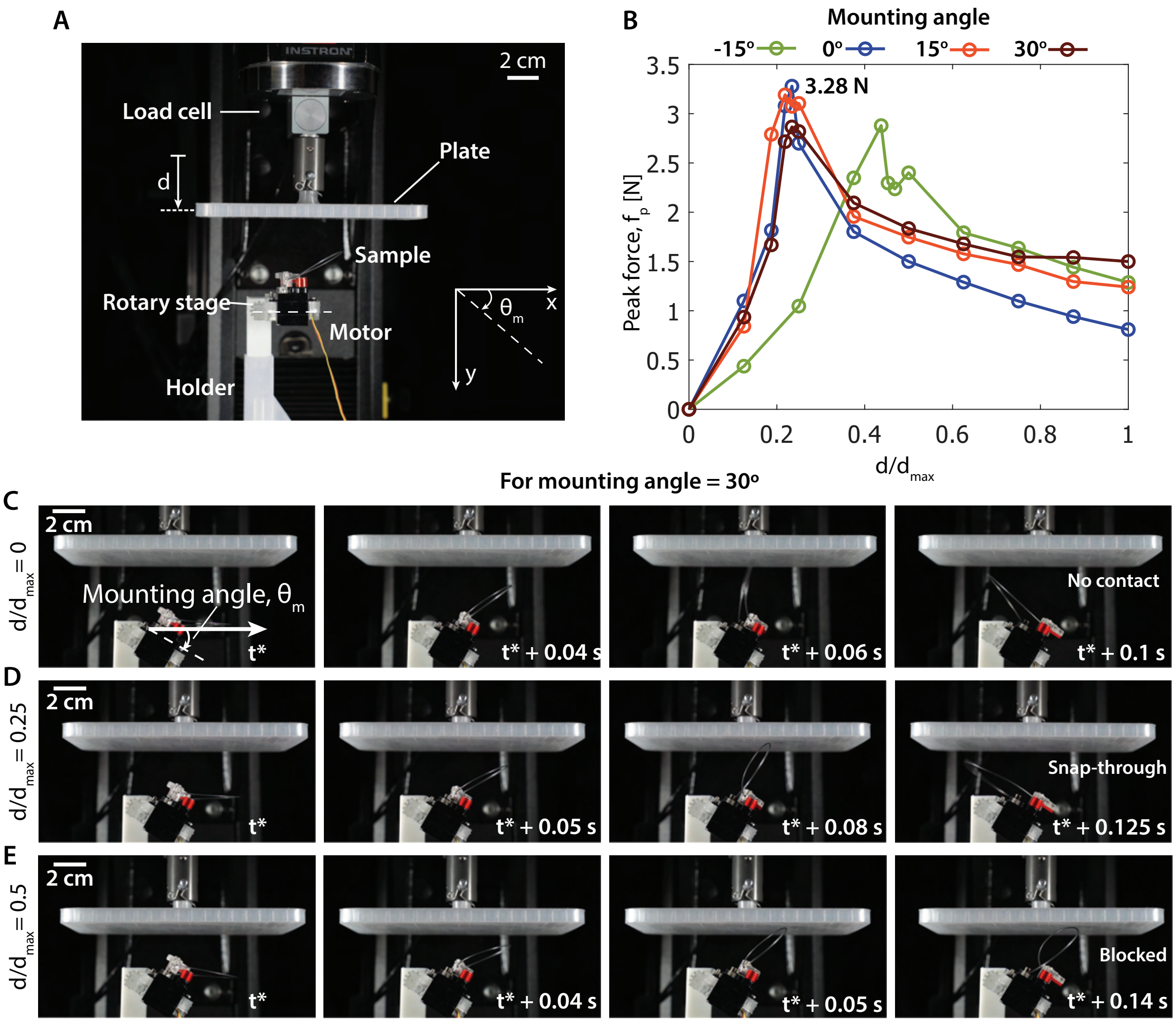

Figure S14: **Measurement of the peak blocking force by constrained actuation of the elastic limb.** (A) Experimental setup used to measure the force exerted by the elastic limb as it reconfigures against a fixed plate. (B) Measured peak blocking force for different fixed mounting angles and normalized distances between the limb and the plate, $d/d_{\max}$. Representative snapshots of the measurements for (C) $d/d_{\max} = 0$, (D) 0.25, and (E) 0.5, respectively.

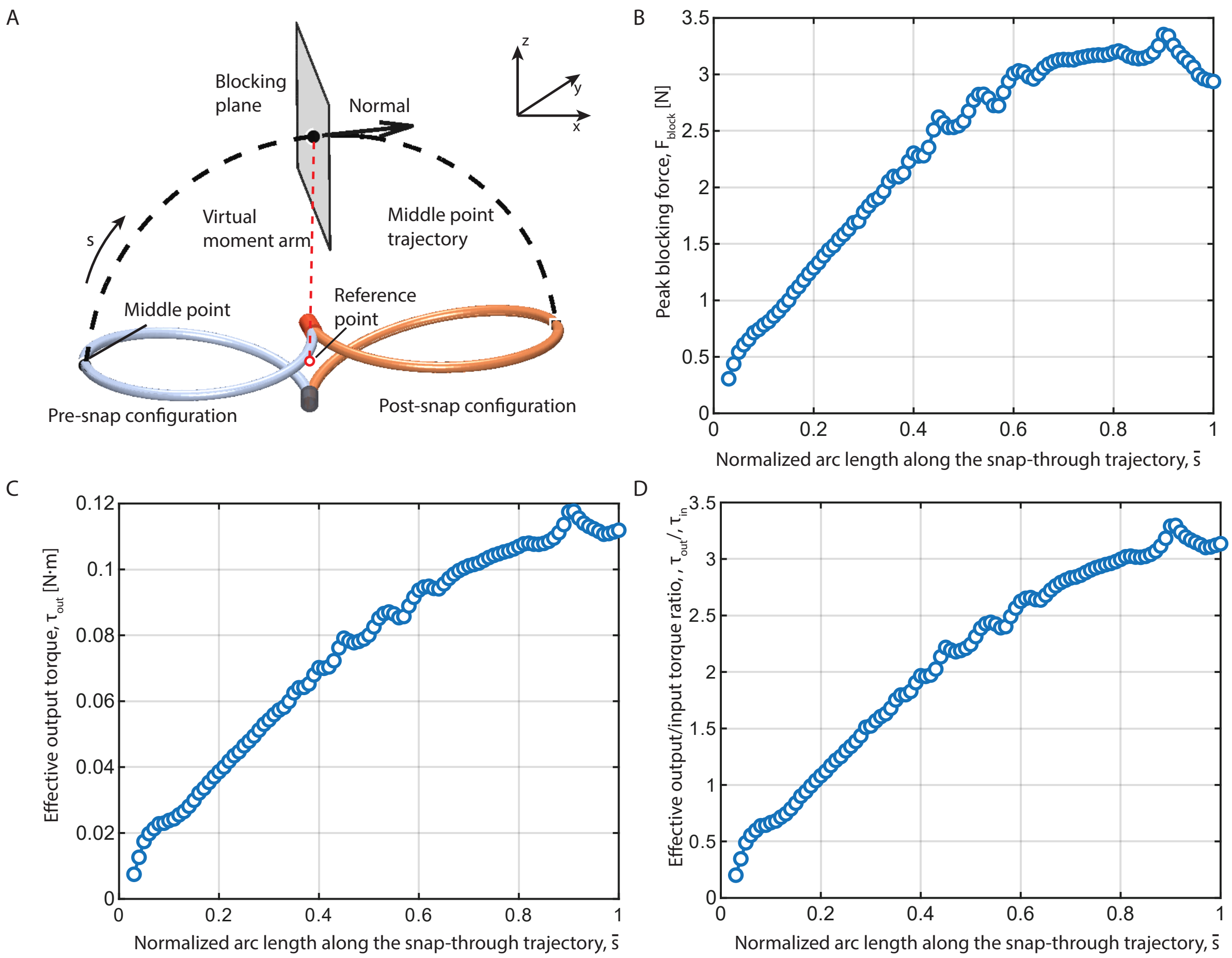

Figure S15: **Contact-constrained snap-through and blocking-force simulation.** (A) Simulation setup. The unconstrained snap-through trajectory is first computed, and blocking planes are placed along the midpoint trajectory to locally constrain the snap-through motion. The virtual moment arm is defined from the base reference point to the blocking-contact location. (B) Peak blocking force $F_{\text{block,max}}$ as a function of normalized arc length $\bar{s}$ along the snap-through trajectory. The contact-force magnitude is smoothed using a 1 ms moving-average window before extracting the peak value. (C) Effective output moment τ_{out} computed about the base reference point and projected onto the dominant snap-through reconfiguration axis. (D) Effective output/input torque ratio $R_\tau = \tau_{\text{out}}/\tau_{\text{in}}$. The simulation provides a controlled example of environment-side reaction-force characterization under contact-constrained snap-through.

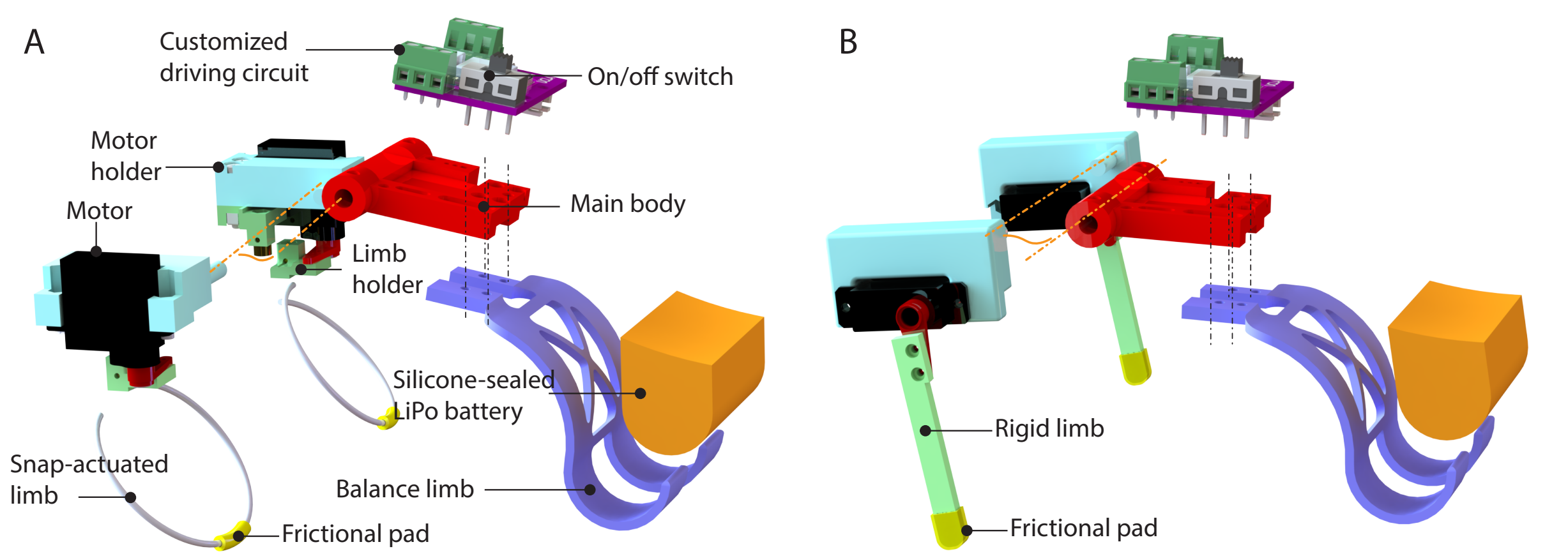

Figure S16: **Design of the untethered hopping robot and the rigid-legged robot.** (A) Exploded render of the untethered hopping robot. Key components include the snap-actuated limbs, motors, silicone-sealed LiPo batteries, and the customized driving circuit. (B) Exploded render of the untethered rigid-legged robot. The soft elastic limbs of the untethered hopping robot are replaced by rigid limbs.

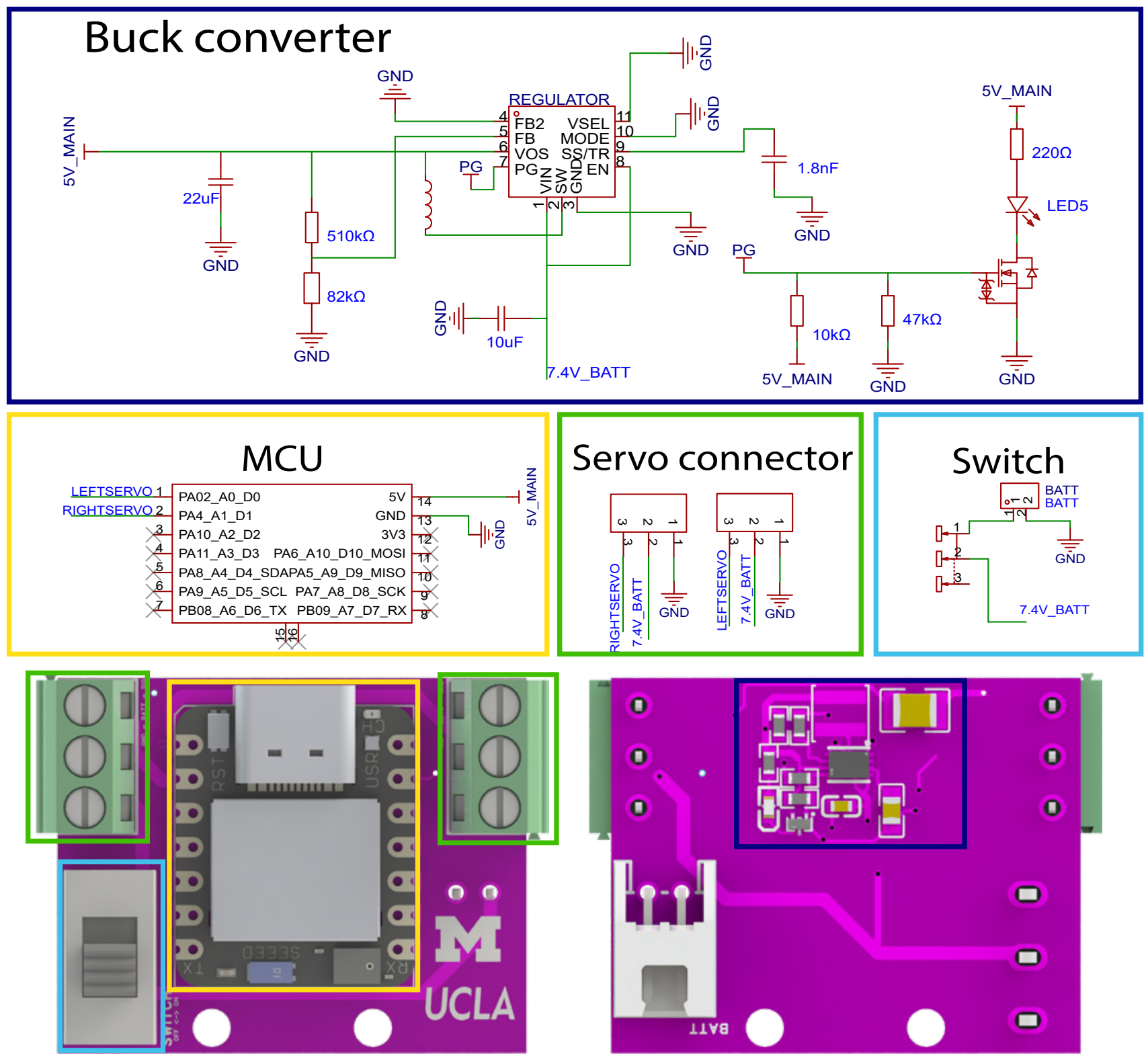

Figure S17: **Custom PCB and power/control architecture for the untethered robot.** The board uses a split-rail design: a buck converter generates a regulated 5 V rail to power the microcontroller and logic, while the two servo motors are powered directly from the 7.4 V (2S) LiPo battery to preserve torque under load. The microcontroller outputs independent PWM command signals to each servo through a dedicated driver/interface stage. Status LEDs, a power switch on the main battery line, and passive filtering/decoupling networks improve robustness by stabilizing the supply rails and maintaining signal integrity during high-current transients.

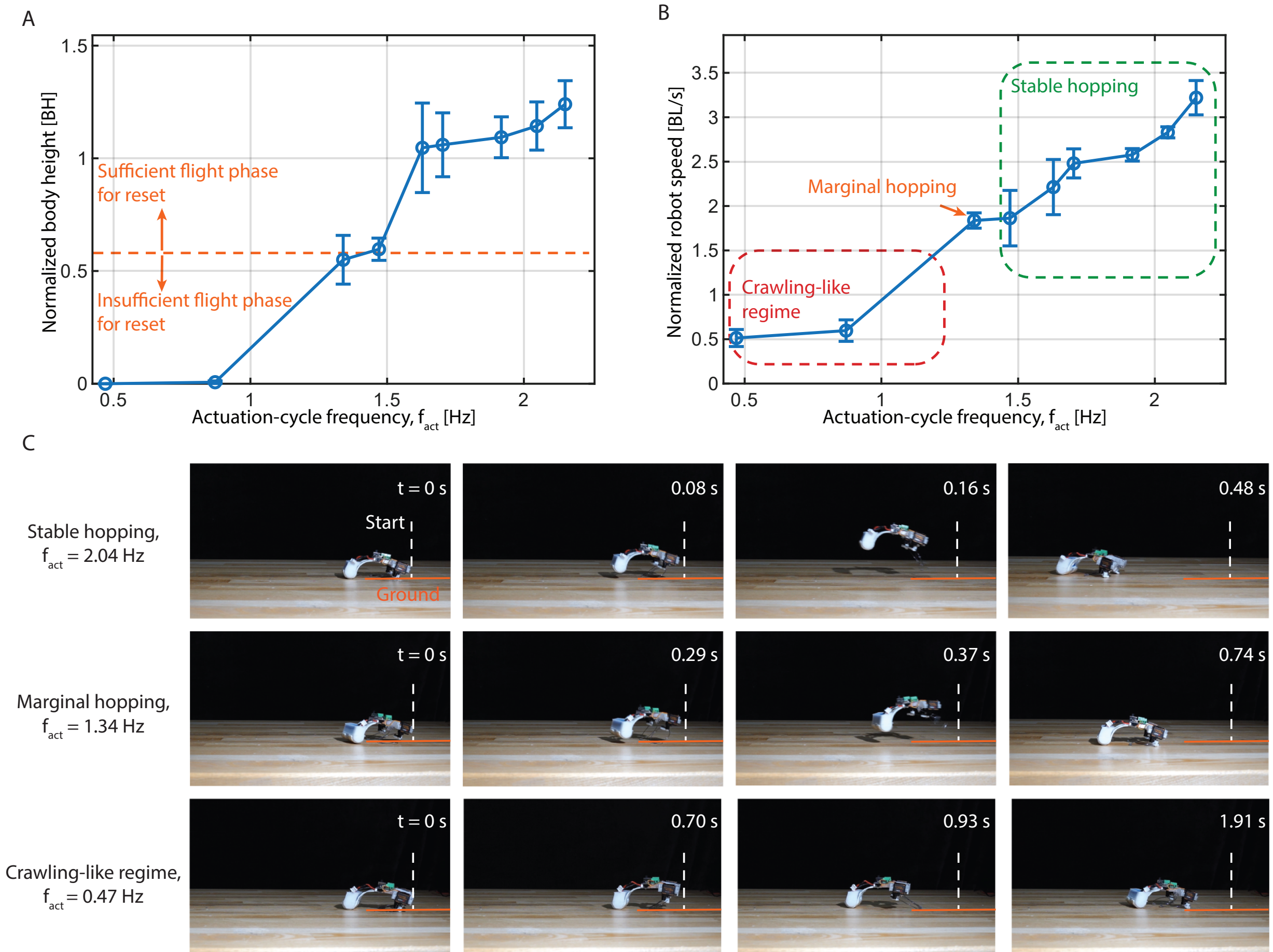

Figure S18: **Actuation-cycle frequency dependence of robot hopping performance.** (A) Maximum normalized body height as a function of actuation-cycle frequency. The dashed line marks the empirical transition between insufficient and sufficient flight phase for limb reset. (B) Normalized forward speed as a function of actuation-cycle frequency. The robot transitions from a crawling-like contact regime at low frequency to marginal hopping and then stable hopping at higher frequency. (C) Representative snapshots of stable hopping, marginal hopping, and crawling-like low-frequency motion. The white dashed line marks the initial body position, and the orange line indicates the ground. Error bars in (A,B) indicate standard deviation over repeated trials.

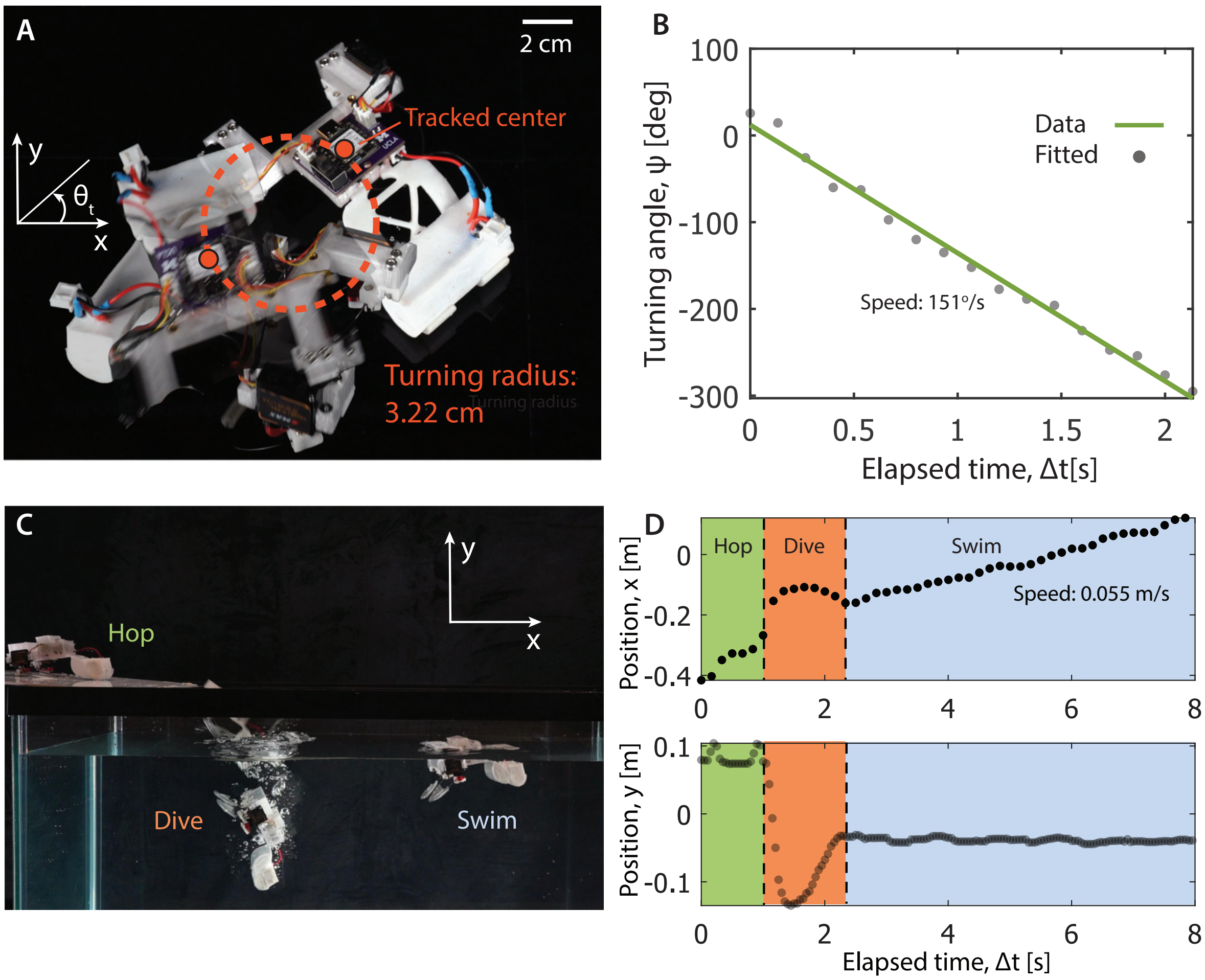

Figure S19: **Representative tracked robot position and orientation in turning and swimming gaits.** (A) Visual illustration of the minimal turning radius ≈ 3.22 cm (0.29 BL) of the untethered hopping robot. (B) Tracked turning angle of the robot as a function of elapsed time. (C) Visual illustration of the robot's amphibious swimming performance. (D) Tracked position of the robot over time during swimming.

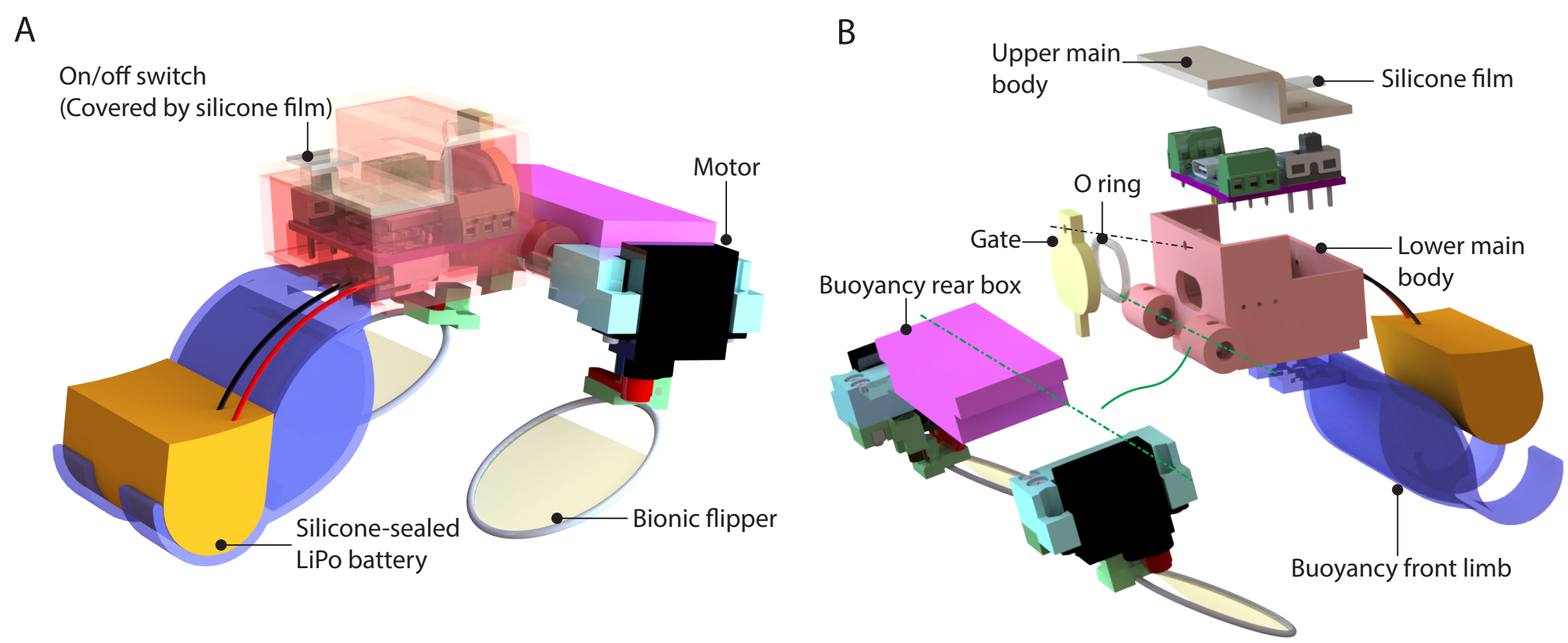

Figure S20: **Design of the untethered amphibious robot** (A) Design of the untethered amphibious robot. Key components include the bionic flippers, waterproof driving circuit body, silicone-sealed LiPo battery, and motors. (B) Exploded render of the untethered amphibious robot.

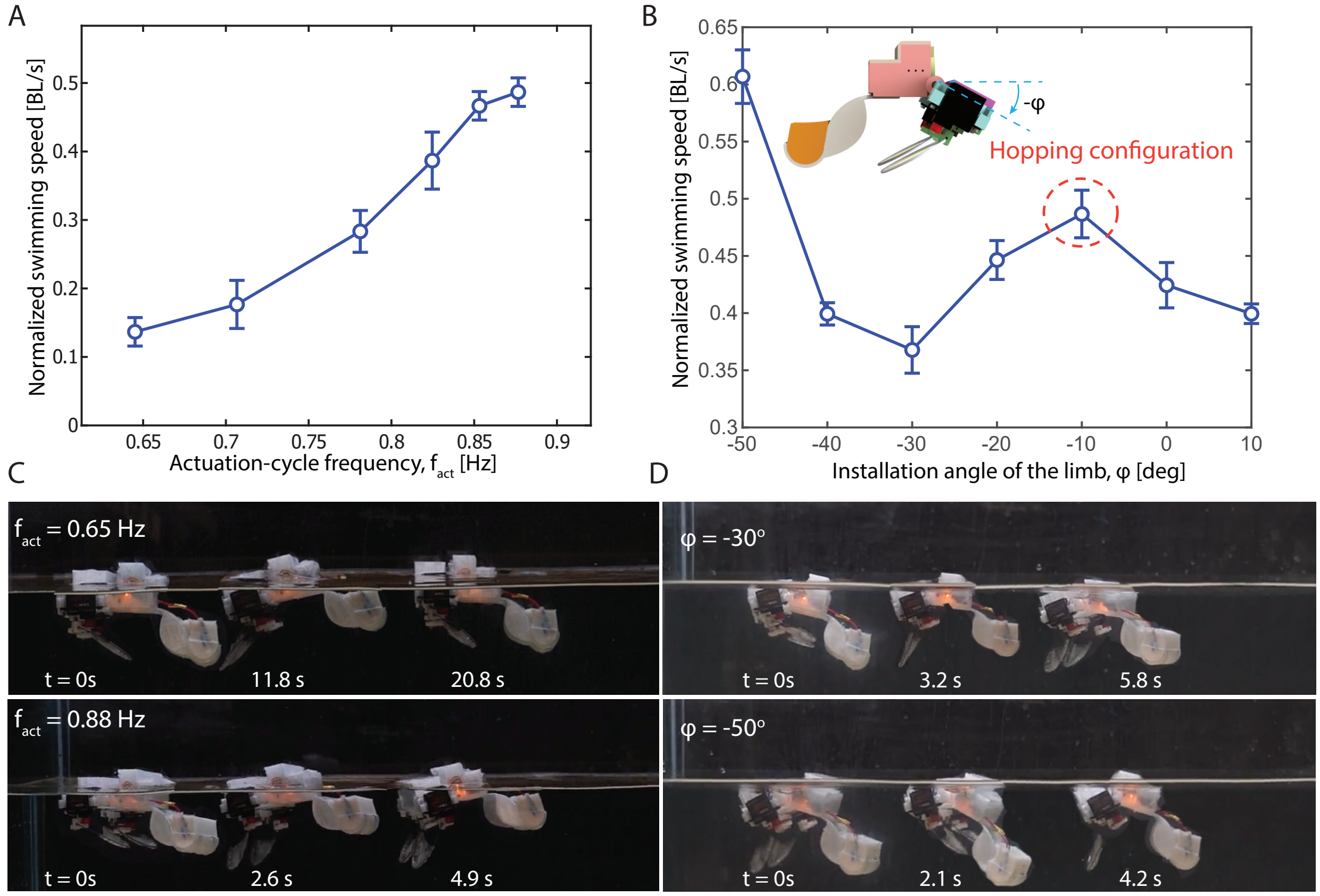

Figure S21: **Exploratory aquatic-locomotion study.** (A) Normalized swimming speed as a function of actuation-cycle frequency for the hopping-robot limb configuration. The snap-through power-stroke duration T_{act} was varied while $T_{\mathrm{reset}} = 0.8$ s and $T_{\mathrm{dwell}} = 0.1$ s were fixed. (B) Normalized swimming speed as a function of limb installation angle ϕ at fixed actuation-cycle frequency. ϕ is defined as the angle between the motor base and the horizontal direction, with counterclockwise rotation taken as positive. The marked hopping configuration corresponds to the limb installation angle used for terrestrial hopping; it enables swimming but is not optimized for aquatic propulsion. (C) Representative swimming snapshots at low and high actuation-cycle frequencies. (D) Representative swimming snapshots for two limb installation angles, showing that limb installation geometry affects aquatic propulsion. These experiments are exploratory and are intended to demonstrate tunability rather than identify a globally optimized swimming design.

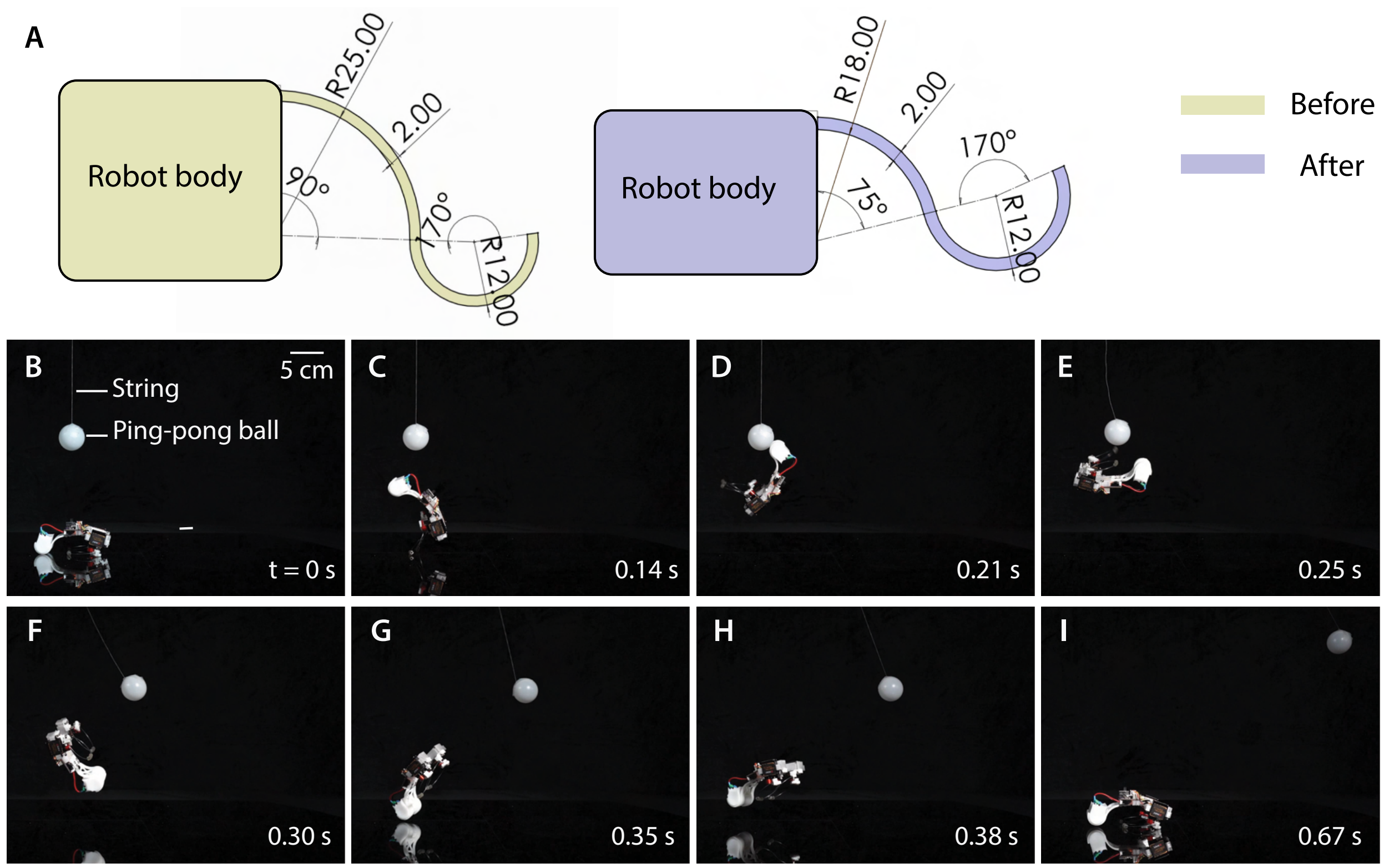

Figure S22: **Updated front-balanced limb design enables consecutive back-flipping.** (A) Side view of the robot with the updated front-balanced limbs (light purple) compared to the previous limbs (light green). Limb design parameters are indicated in the figure (units: mm). (B–I) One back-flip cycle of the robot, showing how the new limbs enable consecutive back-flips to bicycle-kick a hanging ping-pong ball.

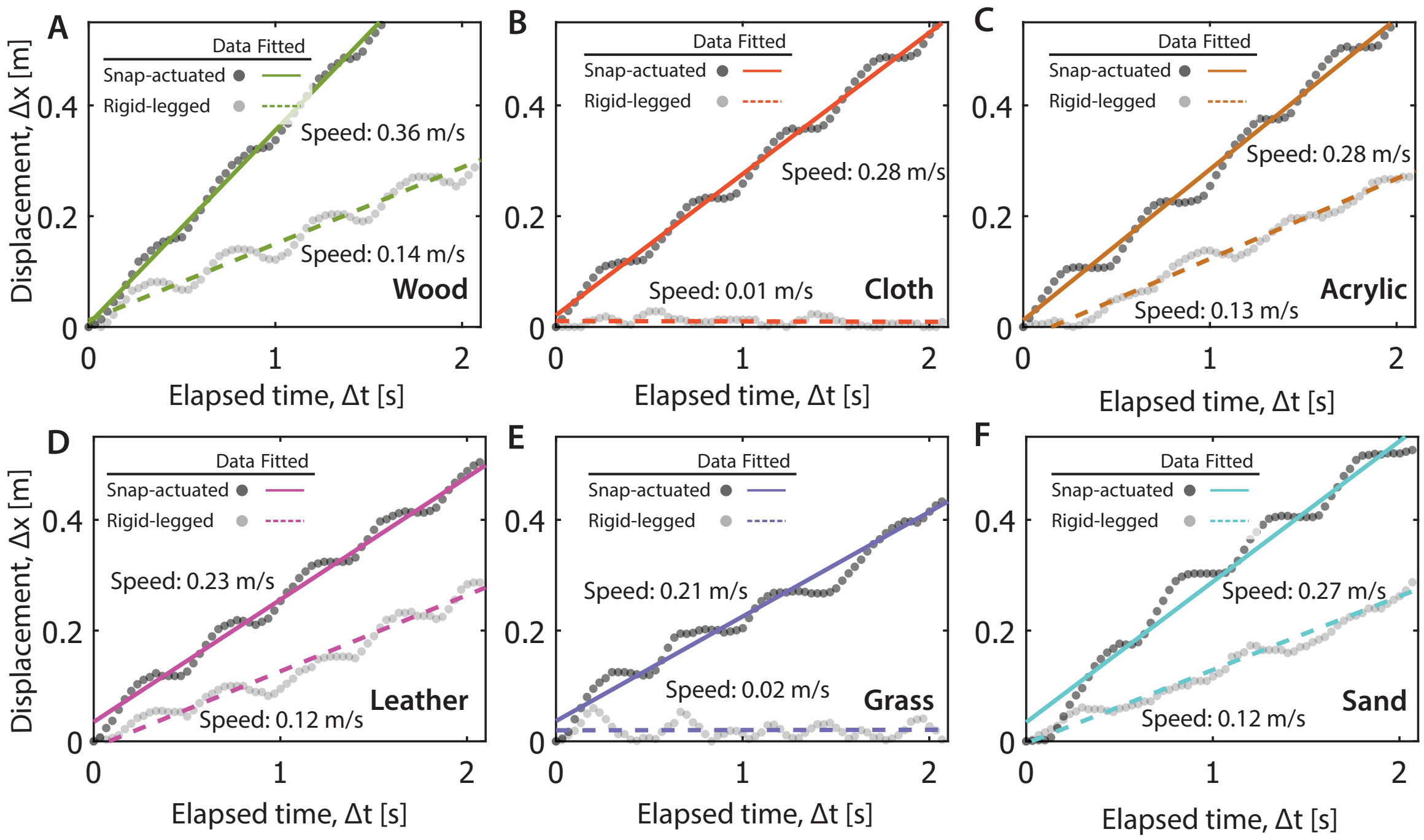

Figure S23: **Tracked robot position over time and corresponding linear fits.** Example datasets of body displacement versus time for the hopping robot on six terrains, showing tracked positions (dots) and linear fits (lines): (A) wood, (B) cloth, (C) acrylic, (D) leather, (E) grass, and (F) sand.

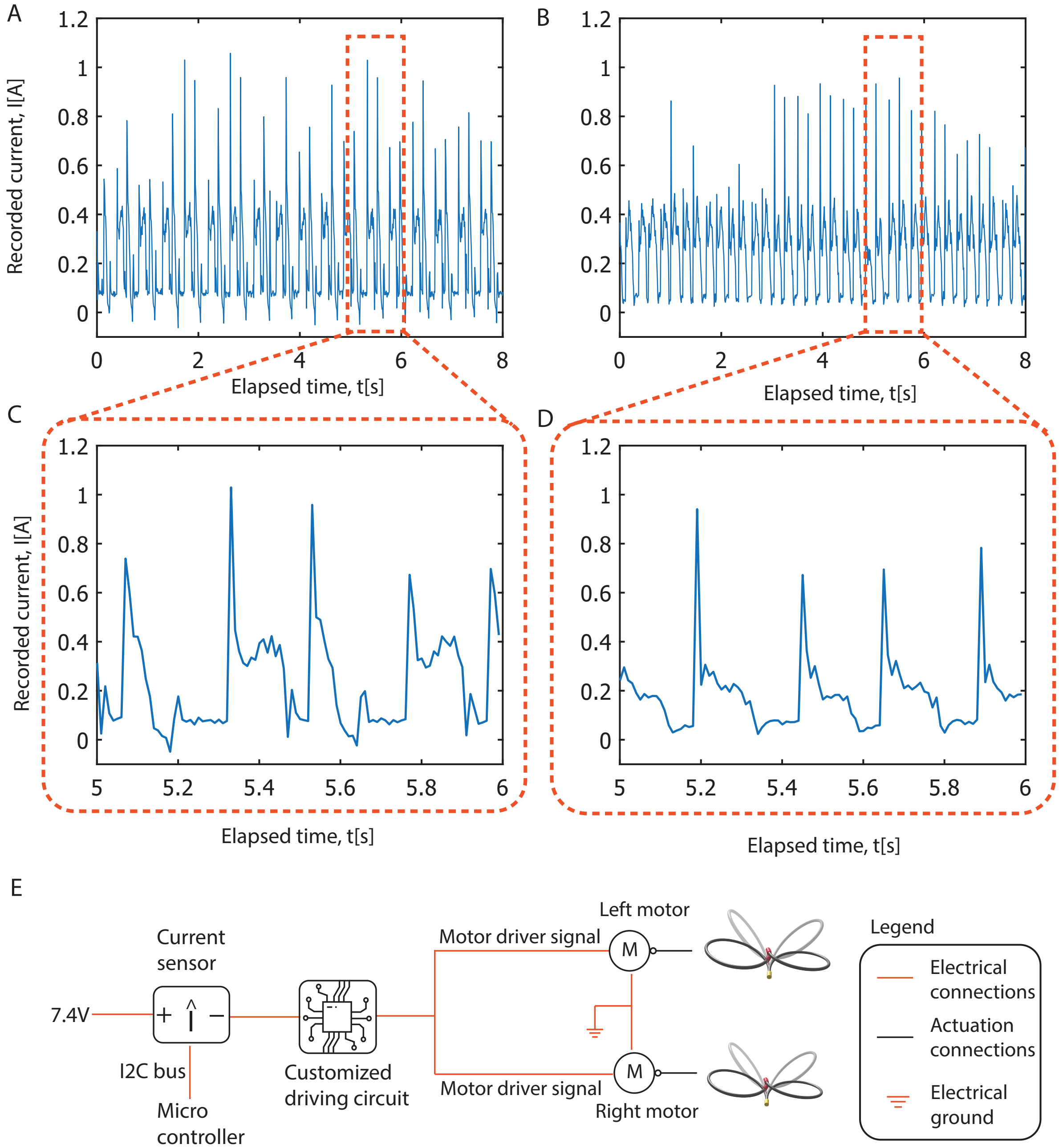

Figure S24: **Cost of transport testing and schematics for untethered robots.** (A) Current drawn of the untethered hopping robot. (B) Current drawn of the untethered rigid-legged robot. (C) Zoomed in view of 1 s period current drawn of the untethered hopping robot. (D) Zoomed in view of 1 s period current drawn of the untethered rigid-legged robot. (E) Schematics of the CoT experimental setup.

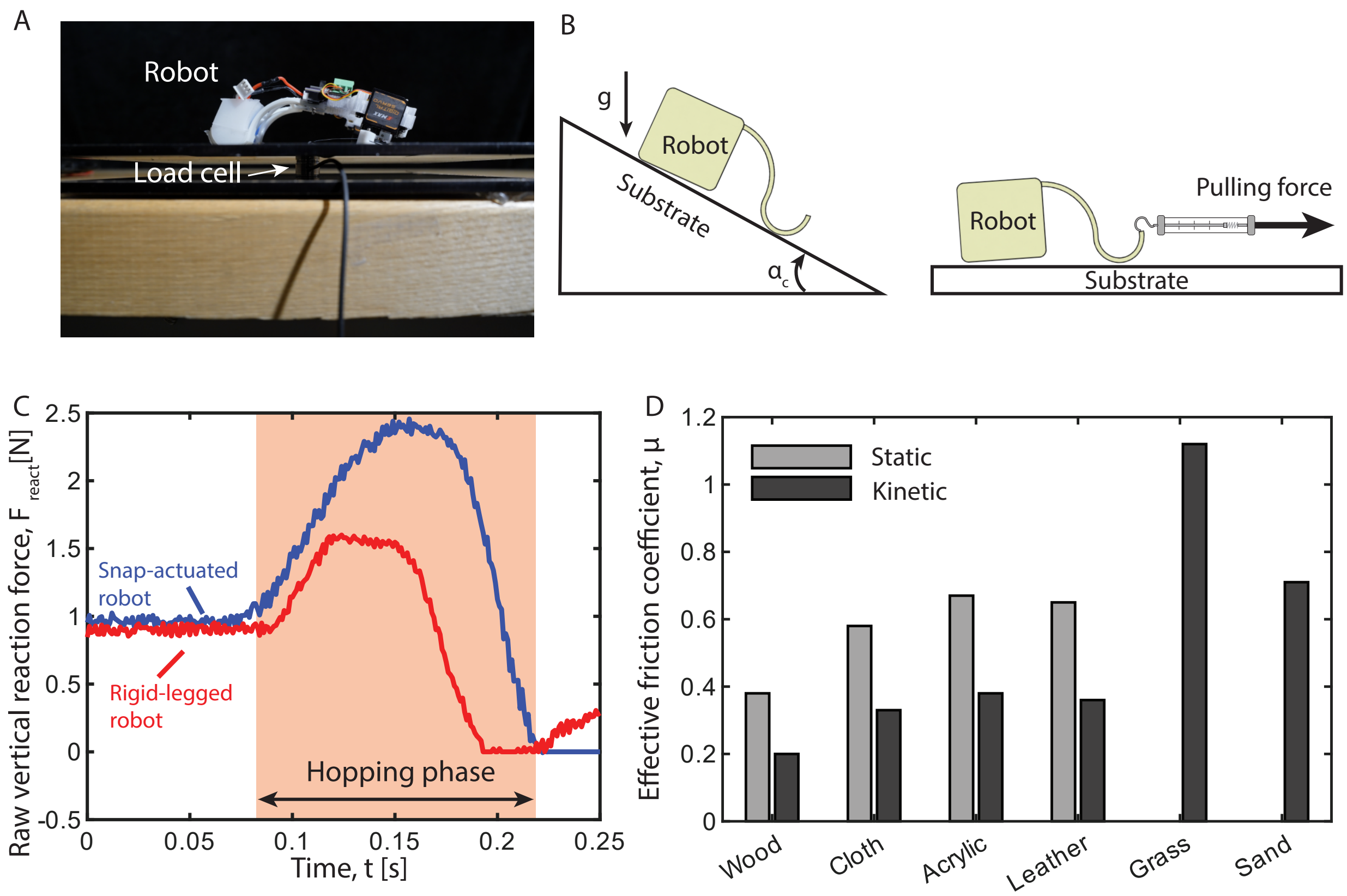

Figure S25: **Robot-level reaction-force and substrate-friction characterization.** (A) Setup for measuring vertical reaction force during robot hopping on an acrylic substrate. (B) Effective robot–substrate friction-characterization methods: static friction from the critical tilt angle and kinetic friction from force-gauge pulling. (C) Representative raw vertical reaction-force traces for the snap-actuated robot and rigid-legged control under matched actuation conditions. The shaded region marks the impulse-integration window. The snap-actuated robot shows a larger and more rapid force increase. (D) Measured effective robot–substrate static and kinetic friction coefficients for the tested substrates.

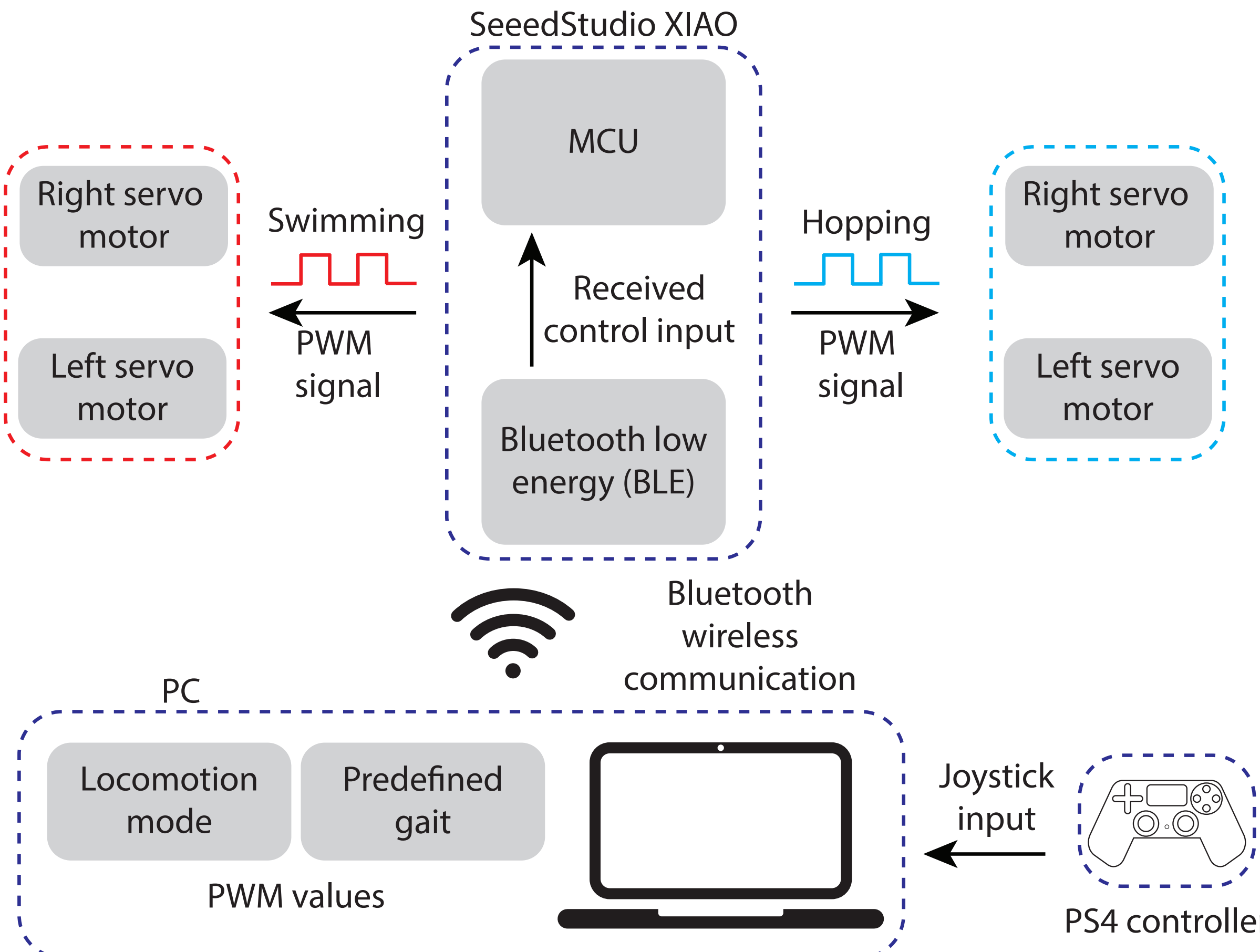

Figure S26: **Control system architecture for the hopping robot.** User commands are provided via a handheld game controller to a host PC, which interprets joystick inputs and locomotion mode selections at 50Hz to generate predefined gait parameters. These commands are wirelessly transmitted via Bluetooth Low Energy to the onboard microcontroller, which executes a dedicated real-time control loop at 200Hz. The microcontroller translates the gait parameters into synchronized pulse-width modulation (PWM) signals, directly driving the left and right servo motors to execute the desired locomotion behavior.

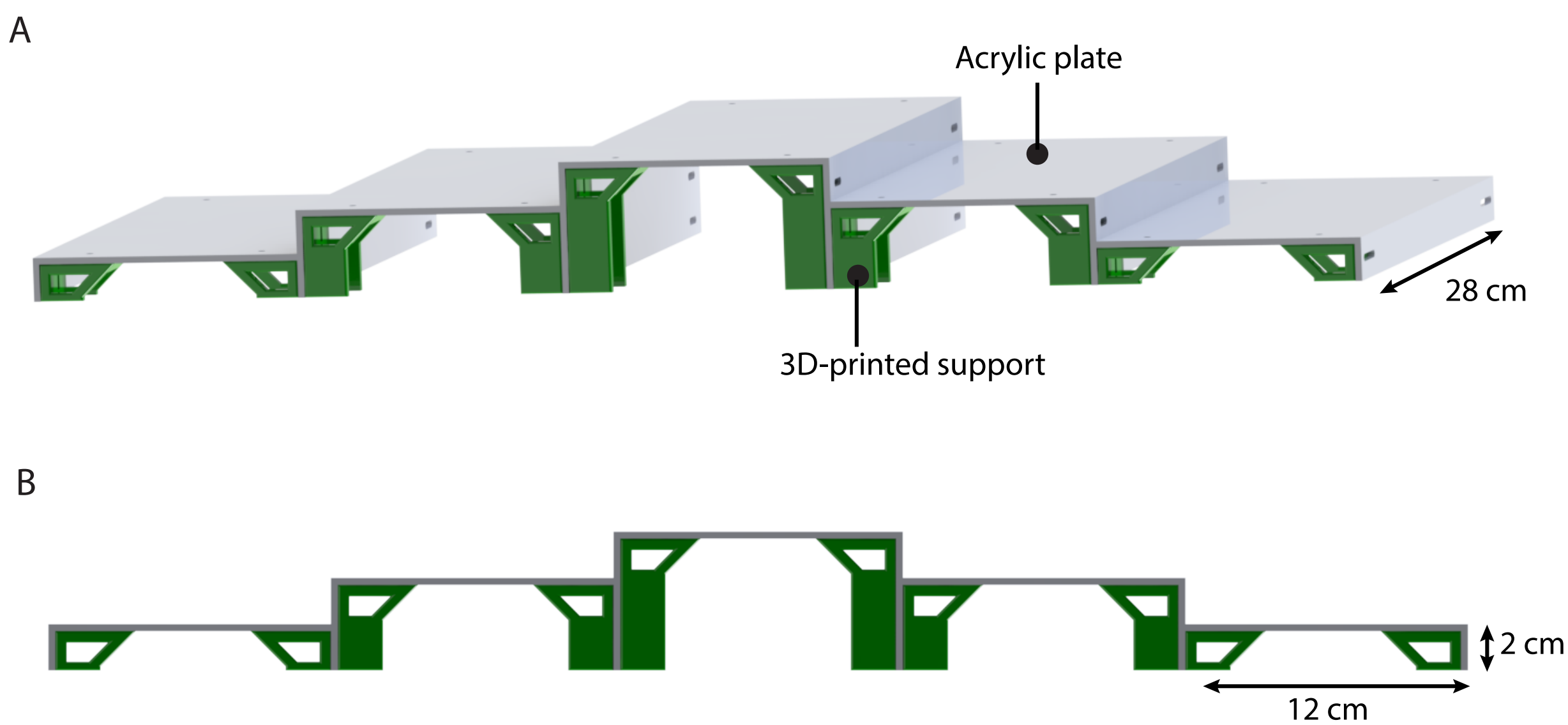

Figure S27: **Design of the constructed stairs** (A) Rendering of the constructed stairs. Key components include the acrylic plates and 3D-printed support. (B) Front view of the constructed stairs.

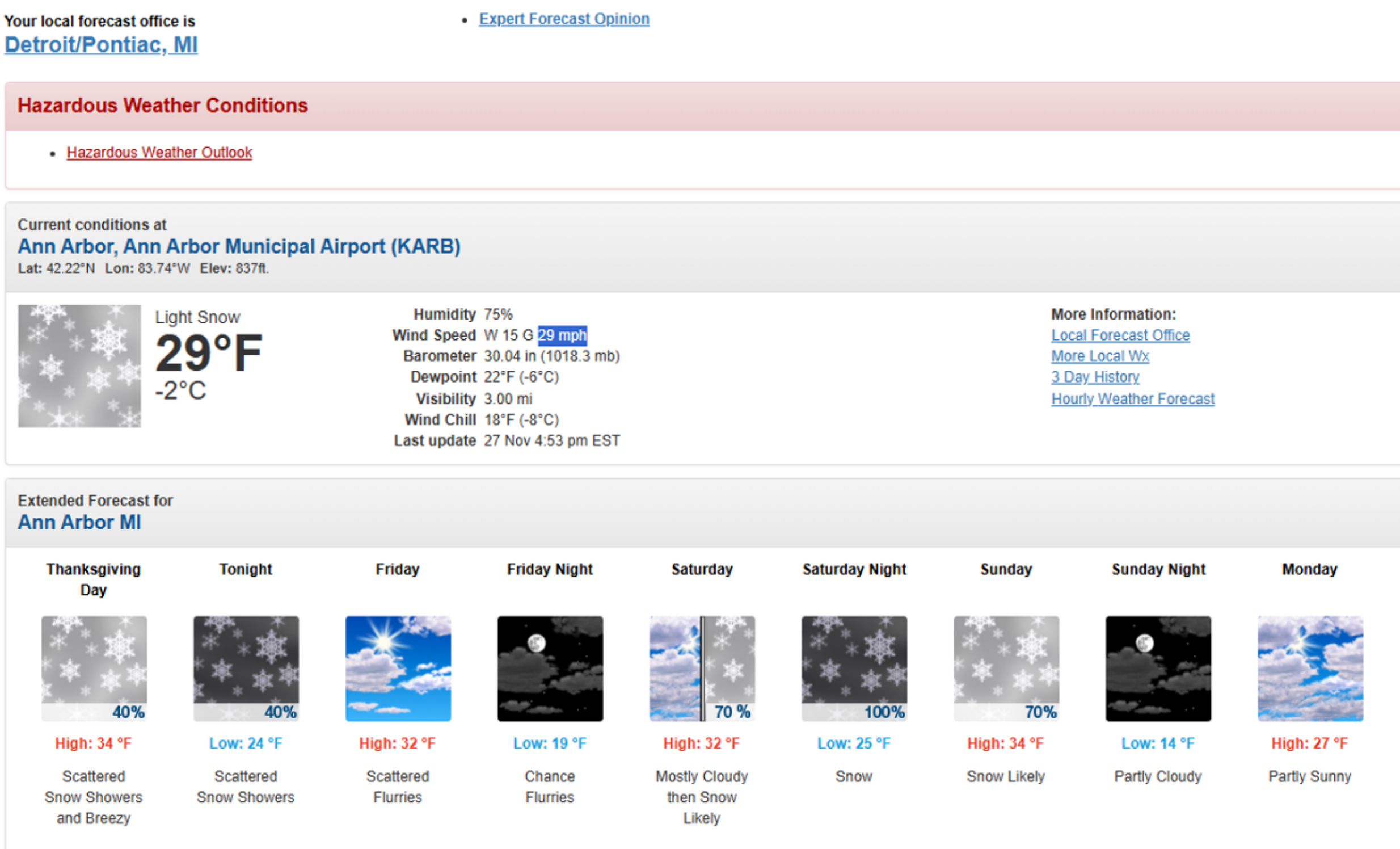

Figure S28: **Weather recording of the swimming test.** The temperature during the test was −2°C, and wind gusts reached 29 mph (> 12 m/s). The weather information was obtained from the National Weather Service.

Table S1: **Controlled parameters for the snap-actuated robot and rigid-legged control used in the terrain comparison.** The two robots used the same platform, command protocol, contact interface, terrain set, and tracking procedure. The only intended design difference was the replacement of the snap-actuated elastic helical limbs with rigid printed counterparts. Electrical quantities were measured under matched command conditions using a fixed 7.4 V supply.

**Parameter**	**Controlled condition or measured value**
Robot platform	Same body architecture, onboard 2S LiPo battery, custom control board, and micro-servos (EMAX ES9052MD HV)
Limb installation geometry	Same holder inclination (10°) and effective limb length from servo output-shaft center to friction-pad ground-contact point (4.3 cm)
Command and actuation	Same prescribed servo command sequence and actuation-cycle frequency, f_{act} = 2.13 Hz
Terrain-test conditions	Same six terrains/substrates as in Fig. 5 and same contact-pad material (Formlabs Elastic 50A Resin V2).
Intended design difference	Snap-actuated elastic helical limbs for the snap-actuated robot; rigid printed counterparts for the rigid-legged control
Total mass	Snap-actuated robot: 98.2 g; rigid-legged control: 92.6 g
Electrical characterization	Snap-actuated robot: average current 0.22 A and average electrical power 1.63 W; rigid-legged control: average current 0.19 A and average electrical power 1.41 W

Table S2: **Friction coefficients for different terrains.** Static and kinetic friction coefficients between the robot and each terrain used in Fig. 5, Fig. S25, and Fig. S23.

Terrain	*Static coefficient* μ_s	*Kinetic coefficient* μ_k
Wood	0.36	0.20
Cloth	0.58	0.33
Acrylic	0.67	0.38
Leather	0.65	0.36
Grass	N/A	1.12
Sand	N/A	0.71

Table S3: **Minimum electrical cost of transport (CoT) of our robots.**

Comparison of the CoT for the untethered hopping robot and the rigid-legged robots across various terrains.

Terrain	*Untethered hopping robot CoT*	*Rigid-legged robot CoT*
Wood	4.79	11.76
Cloth	6.15	141.11
Acrylic	6.19	12.71
Leather	7.37	13.07
Grass	8.06	64.14
Sand	6.06	13.44

Table S4: **Speed and cost of transport (CoT) of state-of-the-art untethered soft robots**

Data correspond to the points reported in Fig. 5H.

Reference	*Speed (BL/s)*	*Speed (mm/s)*	*CoT*	*Length (mm)*	*Able to jump*
Our work on wood	3.21	353.1	4.79	110	Yes
Our work on cloth	2.50	275.1	6.15	110	Yes
Our work on acrylic	2.48	273.3	6.19	110	Yes
Our work on leather	2.09	229.6	7.37	110	Yes
Our work on grass	1.91	210	8.06	110	Yes
Our work on sand	2.54	279	6.06	110	Yes
BHMBot-B (*49*)	18.0	360	237.3	20	No
BHMBot-B (*49*) on sand	0.7	14	5971.4	20	No
UEST PVDF bot (*50*)	4.8	96	117	20	No
Tsinghua PVDF bot 1 (*51*)	1.1	27.2	887	24	No
Tsinghua PVDF bot 2 (*52*)	1.9	45	261	24	No
LVSDEA bot (*53*)	0.3	12	1670	40	No
UW insect bot (*54*)	1.4	2.24	55	54	No
UW insect bot (*55*)	3.8	172	84	45	No
LEONARDO (*56*)	0.27	200	108	750	No
Kink bot (*57*)	1.93	181	11	94	No
HIT LCE bot (*58*)	0.28	17	55	59.6	No
HSA-Quad bot (*59*)	0.65	183	8.35	280	No

Table S5: **Speed and cost of transport (CoT) of mammals**

Data correspond to the points reported in Fig. 5H.

Animals	*Speed (BL/s)*	*Speed (mm/s)*	*CoT*	*Length (mm)*
Mouse (Microtus) (*60, 61*)	1.39	150	40.07	108
Mouse (Clethrionomys) (*60, 61*)	0.95	103	31.26	108
Mouse (Peromyscus) (*60, 61*)	3.16	300	14.94	95
Mouse (Mus) (*60, 61*)	1.67	156	20.88	93
Lemming (Dicrostonyx) (*60, 61*)	3.29	428	17.46	130
Kangaroo rat (Dipodomys) (*60, 61*)	4.86	583	3.92	120
Ground squirrel (Citellus) (*60, 62*)	3.24	778	2.32	240
Rat 1 (Rattus) (*60, 62*)	2.24	583	3.84	260
Rat 2 (Rattus) (*60, 62*)	1.36	300	6.40	220
Rabbit (*60, 62*)	1.30	583	2.03	450
Dog (*60, 63*)	3.24	1622	0.58	500
Sheep (*60, 63*)	0.67	800	0.43	1200
Man (*60, 64*)	0.89	1561	0.31	1750
Pony (*60, 63*)	0.07	98	2.68	1400
Colt (*60, 63*)	0.58	983	0.29	1700
Cattle (*60, 63*)	0.49	983	0.35	2000
Horse (*60, 63*)	0.58	1389	0.22	2400

Table S6: **Speed and cost of transport (CoT) of reptiles**

Data correspond to the points reported in Fig. 5H.

Animals	*Speed (BL/s)*	*Speed (mm/s)*	*CoT*	*Length (mm)*
Lizard (*60, 62*)	0.09	102	2.66	1100
Turtle (*65*)	0.33	100	0.61	300
Snake (*66, 67*)	0.33	140	1.61	419

Movie S1. The buckling of helical filament. Changing the prescribed boundary conditions of a slender elastic filament produces different helical configurations and selects two distinct instability pathways: snap-through and continuous buckling. (i) As the filament is loaded from a stable helical configuration toward an unstable helical configuration, instability occurs. The ideal loading path is defined as a ray in helix-parameter space. (ii) For certain loading directions, the filament undergoes snap-through during the transition from a stable to an unstable helical equilibrium, as demonstrated by automated robotic experiments and numerical simulations. The measured response exhibits hysteresis during loading and unloading. (iii) For other loading directions, the filament undergoes continuous buckling along the same type of transition, again confirmed by experiments and simulations. In this case, the response follows a smooth and reversible evolution during loading and unloading.

Movie S2. Nonlinear snap-through response of the elastic limb. High-speed recordings highlight how snap-through of a helical elastic limb enables rapid reconfiguration and burst-like energy release. (i) Numerical simulations show that the ideal loading path, which combines rotation and translation in the full boundary-condition space, can be approximated using simplified single-degree-of-freedom actuation: rotation about the main axis $\mathbf{e}_1$ is sufficient to trigger snap-through in the selected helical-limb design. (ii) Triggering snap-through in different helical filaments produces different peak reconfiguration speeds and energy releases. Three limbs are fabricated corresponding to critical helical shapes with helix angles $\alpha = 0.01$, $\alpha = 0.11$, and $\alpha = 0.20$. Side-by-side comparisons of experimental and simulated snapshots, together with measured kinematics, show that increasing α produces higher midpoint peak velocities but also requires longer actuation times. Videos are played back in 100× slow motion. (iii) After the limb reconfigures via snap-through, it can be reset to its original helical state by reversing the rotation direction. Larger-helix-angle limbs require longer reset times. Videos are played back in 100× slow motion. (iv) A ping-pong ball impact test demonstrates that the elastic limb can use snap-through-amplified motion to deliver stronger impacts than a rigid leg of comparable size. Videos are shown in real time.

Movie S3. Repeatability test of the snap-actuated helical limb. A snap-actuated helical limb completed 1200 consecutive snap-through/reset cycles under the prescribed command protocol,

with no observed failures. The first 10 s and last 10 s are shown in real time, and the intermediate segment is played back at 10× speed.

Movie S4. Untethered snap-through-actuated robot with multimodal locomotion. The untethered robot, powered by two elastic limbs actuated through helical snap-through, achieves fast and agile locomotion including hopping, turning, swimming, and back-flipping. (i) The untethered robot carries its own energy source, a lithium-polymer battery, and two mini servos. It moves forward using a hopping gait. The main sequence is shown in real time. A 16× slow-motion clip highlights a single hop cycle: the limbs snap through to kick off, reset to their helical shape during flight, and land to initiate the next hop. (ii) By actuating only one limb while allowing the other limb to reset passively, the robot executes a rapid turning gait. The video is shown in real time. (iii) After waterproofing the robot and adding a thin film to each limb to form flipper-like feet, the robot adopts an amphibious swimming gait and can propel and steer itself in water. The video is shown in real time. (iv) By adjusting the balancing limb design to shift the robot's center of mass, the robot can perform consecutive back-flips for rapid and agile motion. The video is shown in real time, followed by a 10× slow-motion segment showing one complete back-flip. (v) The robot performs a "bicycle kick" to strike a ping-pong ball using its snap-through-driven elastic limbs. The video is shown in real time. (vi) The effect of actuation-cycle frequency f_{act} on robot hopping performance is shown.

Movie S5. Comparison of snap-actuated elastic-limb and rigid-legged robots. Two fully untethered robots are compared across different terrestrial terrains: one powered by helical snap-through elastic limbs and an otherwise identical control robot with rigid legs. (i) Exploded-view CAD images show the designs of both robots. Only the limb design differs; all other major components, including the battery, electronics, and chassis, are kept the same. (ii) On wood, the snap-actuated robot reaches 3.21 body lengths per second (BL/s), whereas the rigid-legged control reaches 1.20 BL/s. (iii) On cloth, the snap-actuated robot reaches 2.50 BL/s, whereas the rigid-legged control reaches 0.10 BL/s. (iv) On acrylic, the snap-actuated robot reaches 2.48 BL/s, whereas the rigid-legged control reaches 1.11 BL/s. (v) On leather, the snap-actuated robot reaches 2.09 BL/s, whereas the rigid-legged control reaches 1.08 BL/s. (vi) On grass, the snap-actuated

robot reaches 1.91 BL/s, whereas the rigid-legged control reaches 0.22 BL/s. (vii) On sand, the snap-actuated robot reaches 2.54 BL/s, whereas the rigid-legged control reaches 1.05 BL/s. All videos are shown in real time.

Movie S6. Ascending and descending stairs via hopping locomotion. The untethered robot climbs up and down a staircase, demonstrating its ability to negotiate step-like obstacles.

Movie S7. Teleoperated path-following locomotion on sandy and rocky terrain. The untethered robot navigates an outdoor sand playground, following a prescribed trajectory under teleoperation to demonstrate controlled locomotion in an unstructured environment. The experiment is conducted in the Mars Yard at the Robotics Department of the University of Michigan. Cones mark the desired path, and the untethered robot follows the trajectory over a distance of approximately 10 m. The video is shown in real time.

Movie S8. Wind-disturbance swimming demo in a natural pool. The untethered robot swims in a natural outdoor pool, using its elastic limbs to propel and steer in an unstructured aquatic environment under windy conditions. The experiments are conducted at Olson Park in Ann Arbor, Michigan, USA. (i) The robot follows a U-shaped trajectory to navigate around an underwater stick obstacle. (ii) The robot circles around an underwater obstacle, demonstrating agile turning in water. Both videos are played back at 3× real-time speed.

Movie S9. Simple light-guided steering using onboard light-dependent resistors (LDRs). The untethered robot uses two onboard light-dependent resistors (LDRs) to steer toward a light source. The robot starts from different positions and orientations and reaches the illuminated target area. Both videos are played back at 1.5× real-time speed.

REFERENCES

1. M. Gomez, D. E. Moulton, D. Vella, Critical slowing down in purely elastic ‘snap-through’ instabilities. *Nat. Phys.* **13**, 142–145 (2017).

2. A. Pal, V. Restrepo, D. Goswami, R. V. Martinez, Exploiting mechanical instabilities in soft robotics: Control, sensing, and actuation. *Adv. Mater.* **33**, 2006939 (2021).

3. M. Ilton, M. S. Bhamla, X. Ma, S. M. Cox, L. L. Fitchett, Y. Kim, J. Koh, D. Krishnamurthy, C.-Y. Kuo, F. Z. Temel, A. J. Crosby, M. Prakash, G. P. Sutton, R. J. Wood, E. Azizi, S. Bergbreiter, S. N. Patek, The principles of cascading power limits in small, fast biological and engineered systems. *Science* **360**, eaao1082 (2018).

4. R. M. Alexander, Tendon elasticity and muscle function. *Comp. Biochem. Physiol. A Mol. Integr. Physiol.* **133**, 1001–1011 (2002).

5. J. T. B. Overvelde, T. Kloek, J. J. A. D’haen, K. Bertoldi, Amplifying the response of soft actuators by harnessing snap-through instabilities. *Proc. Natl. Acad. Sci. U. S. A.* **112**, 10863–10868 (2015).

6. G. H. M. Van Der Heijden, J. M. T. Thompson, Helical and localised buckling in twisted rods: A unified analysis of the symmetric case. *Nonlinear Dyn.* **21**, 71–99 (2000).

7. B. Audoly, Y. Pomeau, *Elasticity and Geometry: From Hair Curls to the Non-linear Response of Shells* (Oxford Univ. Press, 2010).

8. S. S. Antman, *Nonlinear Problems of Elasticity* (Springer, ed. 2, 2005).

9. W. Huang, M. K. Jawed, Numerical exploration on buckling instability for directional control in flagellar propulsion. *Soft Matter* **16**, 604–613 (2020).

10. A. Lazarus, J. T. Miller, P. M. Reis, Continuation of equilibria and stability of slender elastic rods using an asymptotic numerical method. *J. Mech. Phys. Solids* **61**, 1712–1736 (2013).

11. Q. Wang, A. Giudici, W. Huang, Y. Wang, M. Liu, S. Tawfick, D. Vella, Transient amplification of broken symmetry in elastic snap-through. *Phys. Rev. Lett.* **132**, 267201 (2024).

12. W. Huang, T. Yu, D. Vella, K. J. Hsia, M. Liu, Exploiting dynamic bifurcation in elastic ribbons for mode skipping and selection. *J. Mech. Phys. Solids* **190**, 105721 (2024).

13. H. Fu, K. Nan, W. Bai, W. Huang, K. Bai, L. Lu, C. Zhou, Y. Liu, F. Liu, J. Wang, M. Han, Z. Yan, H. Luan, Y. Zhang, Y. Zhang, J. Zhao, X. Cheng, M. Li, J. W. Lee, Y. Liu, D. Fang, X. Li, Y. Huang, Y. Zhang, J. A. Rogers, Morphable 3D mesostructures and microelectronic devices by multistable buckling mechanics. *Nat. Mater.* **17**, 268–276 (2018).

14. N. Chouaieb, A. Goriely, J. H. Maddocks, Helices. *Proc. Natl. Acad. Sci. U. S. A.* **103**, 9398–9403 (2006).

15. J. Aguilar, T. Zhang, F. Qian, M. Kingsbury, B. McInroe, N. Mazouchova, C. Li, R. Maladen, C. Gong, M. Travers, R. L. Hatton, H. Choset, P. B. Umbanhowar, D. I. Goldman, A review on locomotion robophysics: The study of movement at the intersection of robotics, soft matter and dynamical systems. *Rep. Prog. Phys.* **79**, 110001 (2016).

16. Y. Chen, P. Chirarattananon, K. Jayaram, A call for a performance-driven approach for soft robotics research. *npj Robot* **4**, 14 (2026).

17. H. C. Astley, A. Haruta, T. J. Roberts, Robust jumping performance and elastic energy recovery from compliant perches in tree frogs. *J. Exp. Biol.* **218**, 3360–3363 (2015).

18. D. Tang, C. Zhang, C. Pan, H. Hu, H. Sun, H. Dai, J. Fu, C. Majidi, P. Zhao, Bistable soft jumper capable of fast response and high takeoff velocity. *Sci. Robot.* **9**, eadm8484 (2024).

19. Q. Guo, Y. Sun, T. Zhang, S. Xie, X. Chen, Z. Zhang, H. Jiang, L. Yang, Bistable insect-scale jumpers with tunable energy barriers for multimodal locomotion. *Adv. Sci.* **11**, e2404404 (2024).

20. Y. Tang, Y. Chi, J. Sun, T.-H. Huang, O. H. Maghsoudi, A. Spence, J. Zhao, H. Su, J. Yin, Leveraging elastic instabilities for amplified performance: Spine-inspired high-speed and high-force soft robots. *Sci. Adv.* **6**, eaaz6912 (2020).

21. D. Drotman, S. Jadhav, D. Sharp, C. Chan, M. T. Tolley, Electronics-free pneumatic circuits for controlling soft-legged robots. *Sci. Robot.* **6**, eaay2627 (2021).

22. Q. Ze, S. Wu, J. Nishikawa, J. Dai, Y. Sun, S. Leanza, C. Zemelka, L. S. Novelino, G. H. Paulino, R. R. Zhao, Soft robotic origami crawler. *Sci. Adv.* **8**, eabm7834 (2022).

23. H. Qing, J. Guo, Y. Zhu, Y. Chi, Y. Hong, D. Quinn, H. Dong, J. Yin, Spontaneous snapping-induced jet flows for fast, maneuverable surface and underwater soft flapping swimmer. *Sci. Adv.* **10**, eadq4222 (2024).

24. Y. Chi, Y. Hong, Y. Zhao, Y. Li, J. Yin, Snapping for high-speed and high-efficient butterfly stroke–like soft swimmer. *Sci. Adv.* **8**, eadd3788 (2022).

25. P. Yang, Y. Mao, H. Liu, L. Gao, F. Huang, F. Dang, A rolling soft robot driven by local snap-through buckling. *Soft Robot.*,10.1089/soro.2024.0115 (2024).

26. X. Yang, Y. Zhou, H. Zhao, W. Huang, Y. Wang, K. J. Hsia, M. Liu, Morphing matter: From mechanical principles to robotic applications. *Soft Sci.* **3**, 38 (2023).

27. Y. Wang, Q. Wang, M. Liu, Y. Qin, L. Cheng, O. Bolmin, M. Alleyne, A. Wissa, R. H. Baughman, D. Vella, S. Tawfick, Insect-scale jumping robots enabled by a dynamic buckling cascade. *Proc. Natl. Acad. Sci. U. S. A.* **120**, e2210651120 (2023).

28. B. Gorissen, D. Melancon, N. Vasios, M. Torbati, K. Bertoldi, Inflatable soft jumper inspired by shell snapping. *Sci. Robot.* **5**, eabb1967 (2020).

29. B. Treml, A. Gillman, P. Buskohl, R. Vaia, Origami mechanologic. *Proc. Natl. Acad. Sci. U. S. A.* **115**, 6916–6921 (2018).

30. M. Bergou, M. Wardetzky, S. Robinson, B. Audoly, E. Grinspun, Discrete elastic rods. *ACM Trans. Graph.* **27**, 63 (2008).

31. M. Bergou, B. Audoly, E. Vouga, M. Wardetzky, E. Grinspun, Discrete viscous threads. *ACM Trans. Graph.* **29**, 1–10 (2010).

32. W. Huang, Z. Hao, J. Li, D. Tong, K. Guo, Y. Zhang, H. Gao, K. J. Hsia, M. Liu, A tutorial on simulating nonlinear behaviors of flexible structures with the discrete differential geometry (DDG) method. *Appl. Mech. Rev.* **78**, 064801 (2026).

33. S. Edwards, J. Nicho, J. Meyer, "The descartes planning library for semi-constrained cartesian trajectories," in *ROSCon Hamburg 2015* (Open Robotics, 2015; https://vimeo.com/142622435).

34. D. Tong, Geometry-controlled instability pathway selection in elastic helices enables fast, efficient robotic locomotion, Zenodo, version 1 (2025); https://doi.org/10.5281/ZENODO.18064935.

35. A. Borum, T. Bretl, When is a helix stable? *Phys. Rev. Lett.* **125**, 088001 (2020).

36. A. Borum, T. Bretl, Infinitely long isotropic Kirchhoff rods with helical centerlines cannot be stable. *Phys. Rev. E* **102**, 023004 (2020).

37. E. L. Allgower, K. Georg, *Introduction to Numerical Continuation Methods* (Society for Industrial and Applied Mathematics, 2003).

38. A. Choi, D. Tong, M. K. Jawed, J. Joo, Implicit contact model for discrete elastic rods in knot tying. *J. Appl. Mech.* **88**, 051010 (2021).

39. D. Tong, A. Choi, J. Joo, A. Borum, M. K. Jawed, Snap buckling in overhand knots. *J. Appl. Mech.* **90**, 041008 (2023).

40. M. K. Jawed, F. Da, J. Joo, E. Grinspun, P. M. Reis, Coiling of elastic rods on rigid substrates. *Proc. Natl. Acad. Sci. U. S. A.* **111**, 14663–14668 (2014).

41. D. Tong, A. Choi, L. Qin, W. Huang, J. Joo, M. K. Jawed, Sim2Real neural controllers for physics-based robotic deployment of deformable linear objects. *Int. J. Robot. Res.* **43**, 791–810 (2024).

42. M. K. Jawed, N. K. Khouri, F. Da, E. Grinspun, P. M. Reis, Propulsion and instability of a flexible helical rod rotating in a viscous fluid. *Phys. Rev. Lett.* **115**, 168101 (2015).

43. D. Tong, A. Choi, J. Joo, M. K. Jawed, A fully implicit method for robust frictional contact handling in elastic rods. *Extreme Mech. Lett.* **58**, 101924 (2023).

44. W. Chen, D. Tong, L. Meng, B. Tan, R. Lan, Q. Zhang, H. Yang, C. Wang, K. Liu, Knotted artificial muscles for bio-mimetic actuation under deepwater. *Adv. Mater.* **36**, e2400763 (2024).

45. W. Huang, X. Huang, C. Majidi, M. K. Jawed, Dynamic simulation of articulated soft robots. *Nat. Commun.* **11**, 2233 (2020).

46. W. Kabsch, A solution for the best rotation to relate two sets of vectors. *Acta Cryst. A* **32**, 922–923 (1976).

47. W. Huang, T. Yu, K. J. Hsia, S. Adriaenssens, M. Liu, Integration of kinks and creases enables tunable folding in meta-ribbons. *Matter* **7**, 3007–3023 (2024).

48. A. Mohammadgholipour, A. M. Billah, Mechanical properties and constitutive models of shape memory alloy for structural engineering: A review. *J. Intell. Mater. Syst. Struct.* **34**, 2335–2359 (2023).

49. X. Yu, W. Zhan, Z. Liu, L. Wei, W. Shen, R. Yun, J. Leng, H. Xu, M. Qi, X. Yan, Forward and backward control of an ultrafast millimeter-scale microrobot via vibration mode transition. *Sci. Adv.* **10**, eadr1607 (2024).

50. Y. Wu, L. Cao, G. Lu, P. Wang, L. Ran, B. Peng, Untethered soft microrobot driven by a single actuator for agile navigations. *Nat. Commun.* **16**, 6842 (2025).

51. J. Liang, Y. Wu, J. K. Yim, H. Chen, Z. Miao, H. Liu, Y. Liu, Y. Liu, D. Wang, W. Qiu, Z. Shao, M. Zhang, X. Wang, J. Zhong, L. Lin, Electrostatic footpads enable agile insect-scale soft robots with trajectory control. *Sci. Robot.* **6**, eabe7906 (2021).

52. Z. Miao, J. Liang, H. Chen, J. Lu, X. Sun, Y. Liu, F. Tang, M. Zhang, Power autonomy and agility control of an untethered insect-scale soft robot. *Soft Robot.* **10**, 749–759 (2023).

53. X. Ji, X. Liu, V. Cacucciolo, M. Imboden, Y. Civet, A. El Haitami, S. Cantin, Y. Perriard, H. Shea, An autonomous untethered fast soft robotic insect driven by low-voltage dielectric elastomer actuators. *Sci. Robot.* **4**, eaaz6451 (2019).

54. V. Iyer, A. Najafi, J. James, S. Fuller, S. Gollakota, Wireless steerable vision for live insects and insect-scale robots. *Sci. Robot.* **5**, eabb0839 (2020).

55. B. Goldberg, R. Zufferey, N. Doshi, E. F. Helbling, G. Whittredge, M. Kovac, R. J. Wood, Power and control autonomy for high-speed locomotion with an insect-scale legged robot. *IEEE Robot. Autom. Lett.* **3**, 987–993 (2018).

56. K. Kim, P. Spieler, E.-S. Lupu, A. Ramezani, S.-J. Chung, A bipedal walking robot that can fly, slackline, and skateboard. *Sci. Robot.* **6**, eabf8136 (2021).

57. A. Comoretto, H. A. H. Schomaker, J. T. B. Overvelde, Physical synchronization of soft self-oscillating limbs for fast and autonomous locomotion. *Science* **388**, 610–615 (2025).

58. Y. Xia, T. Mu, J. Guo, Y. Liu, J. Leng, Multifunctional untethered soft machines driven by 4D printed electrically responsive actuators. *ACS Appl. Mater. Interfaces* **17**, 36059–36068 (2025).

59. T. Kim, A. Torres-Soto, H. Wang, R. L. Truby, Architected elastomers as load bearing actuators for untethered soft robot walking. *Adv. Mater. Technol.* **10**, e00503 (2025).

60. V. A. Tucker, Energetic cost of locomotion in animals. *Comp. Biochem. Physiol.* **34**, 841–846 (1970).

61. Plazi, TreatmentBank, https://treatment.plazi.org/.

62. Animal Diversity Web, University of Michigan Museum of Zoology, https://animaldiversity.org/.

63. C. Zaragoza, C. Gomez-Guerrero, J. L. Martin-Ventura, L. Blanco-Colio, B. Lavin, B. Mallavia, C. Tarín, S. Mas, A. Ortiz, J. Egido, Animal models of cardiovascular diseases. *J. Biomed. Biotechnol.* **2011**, 497841 (2011).

64. M. de Onis, A. W. Onyango, E. Borghi, A. Siyam, C. Nishida, J. Siekmann, Development of a WHO growth reference for school-aged children and adolescents. *Bull. World Health Organ.* **85**, 660–667 (2007).

65. R. V. Baudinette, A. M. Miller, S. P. Sarre, Aquatic and terrestrial locomotory energetics in a toad and a turtle: A search for generalisations among ectotherms. *Physiol. Biochem. Zool.* **73**, 672–682 (2000).

66. A. D. Kuo, Choosing your steps carefully. *IEEE Robot. Autom. Mag.* **14**, 18–29 (2007).

67. S. M. Secor, B. C. Jayne, A. F. Bennett, Locomotor performance and energetic cost of sidewinding by the snake Crotalus cerastes. *J. Exp. Biol.* **163**, 1–14 (1992).
